# Evaluation of Cadmium, Lead, Chromium, and Nickel Content in Various Types of Nuts: Almonds, Cashews, Hazelnuts, Peanuts, and Walnuts – Health Risk of Polish Consumers

**DOI:** 10.1007/s12011-024-04438-4

**Published:** 2024-11-06

**Authors:** Małgorzata Ćwieląg-Drabek, Joanna Nieć-Leśniak, Agnieszka Białek-Dratwa, Agata Piekut, Agata Kiciak, Grzegorz Dziubanek, Elżbieta Szczepańska

**Affiliations:** 1 Department of Environmental Health Risk Factors, Faculty of Public Health in Bytom, Medical University of Silesia in Katowice (Poland), 18 Piekarska Street, 41-902 Bytom, Poland; 2 Department of Human Nutrition, Faculty of Public Health in Bytom, Medical University of Silesia in Katowice (Poland), 19 Jordana Street, 41-808 Zabrze, Poland; 3 Department of Environmental Health, Faculty of Public Health in Bytom, Medical University of Silesia in Katowice (Poland), 18 Piekarska Street, 41-902 Bytom, Poland; 4 Department of Food Technology and Quality Evaluation, Faculty of Public Health in Bytom, Medical University of Silesia in Katowice (Poland), 19 Jordana Street, 41-808 Zabrze, Poland

**Keywords:** Heavy metals, Nuts, Dietary intake, Health risk assessment, ETAAS

## Abstract

World production and consumption of nuts continue to rise year on year. Nuts, due to their high nutrient content, are becoming increasingly popular among consumers. A potential health risk may result from the consumption of nuts contaminated with metallic trace elements like cadmium or lead. In this research, we measured the content of Cd, Pb, Cr, and Ni in various edible nuts. All elements were detected using the electrothermal atomic absorption spectrometry analytical technique (ETAAS). The study material comprised 69 nut samples including almonds, cashews, hazelnuts, peanuts, and walnuts available on the Polish market. Indicators such as the hazard quotient (HQ) and hazard index (HI) were used to assess the health risk of consumers. The concentrations of elements in all analyzed samples of nuts were as follows: Cd: peanuts > almonds > hazelnuts > walnuts > cashews, Pb: peanuts > almonds > hazelnuts > walnuts > cashews, Ni: cashews > peanuts > walnuts > hazelnuts > almonds, Cr: almonds > cashews & hazelnuts > peanuts > walnuts. Health risk indicators estimated for consumers whose diet is rich in nuts, in which metallic trace elements were recorded, indicate that an unacceptable impact may occur, and the risk of negative health effects (non-cancer) will be moderate to high.

**Supplementary Information:**

The online version contains supplementary material available at 10.1007/s12011-024-04438-4.

## Introduction

World production and consumption of nuts continue to rise year on year. According to the International Nut and Dried Fruit Council [1] during the 2020/2021 season, over 5.3 million metric tons (MT) of tree nuts were produced worldwide, up 15% from 2019/20 and 65% higher than a decade earlier. Within the tree nut category, the almond production volume (kernel basis) was by far the highest in the given period: over 1.6 mln MT of almonds were produced that year. Walnuts and cashews, which stood in second and third place respectively within the same measured period, each reached over 1 mln MT. During this period, the leading country in terms of global walnut production was China. The United States ranked second, producing just about 600,000 MT. China was also the largest producer of peanuts worldwide, with a global production share of roughly 40% in 2019. India produced about 14% of peanuts. In 2019, the world’s leading exporter of edible nuts was by far the United States, which exported close to 1.4 bln kg that year. Turkey accounted for the second-largest edible nut export volume, which was about 260 mln kg. Other major nut exporters in 2019 included Indonesia and Chile. On the other hand, 2019’s leading importer of edible nuts was Germany. In 2020/2021, world peanut production was estimated at over 47.6 mln MT (in-shell basis), a 7% rise over the previous season and a 33% increase over 2011/2012. Almonds and walnuts were the largest commodities, accounting for 31% and 19% of the global share, respectively, followed by pistachios (19%), cashews (16%), and hazelnuts (10%). Almonds and walnuts accounted for 50% of total tree nut estimated consumption worldwide in 2019 (30% and 20%, respectively), followed by cashews and pistachios (18% and 15%, respectively). Following Europe as the leading consumer, Asia and North America were the 2nd and 3rd largest consuming regions [1].

Nuts, due to their high content of nutrients, are becoming more and more popular among Polish consumers. They are most often eaten as snacks or as additives used in main courses, salads, desserts, cakes, ice cream, and pastries, as well as in the form of spreads. Nuts are included in the group of fruits, where the edible core is enclosed in a woody shell. The most popular edible nuts are almonds (*Prunus Dulcis* (Mill.) D.A.Webb), hazelnuts (*Corylus avellana* L.), walnuts (*Juglans regia* L.), cashews (*Anacardium occidentale)*. Other popular edible nuts are pistachios (*Pistacia* L.), pecans (*Carya illinoinensis* (Wangenh.) K.Koch), macadamia nuts (*Macadamia* F. v. Mueller) and Brazil nuts (*Bertholletia excelsa*). This group of products also includes goober peas, also known as peanuts (*Arachis hypogaea* L.), which, according to the botanical classification, belong to legumes [2].

### Nutritional Properties of Nuts

Nuts, apart from vegetable oils, are natural products rich in fat (43.9–75.8 g/100 g of product), and at the same time provide a large amount of energy (553–718 kcal/100 g of product). The composition of fat consists mainly of monounsaturated fatty acids (MUFA) and polyunsaturated fatty acids (PUFA)—incl. oleic acid and α-linolenic acid (ALA); the content of saturated fatty acids (SFA) is low—only 4–16%. As for walnuts, it should be emphasized that they are the products with the highest ALA content among all edible plants [3]. The products in question are also a rich source of protein (about 25%), characterized by a high content of L-arginine—an amino acid that is a precursor of nitric oxide (NO)—an endogenous vasodilator. The effect of L-arginine is i.e. improvement of blood supply and oxygenation of skeletal muscles, which is especially desirable in athletes, therefore it is recommended to enrich the diet, especially in this population group, with products containing this amino acid [4]. The carbohydrate content in nuts is low (12–30%), the highest of which is in cashews, pistachios, and almonds – 30, 27, and 22% respectively [4]. Nuts are a good source of dietary fiber, minerals (calcium, magnesium, iron, zinc, potassium, phosphorus), vitamins E and K, folic acid, and phenolic compounds [3]. A wide spectrum of the present macro- and microelements classifies them as products with a particularly favorable nutritional profile [3].

### Positive Health Effects of Nut Consumption

The positive impact of nut consumption on human health has been confirmed in many epidemiological studies, where the consumption of nuts was associated, among others, with a reduced incidence of cardiovascular diseases, hypertension, type 2 diabetes, and obesity, as well as a reduction in LDL cholesterol and triglycerides [3, 5–7]. The antioxidants present in nuts reduce the number of oxidized forms of LDL and DNA damage dependent on oxidative stress and also reduce the level of inflammatory markers, such as adhesive proteins (ICAM-1, VCAM-1), interleukin 6, CRP, and E-selectin. Alpha-linolenic acid (ALA), included in the omega-3 acids, in combination with polyphenols, vitamin E and L-arginine improves the reactivity and elasticity of the endothelium damaged by saturated fatty acids. The above-mentioned factors contribute to the improvement of blood circulation in the peripheral vessels, and the reduction of systolic and diastolic pressure, which classifies nuts as a dietary component that may constitute an element of primary and secondary prevention of cardiovascular diseases [6]. Omega-3 acids present in nuts increase the body’s immune resistance associated with the increase in the synthesis of PGE prostaglandins, they also show hypolipemic, anticoagulant, anti-inflammatory, vasodilating effects, and have a positive effect on memory processes and motor functions [6]. Due to the high content of compounds with strong antioxidant properties (vitamin E, selenium, phenolic compounds), nuts should be part of the diet helping to neutralize free radicals. In studies aimed at determining the antioxidant capacity (TAC – Total Antioxidant Capacity), pecans, walnuts, hazelnuts, and pistachios had the strongest free radical scavenging properties [8].

### Heavy Metals in Nuts

Cereal products, vegetables, and fruits – including nuts, grown in contaminated areas, can accumulate metallic trace elements (MTEs), elements of high atomic density (greater than 4 g/cm^3^ or at least 5 times the density of water), in some cases being highly toxic even at low concentrations. This group includes lead (Pb), cadmium (Cd), mercury (Hg), arsenic (As), and hexavalent chromium (Cr VI). It has been reported that metals such as copper (Cu), trivalent chromium (Cr III), iron (Fe), zinc (Zn), and nickel (Ni) are essential nutrients that are required for various biochemical and physiological functions. Inadequate supply of these micro-nutrients results in a variety of deficiency diseases or syndromes. The content of these elements in plants depends on their concentration in soil, water, and air [9]. Human industrial activity has contributed to the increased concentration of MTEs in the environment, the main threat is their bioaccumulation in the food chain. Plants absorbing toxic elements from the environment pose a potential threat to the health of animals and people who consume them [10]. Metallic trace elements accumulating in the body can damage internal organs (liver, kidneys), or contribute to disorders in the functioning of the nervous, hematopoietic (anemia), skeletal, and cardiovascular (hypertension) systems. In chronic exposure (taking small doses of toxic substances for a long period), e.g. with food, nonspecific disease symptoms on the part of the exposed organism may appear (chronic fatigue, headaches, eating disorders) [11, 12].

#### Cadmium

The International Agency for Research on Cancer (IARC) classifies cadmium in group I of compounds known to be carcinogenic and teratogenic to humans [13]. It has been confirmed that this element can also cause anemia, disorders of calcium and vitamin D metabolism (leading to osteomalacia and osteoporosis), iron, copper, and zinc deficiencies, as well as disturbances in the functioning of the sex glands and the immune system. Scientific research confirms that cadmium affects the cardiovascular system (causes peripheral arterial diseases, hypertension, and structural damage to the heart muscle), induces oxidative stress, contributes to disturbances in the functioning of the nervous system, and has hepatotoxic and nephrotoxic effects. The main sources of cadmium in food are seafood (shellfish, clams, oysters, crabs), meat (especially liver and kidneys), eggs, milk and dairy products, oilseeds, cocoa, beans, forest mushrooms, rice, wheat, green leafy vegetables, potatoes, carrots, and celery [14, 15].

#### Lead

Chronic exposure to lead manifests itself mainly in a detrimental effect on the central nervous system (neurological and mental disorders, lowered IQ, memory impairment, aggression, faster fatigue, and muscle paralysis). Lead is also hepatoxic, nephrotoxic, teratogenic, and embryotoxic. This element may cause disturbances in the work of the cardiovascular (increased blood pressure), respiratory, immune, and reproductive systems (reducing fertility, low birth weight in children, and miscarriages). Lead has also been classified as possibly carcinogenic in the human body (group 2A according to IARC). Food grown in soil near industrial plants, motorways or active landfills can contain high levels of lead. Plants that easily absorb lead are lettuce, radishes, spinach, parsley, carrots, beetroot, white cabbage, and cauliflower, as well as grain products (flour, groats, bread, pasta, cereals, rice). The source of lead in food products can also be devices used in production, dishes (ceramics, glass), and packaging (cans) [16, 17].

#### Nickel and Chrome

Nickel and chromium are elements that, in trace amounts, are needed for the proper functioning of the human body. Excessive amounts of these metals that enter the human body, e.g. in connection with occupational exposure or by eating food contaminated with these elements, may contribute to the appearance of negative health effects [18, 19].

Nickel enters the human body through the respiratory tract, with food and water, and through the skin. This element regulates hormonal activity and participates in lipid metabolism, however, excessive exposure to this element may result in genotoxic, hematotoxic, teratogenic, immunotoxic, and carcinogenic effects (IARC classified nickel as Group I – carcinogenic to humans). Nickel is also a highly allergenic metal (it causes dermatitis in allergic people) [20–22]. This element can cause cardiovascular diseases, kidney and liver damage, pulmonary fibrosis, iatrogenic poisoning, headaches, and gastrointestinal discomfort. Food products with the highest amount of nickel include hazelnuts, cocoa and dark chocolate, fruit, almonds, dates, figs, pineapple, plums, raspberries, cereals (bran, buckwheat, millet, whole grain bread, oats, brown rice, sesame, sunflower seeds), seafood (shrimps, mussels, oysters, crabs, salmon), vegetables (beans, savoy cabbage, leek, lettuce, lentils, peas, spinach, cabbage), soy and its products, peanuts [20–22].

Chromium, as trivalent (+ 3) chromium, has been reported as an essential trace element in that it has been postulated to be necessary for the efficacy of insulin in regulating the metabolism of carbohydrates, lipids, and proteins. Hexavalent (+ 6) chromium however is a toxic by-product of stainless steel and other manufacturing processes; therefore, it rarely occurs naturally. Exposure to (+ 6) chromium, may induce asthma and other signs of respiratory distress. The foods with the highest chromium content include cocoa, yeast, legumes, whole grains, meat, seafood, fish, eggs, and yellow and blue cheeses [23–25].

Nuts, due to their numerous nutritional values (high content of protein, fiber, macro- and microelements, phenolic compounds, EFAs, and vitamins), should permanently find their place in the diet of people who want to improve and maintain health and well-being. According to the latest recommendations, nuts can be introduced into the child’s diet, just like all other products, from the very beginning of expanding the diet. So they can be introduced into the diet after the child is 6 months old. There is growing evidence that early introduction of foods such as peanuts (at an appropriate age) might be beneficial in preventing food allergy [26].

Considering all the benefits of including nuts in consumers’ diets, it is important to remember the possible negative health effects (e.g. non-cancerous health effects) resulting from, among others, consuming nuts contaminated with heavy metals. Harmful health effects other than cancer can result from oral exposure to chemicals from a contaminated product (such as nuts). These effects are evaluated separately from cancer. Tools that allow estimating non-cancer health risks are the Hazard Quotient (HQ) and the Hazard Index (HI). A non-cancer hazard quotient signals whether such chronic health effects are likely from exposure to one chemical. If there are exposures to multiple chemicals, the hazard quotient for each chemical is added up to calculate a hazard index. Research conducted in recent years, determining the content of heavy metals in different types of nuts and estimating the health risk to consumers on this basis, shows that elements such as cadmium, lead, arsenic, chromium or mercury do not pose a non-cancerous health risk to the human body [27–30] However, researchers draw attention to the daily intake doses assumed in their estimates (usually 25–50 g/day), emphasizing that health risks may occur with higher consumption, which becomes real given the growing popularity of nuts in the diet. We should also take into consideration that nuts are only a small component of our diet, which together with vegetables, cereals, fish, seafood etc. supplies total dose of metals. Therefore, the total dietary intake could be associated with significant non-cancer health risks.

The study assumes that the accumulation of individual metallic trace elements will vary depending on the type of nut, which will result in different health risks for consumers. The study aimed to determine the content of metallic trace elements (Cd, Pb, Ni, and Cr) in the most commonly consumed nuts and to estimate the health risk (HQ and HI) of consumers of these food products, taking into account age and consumption habits. The presented study aims to supplement the available literature with detailed data on the safety of nut consumption in Poland, including elements determined in only a few Polish studies or those not yet defined (Cr) and estimated non-cancer health risks, assuming many consumption scenarios. At the time of writing the manuscript, the health risks of Polish consumers associated with the consumption of nuts had been presented in only one study [12], and it included metals such as cadmium and lead. The health risk for chromium and nickel had not been previously estimated for the studied group of consumers.

## Material and Methods

### Collection of Samples

The research material consisted of 69 samples of nuts available on the Polish market, which came from 13 countries. For the study, 16 samples of peanuts (from 3 countries), 15 samples of hazelnuts (from 4 countries), 15 samples of almonds (from 2 countries), 8 samples of cashews (from 3 countries), and 15 samples of walnuts (from 3 countries) were obtained. The largest number of nut samples came from Poland (20 samples; 29% of all samples) and the United States (13 samples; 19% of all samples); the US samples included only almonds (Table 1). The country of origin was obtained from the packaging (individual packaging in the case of packed samples or collective packaging in the case of samples purchased by weight).

**Table 1:** Type and country of origin of the nut samples included in the study

country of origin	number of samples				
	peanuts	hazelnuts	almonds	cashews	walnuts
Argentina	4	-	-	-	-
Azerbaijan	-	2	-	-	-
Chile	-	-	-	-	4
China	6	-	-	-	-
Georgia	-	2	-	-	-
India	-	-	-	2	-
Italy	-	-	2	-	-
Ivory Coast	-	-	-	2	-
Moldova	-	-	-	-	2
Poland	6	5	-	-	9
Turkey	-	6	-	-	-
USA	-	-	13	-	-
Vietnam	-	-	-	4	-
total	16	15	15	8	15

More than four-fifths of the nut samples (83% of the total) were originally packed in plastic packaging at the time of purchase, the remaining nuts were weighed in the store (“sold by weight”), placed in foil bags, and purchased (17%). 5 samples of hazelnuts, 5 samples of walnuts, and 2 samples of peanuts were sold by weight, the remaining nuts were purchased in their original packaging. In the case of peanuts, 75% were shelled nuts, the remaining 25% were purchased in the shell. 67% of the samples of hazelnuts and walnuts included in the analysis were purchased in shelled form, and 33% were nuts in shells. None of the samples analyzed were seasoned. Detailed information on individual samples (sample type, country of origin, package weight, product form) is included in Table A.1 (supplementary material).

### Samples Preparation and Chemical Analyses

A single sample consisted of 50 g of nuts (only the edible part of the nut) – taken randomly from a given package (packages ranging in size from 50 to 250 g) or from 250 g of the product sold by weight. Nuts purchased in the shell were peeled before further analysis. The samples were ground immediately after weighing in a vibrating mill (TESTCHEM, Poland – LMW-S model). Using an analytical balance (OHAUS Pioneer Plus analytical balance, USA – model PA214CM/1), one-gram weighted portions were prepared from the ground samples of nuts. The samples were weighed directly into 100 mL Teflon containers. 8 ml of supra pure grade reagent (Merck, Germany) of nitric acid was utilized for the digestion of nuts samples in a Teflon container using microwave reactor, model Magnum II (ERTEC, Poland) with computer control of pressure and temperature. The mineralization of the nut samples was carried out in four stages: Stage I – mineralization time 5 min, 60% power, pressure 17–20 bar; Stage II – digestion time 5 min, 80% of power, pressure 30–32 bar; Stage III – digestion time 10 min, 100% power, pressure 42–45 bar; Stage IV – sample cooling for 10 min. After mineralization, the samples were placed in 25 mL flasks.

The content of cadmium, lead, nickel, and chromium in the analyzed nut samples was determined by the method of electrothermal atomic absorption spectrometry (ETAAS) using the spectrometer model Savant AA Sigma (GBC, Australia). Recycle values were identified by using standard reference solutions. Each sample was prepared for ETAAS analysis including duplications, blank, and certified standards. The analysis of standard reference substances for nut samples allows the identification of accuracy and sensitivity in various element concentrations. In the method used, the limit of detection (LOD) was as follows: for Cd = 0.0017 mg/kg, for Pb = 0.018 mg/kg, for Ni = 0.27 mg/kg and Cr = 0.034 mg/kg. The limit of quantitation (LOQ) amounted to: for Cd = 0.0032 mg/kg, for Pb = 0.032 mg/kg, for Ni = 0.44 mg/kg, and for Cr = 0.06 mg/kg. The limits of detection (LOD) and quantification (LOQ) were determined from 20 independent blank samples, measured three times. The mean value and standard deviation were calculated for the obtained values. LOD and LOQ were calculated according to the following formulas: LOD = mean value of blank sample signals + 3*standard deviation (three times the standard deviation was added to the mean value of blank sample signals), LOQ = mean value of blank sample signals + 6*standard deviation (six times the standard deviation was added to the mean value of blank sample signals). The efficiency of element recovery was achieved during monitoring of the validity of the results. In accordance with the procedures in force in the Analytical Laboratory, the elements are recovered in the range of 80% to 120%. Elements recovered at the level of: nickel 98%, chromium 107%, cadmium 95%, and lead 101%.

### Health Risk Assessment

Based on the obtained results of laboratory analyses, the estimated exposure of nut consumers to heavy metals was made. This assessment covers non-cancerous health risks. The calculations were based on the abbreviated mathematical formula recommended by the United States Environmental Protection Agency [31]. The estimation of oral exposure consists of two steps. The first step is to calculate the average daily dose (ADD). In the second step, using the obtained ADD values, the hazard quotient (HQ) is calculated. Additionally, based on the obtained HQ values for individual metals, which cause a similar toxic effect in the exposed organism (in this work for cadmium, lead, nickel, and chromium), and for individual samples (in this case for individual nut species), the hazard index (HI) can be calculated, which was done. The formulas and individual components of the calculations are presented in Table 2.

**Table 2:** Mathematical equations and components used to calculate the average daily dose (ADD), the hazard quotient (HQ), and the hazard index (HI)

equation	ADD = C x IngR / BW	HQ = ADD / RfD	HI = HQ_1_ + HQ_2_ + … + HQ_i_
components of the equation	**ADD** **–** average daily dose [mg/kg] **C** – element concentration [mg/kg]	**HQ** – hazard quotient [unitless]	**HI** – hazard index [unitless]
	**IngR** – daily ingestion rate [kg]	**RfD** – reference dose [mg/kg]	**HQ**_**i**_ – hazard quotient for the *i*_th_ heavy metal
	**BW** – body weight [kg]		

The reference dose (RfD) for cadmium is 0.0010 mg/kg, for lead is 0.0036 mg/kg, for nickel 0.0200 mg/kg, and for chromium 0.0030 mg/kg [32–35]. If the value of HQ and HI is less than or equal to 1.0, the risk is considered negligible to low, and no unacceptable effects will occur in the exposed population of receptors. When the value is greater than 1, non-cancer health effects are possible, but not certain.

Several exposure scenarios were assumed in the study. Each scenario was estimated for 8 age groups (Table 3), taking into account the mean body weight for a given population [36]. The scenarios assume a daily nut consumption of 10 to 100 g (daily intake is described in the equation as ingestion rate (IngR)), in increments of 10 g (10 intervals in total: where scenario A means 10 g and scenario J means 100 g). For each consumption interval, the minimum (scenario I), average (scenario II), and maximum (scenario III) concentration of a given element in each of the 5 groups of nuts were taken into account.

**Table 3:** Age groups and their assigned body weight

population by age group	weight [kg]
child 6–11 months	9.20
child 1– < 2 years	11.40
child 2– < 3 years	13.80
child 3– < 6 years	18.60
child 6– < 11 years	31.80
child 11– < 16 years	56.80
child 16– < 21 years	71.60
adult	80.00

The authors included the youngest group of children (infants from 6 to 11 months of age) in the estimated exposure scenarios, but they are aware that the assumed intake is hypothetical rather than realistic.

## Results and Discussion

### Cd, Pb, Ni, and Cr Concentrations in Nuts

11 samples of walnuts, 7 samples of cashews, 6 samples of peanuts, 3 samples of almonds, and 1 sample of hazelnuts were below the LOQ for cadmium. 10 samples of peanuts, 8 samples of almonds, 7 samples of cashews, 6 samples of walnuts, and 3 samples of hazelnuts were below the LOQ for lead. In the case of nickel, 11 samples of almonds, 3 samples of peanuts and 1 sample of walnuts were below the LOQ. 2 samples of walnuts and 1 sample of hazelnuts were below the LOQ for chromium. In total, when analyzing all nuts, below the LOQ were: in the case of cadmium 41% of the samples, in the case of lead it was 49% samples, nickel 21%, and chromium 4%.

**Fig. 1:**
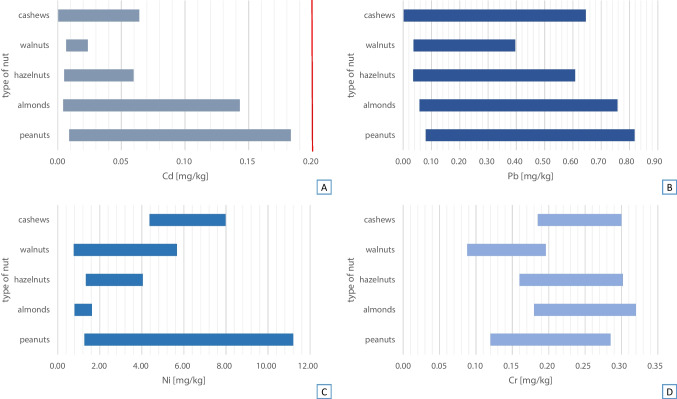
The concentration range of cadmium (**A**), lead (**B**), nickel (**C**), and chromium (**D**) in the tested samples of nuts available on the Polish market

In the EU legislation, the maximum permissible level (MPL) of the analyzed elements in tree nuts and peanuts has been established only for cadmium, which is 0.20 mg/kg [37]. No maximum levels have been established for lead, nickel, and chromium. The level of cadmium in none of the tested samples of nuts exceeded the MPL assigned for this element (Fig. 1A). The highest mean concentration of cadmium was recorded in peanuts (0.092 mg/kg), and the lowest in cashews (in 7 out of 8 samples the concentration of Cd was below the LOQ). The concentration of Cd in all analyzed samples of nuts was as follows: peanuts > almonds > hazelnuts > walnuts > cashews (Fig. 1A). In 2023, updated Chinese maximum levels for contaminants in food were released, setting the MPL for cadmium at a higher level than in the European Union, at 0.5 mg/kg [38]. Therefore, none of the obtained Cd concentrations in the analyzed samples exceeded the standards applicable in China.

In the case of lead, the highest mean concentration of this element was recorded (in the same way as in the case of cadmium) in peanuts (0.229 mg/kg), and the lowest in cashews (in 7 out of 8 samples the concentration of Pb was below the LOQ). The concentration of Pb was as follows: peanuts > almonds > hazelnuts > walnuts > cashews (Fig. 1B). However, the highest mean nickel concentration was recorded in cashews (6.434 mg/kg), while the lowest was in almonds (1.299 mg/kg). The Ni concentrations were as follows: cashews > peanuts > walnuts > hazelnuts > almonds (Fig. 1C). The highest mean concentration of chromium was recorded in almonds (0.253 mg/kg), and the lowest in walnuts (0.133 mg/kg). The Cr concentrations were as follows: almonds > cashews and hazelnuts > peanuts > walnuts (Fig. 1D). In addition to the concentration of cadmium in nuts, Chinese standards also specify an MPL for lead, but this applies only to peanuts and is 0.2 mg/kg [38]. This value was exceeded in one sample where the Pb concentration was determined to be 0.818 mg/kg (country of origin of peanuts: Poland).

Taking into account the type of nuts and the country of origin, the average concentrations of the elements were as follows: a) for peanuts – Cd: China > Poland > Argentina, Pb: Poland > China > Argentina, Ni & Cr: China > Argentina > Poland; b) for almonds – Cd, Pb & Ni: Italy > USA, Cr: USA > Italy; c) for hazelnuts – Cd: Turkey > Poland > Azerbaijan > Georgia, Pb: Turkey > Poland > Georgia > Azerbaijan, Ni: Poland > Turkey > Georgia > Azerbaijan, Cr: Azerbaijan > Poland > Georgia > Turkey; d) for walnuts – Cd & Ni: Moldova > Poland > Chile, Pb: Moldova > Chile > Poland, Cr: Poland > Moldova > Chile; e) for cashews – Cd & Pb: values above LOQ were only recorded in the samples from India, Ni: India > Vietnam > Ivory Coast, Cr: Ivory Coast > Vietnam > India.

The consumption of nuts has several positive health consequences. However, the question should be asked whether it is always safe or whether it may pose a risk to health. A potential health risk may result from the consumption of nuts contaminated with metallic trace elements (MTEs), like cadmium or lead. Based on the presented results, to reduce the health risk, one should pay attention to the origin of the nuts, their type, and the amount of consumption, adjusted to the consumer’s age. Few studies are taking into account the pollution of nuts with MTEs, and they mainly concern China and Turkey [27, 39–44]. Even fewer studies estimate the acceptable daily intake of nuts that will be safe for consumers [12, 28, 43] concerning exposure to MTEs. Single studies were conducted in Slovenia, Spain, Korea, Poland, Slovakia, Sweden, Brazil, Bosnia and Herzegovina, Serbia, Belgium, and Luxembourg [20–22, 45–52].

Comparing the results obtained in the publications mentioned above, the highest concentration of cadmium was determined in hazelnuts (6.95 mg/kg) in the studies conducted in Bosnia and Herzegovina by *Bašić *et al*.* [51]. Cadmium concentrations below the detection limit (< LOD) have been documented in the studies carried on by *Cabrera *et al*.* [45] – in the case of all tested nut and peanut samples, *Harangozo *et al*.* [48] and *Kalkıșım *et al*.* [40] – in case of walnuts, *Muller *et al*.* [50] – in almond and cashew samples, as well as in the study by *Tošić *et al*.* [52] – in cashew, hazelnut and walnut samples. In the own study, cadmium concentrations ranged from < LOQ (for all tested nuts) to 0.183 mg/kg in peanuts, with the mean concentration for all tested samples equal to 0.030 mg/kg. The obtained single results were comparable to the cadmium concentrations determined in the cited studies. When analyzing the lead content, the highest concentration of this element was recorded in peanuts (18.64 mg/kg), as well as in cashews (6.61 mg/kg), walnuts (3.43 mg/kg), and almonds (2.81 mg/kg). All samples were obtained in Korea by *Chung *et al*.* [46] and were significantly higher than the results obtained by other researchers. Concentrations below the LOD value were obtained in studies conducted by: *Muller *et al*.* [49] – in the case of hazelnut, almond, walnut, and cashew samples, *Yin *et al*.* [41] – in the case of almonds, walnuts, and cashews, *Harangozo *et al*.* [48] and *Taghizadeh *et al*.* [28] – in case of walnuts. This study showed incomparably lower concentrations of lead than in the Korean research, and at the same time comparable to the works of other researchers. The concentration of Pb in the tested nuts ranged from < LOQ (for all tested types of nuts) to 0.82 mg/kg in the case of peanuts, with the mean concentration for all tested samples equal to 0.143 mg/kg. In studies conducted by *Rodushkin *et al*.* [49], when investigating levels of inorganic constituents in raw nuts and seeds on the Swedish market, the lowest chromium concentrations were found – in walnuts (0.0013 mg/kg), in hazelnuts (0.0044 mg/kg), in almonds (0.0056 mg/kg) and cashew nuts (0.0120 mg/kg). The highest concentrations of Cr were obtained in the studies conducted by *Tošić *et al*.* [52], where the concentrations of chromium in nuts from the Serbian market were: 50.53 mg/kg in hazelnuts, 41.77 mg/kg in peanuts, 40.65 mg/kg in almonds and 17.40 mg/kg in walnuts. Analyzing own results, the lowest chromium concentrations (< LOQ) were found in walnuts and hazelnuts, and the highest in almonds (0.320 mg/kg); the mean value for all nut samples was 0.211 mg/kg. The results obtained were therefore much lower than those carried out in Serbia [52]. The highest nickel concentration was obtained in cashews purchased in Serbia [52], Luxembourg [22], and Sweden [49], and it was 7.75 mg/kg, 6.80 mg/kg, and 6.70 mg/kg, respectively. The lowest concentrations of Ni have been documented in nuts available on the Spanish market [45] – 0.02 mg/kg for peanuts, and 0.10 mg/kg for other nuts.

The highest nickel concentration obtained in own research was recorded for the peanut sample (11,20 mg/kg), and compared to other studies it was the highest concentration. Below the LOQ were samples of almonds, but also of peanuts. The mean nickel concentration for all tested samples was 3.21 mg/kg. Summarizing own research, the concentrations of elements in all tested types of nuts, in most cases, were arranged as the following diminishing series: Ni > Cr > Pb > Cd.

Comparing the results obtained only for nuts available on the Polish market, the study conducted by *Bielecka *et al*.* [12] determined, among others: the concentration of cadmium and lead in peanuts, walnuts, hazelnuts, almonds, and cashews. The highest mean Cd concentration (0.70 mg/kg) was determined for hazelnuts and the lowest (< 0.002 mg/kg) for both almonds and walnuts. In own study, the highest mean Cd concentration was determined for peanuts (0,09 mg/kg) and the lowest for cashews (< LOQ). Comparing only nuts included in own study, in the *Bielecka *et al*.* [12] study, the analysis of the Pb concentration showed that the highest mean value (0.19 mg/kg) was reached by peanuts, while the lowest mean (0.01 mg/kg) concentration of Pb was in walnuts. In own study, the highest average Pb value was also determined in peanuts, and it was slightly higher, although comparable (0.23 mg/kg); a comparable level was also determined in almonds (0.22 mg/kg). In turn, the lowest average Pb concentration was determined, as in the case of cadmium, in cashews (< LOQ). In the studies conducted by Woźniak, Waśkiewicz, and Ratajczak [53], nickel concentration was determined in peanuts, walnuts, hazelnuts, almonds, and cashews, among others. The mean Ni concentration obtained in the analyzed nut samples decreased in the following order: cashews (17.98 mg/kg) > hazelnuts (14.40 mg/kg) > almonds (12.57 mg/kg) > walnuts (11.76 mg/kg) > peanuts (8.80 mg/kg). In own study, the highest concentration of nickel was also recorded in cashews (7.32 mg/kg), however, the obtained result was almost two and a half times smaller than that obtained in the work of Woźniak, Waśkiewicz, and Ratajczak [53]. In turn, the lowest concentration of Ni was recorded in almonds (1.19 mg/kg). No studies were found that determined the concentration of chromium in nuts available on the Polish market. Table B.1 (supplementary material) compares results of this study with the results of other authors.

### Hazard Quotient

When analyzing the hazard quotient values obtained in the case of peanut consumption, the situation was as follows (Supplementary material Table C.1):

- **HQ**_**Cd**_** > 1**: in the group of children from 6 to 11 months of age in 5 consumption scenarios (from 60 to 100 g/day), in the group of children aged 1– < 2 years in 4 consumption scenarios (from 70 to 100 g/day), in the group of children 2 – < 3 years in 3 consumption scenarios (80 to 100 g/day), in group 3 – < 6 years in 1 consumption scenario (100 g/day) –;
- **HQ**_**Pb**_** > 1**: in the group of children from 6 to 11 months of age in 6 consumption scenarios (from 50 to 100 g/day), in the group of children aged 1– < 2 years in 5 consumption scenarios (from 60 to 100 g/day), in the group of children 2– < 3 years in 4 consumption scenarios (70 to 100 g/day);
- **HQ**_**Ni**_** > 1**: in the group of children from 6 to 11 months of age in 9 consumption scenarios (from 20 to 100 g/day), in the group of children aged 1– < 2 years and 2– < 3 years in 8 consumption scenarios (from 30 to 100 g/day), in the group of children 3– < 6 years old in 7 consumption scenarios (from 40 to 100 g/day), in the group of children 6– < 11 years old in 5 consumption scenarios (from 60 to 100 g/day);
- **HQ**_**Cr**_** > 1**: in the group of children from 6 to 11 months of age in one consumption scenario (100 g/day).

All exceedances of the value equal to 1 were obtained for the scenarios taking into account the maximum concentration of the element (scenario III) obtained in the tested nut types – in peanuts. The exception was the consumption of 100 g of peanuts by the youngest study group – HQ > 1 was also recorded for the scenario taking into account the average cadmium concentration in peanuts.

The hazard quotient values obtained in the case of almond consumption were as follows (Supplementary material Table C.2):

- **HQ**_**Cd**_** > 1**: in the group of children from 6 to 11 months of age in 4 consumption scenarios (from 70 to 100 g/day), in the group of children aged 1– < 2 years in 3 consumption scenarios (from 80 to 100 g/day), in the group of children 2– < 3 years in 1 consumption scenario (100 g/day);
- **HQ**_**Pb**_** > 1**: in the group of children from 6 to 11 months of age in 6 consumption scenarios (from 50 to 100 g/day), in the group of children aged 1– < 2 years in 5 consumption scenarios (from 60 to 100 g/day), in the group of children 2– < 3 years old in 4 consumption scenarios (70 to 100 g/day), children 3– < 6 years old in 2 consumption scenarios (90 to 100 g/day);
- **HQ**_**Ni**_** > 1**: not recorded;
- **HQ**_**Cr**_** > 1**: in the group of children from 6 to 11 months of age in 2 consumption scenarios (from 90 to 100 g/day).

All exceedances of the value equal to 1 were obtained for the scenarios considering the maximum concentration of the element (scenario III) obtained in the tested nut types – in almonds.

In the case of hazelnuts consumption, the obtained hazard quotient values were as follows (Supplementary material Table C.3):

- **HQ**_**Cd**_** > 1**: not recorded;
- **HQ**_**Pb**_** > 1**: in the group of children from 6 to 11 months of age in 5 consumption scenarios (from 60 to 100 g/day), in the group of children aged 1– < 2 years in 4 consumption scenarios (from 70 to 100 g/day), in the group of children 2– < 3 years in 2 consumption scenarios (90 to 100 g/day);
- **HQ**_**Ni**_** > 1**: in the group of children from 6 to 11 months of age in 6 consumption scenarios (from 50 to 100 g/day), in the group of children aged 1– < 2 years in 5 consumption scenarios (from 60 to 100 g/day), in the group of children 2– < 3 years in 4 consumption scenarios (from 70 to 100 g/day), in the group of children 3– < 6 years in 1 consumption scenario (100 g/day);
- **HQ**_**Cr**_** > 1**: in the group of children from 6 to 11 months of age in one consumption scenario (100 g/day).

All exceedances of the value equal to 1 were obtained for the scenarios taking into account the maximum concentration of the element (scenario III) obtained in the tested nut types – in hazelnuts.

Hazard quotient values obtained in the case of consumption of walnuts were as follows (Supplementary material Table C.4):

- **HQ**_**Cd**_** > 1**: not recorded;
- **HQ**_**Pb**_** > 1**: in the group of children from 6 to 11 months of age in 2 consumption scenarios (from 90 to 100 g/day);
- **HQ**_**Ni**_** > 1**: in the group of children from 6 to 11 months of age in 7 consumption scenarios (from 40 to 100 g/day), in the group of children aged 1– < 2 years and 2– < 3 years in 6 consumption scenarios (from 50 to 100 g/day) day), in the group of children 3– < 6 years old in 4 consumption scenarios (from 70 to 100 g/day);
- **HQ**_**Cr**_** > 1**: not recorded.

Again, all exceedances of the value equal to 1 were obtained for the scenarios taking into account the maximum concentration of the element (scenario III) obtained in the tested nut species – in walnuts.

In the case of the consumption of the last tested group of products – cashew nuts, the obtained hazard quotient values were as follows (Supplementary material Table C.5):

- **HQ**_**Cd**_** > 1**: not recorded;
- **HQ**_**Pb**_** > 1**: in the group of children from 6 to 11 months of age in 5 consumption scenarios (from 60 to 100 g/day), in the group of children aged 1– < 2 years in 4 consumption scenarios (from 70 to 100 g/day), in the group of children 2– < 3 years in 3 consumption scenarios (80 to 100 g/day);
- **HQ**_**Ni**_** > 1**: in the group of children from 6 to 11 months of age and 1– < 2 years in 8 consumption scenarios (from 30 to 100 g/day), in the group of children aged 2– < 3 years in 7 consumption scenarios (from 40 to 100 g/day), in the group of children 3– < 6 years in 6 consumption scenarios (50 to 100 g/day), in the group of children 6– < 11 years in 3 consumption scenarios (80 to 100 g/day),
- **HQ**_**Cr**_** > 1**: in the group of children from 6 to 11 months of age in one consumption scenario (100 g/day).

All exceedances of the value equal to 1 were obtained for the scenarios taking into account the maximum concentration of the element (scenario III) obtained in the tested types of nuts – cashews.

*Bielecka *et al*.* [12] assessed the health risk resulting from the consumption of contaminated nuts using indicators such as the target hazard quotient (THQ), cancer risk (CR), and hazard index (HI). The average daily consumption in the quoted study was estimated at 42 g, the average body weight was 70 kg, and the health risk indicators did not identify increased health risk in the case of cadmium and lead. *Taghizadeh *et al*.* [28] measured heavy metal levels (i.a. Pb, Cr) in six walnut cultivars from five geographical zones of Iran. They also calculated the target hazard quotient (THQ), and additionally the incremental lifetime cancer risk (ILCR). Based on the calculated 95% ILCR, dietary exposure to Pb and Cr through the consumption of walnuts does not pose a risk to Iranian consumer health. *Gu *et al*.* [43], calculated chronic daily intake (CDI) and hazard quotient (HQ) and indicated that Cd and Pb exhibited a slight health risk to adult residents of China.

### Hazard Index

When analyzing the Hazard Index, taking into account the health risk resulting from oral exposure to individual MTEs contained in the tested nuts, and their simultaneous consumption during the day, the value of HI > 1 was recorded in the case of (Table 4):

- **HI**_**Cd**_** > 1**: in the group of children from 6 to 11 months of age in scenarios B–J (maximum concentration of the element; scenario III) and in scenarios G–J (average concentration of the element; scenario II), in the group of children aged 1– < 2 years in scenarios C–J (scenario III) and in scenarios H–J (scenario II), in the group of children aged 2– < 3 years in scenarios C–J (scenario III) and in scenario J (scenario II), in the group of children aged 3– < 6 years in scenarios D–J (scenario III), in the group of children aged 6– < 11 years in scenarios G–J (scenario III);
- **HI**_**Pb**_** > 1**: in the group of children from 6 to 11 months of age in scenarios B–J (scenario III) and in scenarios E–J (scenario II), in the group of children aged 1– < 2 years in scenarios B–J (scenario III) and in scenarios F–J (scenario II), in the group of children aged 2– < 3 years in scenarios B–J (scenario III) and in scenarios G–J (scenario II), in the group of children aged 3– < 6 years in scenarios C–J (scenario III) and scenario J (scenario II), in the group of children aged 6– < 11 years in scenarios D–J (scenario III), in the group of children aged 11– < 16 years and 16– < 21 years old in scenarios G–J (scenario III), in the group of adults in scenarios I–J (scenario III);
- **HI**_**Ni**_** > 1**: in the group of children from 6 to 11 months of age and aged 1– < 2 years in scenarios A–J (III), in scenarios B–J (II) and C–J (minimum element concentration; scenario I), in the group of children at the age of 2– < 3 in scenarios A–J (III), in scenarios B–J (II) and D–J (I), in the group of children aged 3– < 6 in scenarios B–J (III), in scenarios C–J (II) and E–J (I), in a group of children aged 6– < 11 years in scenarios C–J (III), D–J (II) and H–J (I), in the group of children aged 11– < 16 in scenarios D–J (III) and H–J (II), in the group of children aged 16– < 21 in scenarios E–J (III) and I–J (II), as well as in the group of adults in scenarios F–J (III) and J (II);
- **HI**_**Cr**_** > 1**: in the group of children from 6 to 11 months of age in scenarios B–J (III), C–J (II), and D–J (I), in the group of children aged 1– < 2 years in scenarios C–J (III), D–J (II) and E–J (I), in the group of children aged 2– < 3 years in scenarios C–J (III), D–J (II) and F–J (I), in the group children aged 3– < 6 years in scenarios D–J (III), F–J (II) and H–J (I), in the group of children aged 6– < 11 years in scenarios G–J (III) and J (II).

**Table 4:**
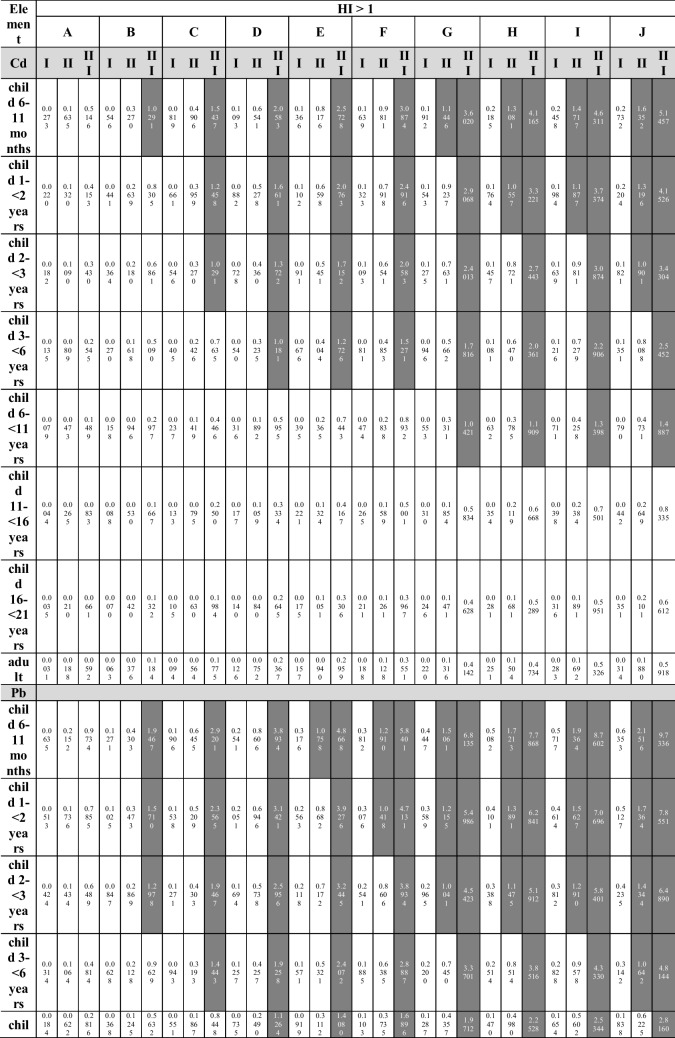

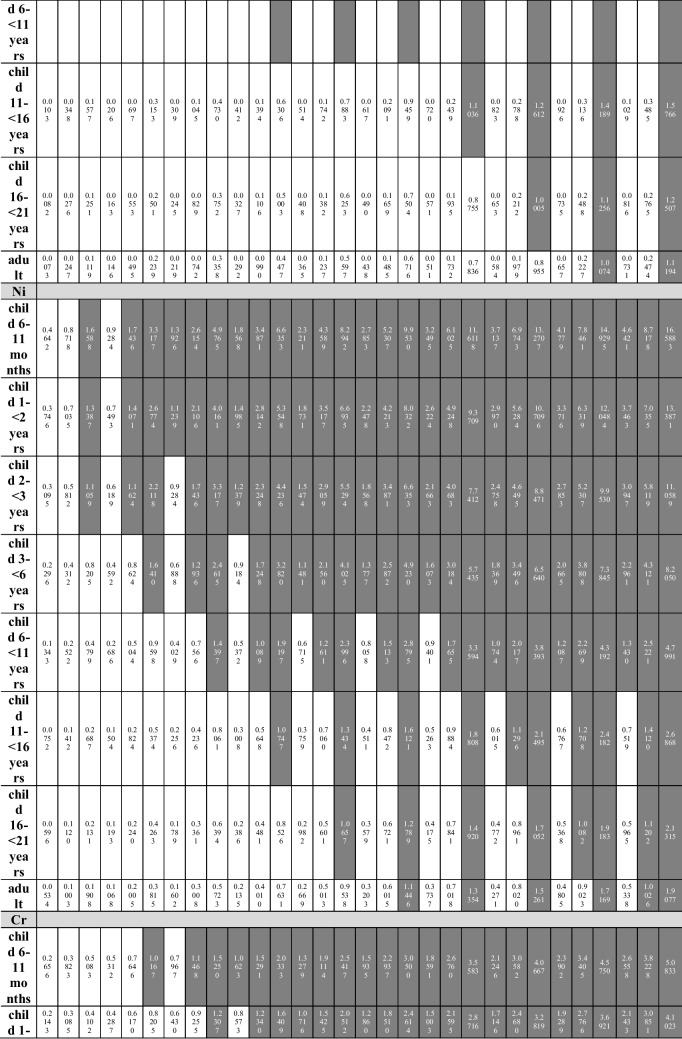

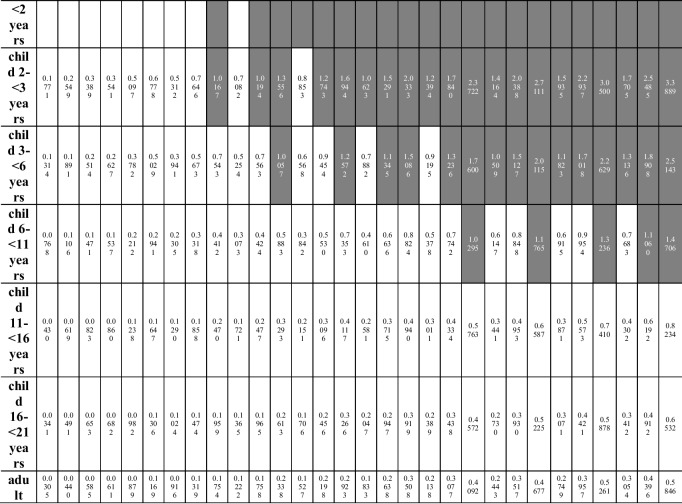
Hazard Index (HI) calculated for Cd, Pb, Ni, and Cr taking into account the simultaneous consumption of all tested nut types

Taking into account the simultaneous consumption, it should be remembered that the consumption scenarios will increase as follows: scenario A assumes the consumption of not 10 but 50 g of nuts, and scenario J, in turn, not 100 g but half a kilogram (500 g), which for the majority of the analyzed groups of consumers is unlikely to happen.

The consumption scenarios remain unchanged in the case of the analysis of the hazard index value calculated for Cd, Pb, Ni, and Cr, taking into account the exposure to all analyzed elements marked in one type of nut. HI values above 1 were noted in the case of consumption (Table 5):

- **Peanuts**: in the group of children from 6 to 11 months of age in all 10 consumption scenarios (from 10 to 100 g/day) taking into account the maximum element concentration (scenario III), in 8 consumption scenarios (from 30 to 100 g/day) taking into account the average element concentration (scenario II) and in 4 consumption scenarios (from 70 to 100 g/day) taking into account the minimum concentration of elements (scenario I); in the group of children aged 1– < 2 years in 9 consumption scenarios (from 20 to 100 g/day) taking into account the maximum element concentration (III), in 8 consumption scenarios (from 30 to 100 g/day) taking into account the average element concentration (II) and in 2 consumption scenarios (from 90 to 100 g/day) taking into account the minimum concentration of the elements (I); in the group of children aged 2– < 3 years in 9 consumption scenarios (20–100 g/day) in scenario III and in 7 (40–100 g/day) in scenario II; in the group of children aged 3– < 6 years in 9 consumption scenarios (20–100 g/day) in scenario III and in 6 (50–100 g/day) in scenario II; in the group of children aged 6– < 11 years old in 8 consumption scenarios (30–100 g/day) in scenario III and in 2 (90–100 g/day) in scenario II; in the group of children aged 11– < 16 years in 5 consumption scenarios (60–100 g/day) in scenario III; in the group of children aged 16– < 21 years in 4 consumption scenarios (70–100 g/day) in scenario III; in the group of adults in 3 consumption scenarios (80–100 g/day) in scenario III;
- **Almonds**: in the group of children from 6 to 11 months of age in 9 consumption scenarios (from 20 to 100 g/day) taking into account the maximum element concentration (III), in 7 consumption scenarios (from 40 to 100 g/day) taking into account the average element concentration (II) and in 3 consumption scenarios (from 80 to 100 g/day) taking into account the minimum concentration of the elements (I); in the group of children aged 1– < 2 years in 8 consumption scenarios (from 30 to 100 g/day) taking into account the maximum element concentration (III), in 6 consumption scenarios (from 50 to 100 g/day) taking into account the average element concentration (II) and in 1 consumption scenario (100 g/day) taking into account the minimum element concentration (I); in the group of children aged 2– < 3 years in 8 consumption scenarios (30–100 g/day) in scenario III and in 5 (60–100 g/day) in scenario II; in the group of children aged 3– < 6 years in 7 consumption scenarios (40–100 g/day) in scenario III and in 3 (80–100 g/day) in scenario II; in the group of children aged 6– < 11 years in 5 consumption scenarios (60–100 g/day) in scenario III;
- **Cashews**: in the group of children from 6 to 11 months of age in 9 consumption scenarios (from 20 to 100 g/day) taking into account scenario III, in 8 consumption scenarios (from 30 to 100 g/day) taking into account scenario II and in 7 consumption scenarios (from 40 up to 100 g/day) taking into account the minimum concentration of elements (scenario I); in the group of children aged 1– < 2 years in 9 consumption scenarios (from 20 to 100 g/day) taking into account scenario III, in 8 consumption scenarios (from 30 to 100 g/day) taking into account scenario II and in 6 consumption scenarios (from 50 to 100 g/day) taking into account scenario I; in the group of children aged 2– < 3 years in 9 consumption scenarios (20–100 g/day) in scenario III, in 7 (40–100 g/day) in scenario II and in 6 (50–100 g/day) in scenario I; in the group of children aged 3– < 6 years in 8 consumption scenarios (30–100 g/day) in scenario III, in 6 (50–100 g/day) in scenario II and in 4 (70–100 g/day) in scenario I; in the group of children aged 6– < 11 years in 6 consumption scenarios (50–100 g/day) in scenario III and in 3 (80–100 g/day) in scenario II; in the group of children aged 11– < 16 years in 3 consumption scenarios (80–100 g/day) in scenario III; in the group of children aged 16– < 21 years in the consumption scenario 1 (100 g/day) for scenario III;
- **Hazelnuts**: in the group of children from 6 to 11 months of age in 9 consumption scenarios (from 20 to 100 g/day) taking into account scenario III, in 7 consumption scenarios (from 40 to 100 g/day) taking into account scenario II and in 4 consumption scenarios (from 70 up to 100 g/day) taking into account the minimum scenario I; in the group of children aged 1– < 2 years in 8 consumption scenarios (from 30 to 100 g/day) taking into account scenario III, in 6 consumption scenarios (from 50 to 100 g/day) taking into account scenario II and in 2 consumption scenarios (from 90 to 100 g/day) taking into account scenario I; in the group of children aged 2– < 3 years in 8 consumption scenarios (30–100 g/day) in scenario III, in 5 (60–100 g/day) in scenario II; in the group of children aged 3– < 6 years in 7 consumption scenarios (40–100 g/day) in scenario III and in 3 (80–100 g/day) in scenario II; in the group of children aged 6– < 11 years in 5 consumption scenarios (60–100 g/day) in scenario III;
- **Walnuts**: in the group of children from 6 to 11 months of age in 9 consumption scenarios (from 20 to 100 g/day) taking into account scenario III, in 7 consumption scenarios (from 40 to 100 g/day) taking into account scenario II; in the group of children aged 1– < 2 years in 8 consumption scenarios (from 30 to 100 g/day) taking into account scenario III and in 6 consumption scenarios (from 50 to 100 g/day) taking into account scenario II; in the group of children aged 2– < 3 years in 8 consumption scenarios (30–100 g/day) in scenario III, in 5 (60–100 g/day) in scenario II; in the group of children aged 3– < 6 years in 7 consumption scenarios (40–100 g/day) in scenario III and in 2 (90–100 g/day) in scenario II; in the group of children aged 6– < 11 years in 4 consumption scenarios (70–100 g/day) in scenario III.

**Table 5:**
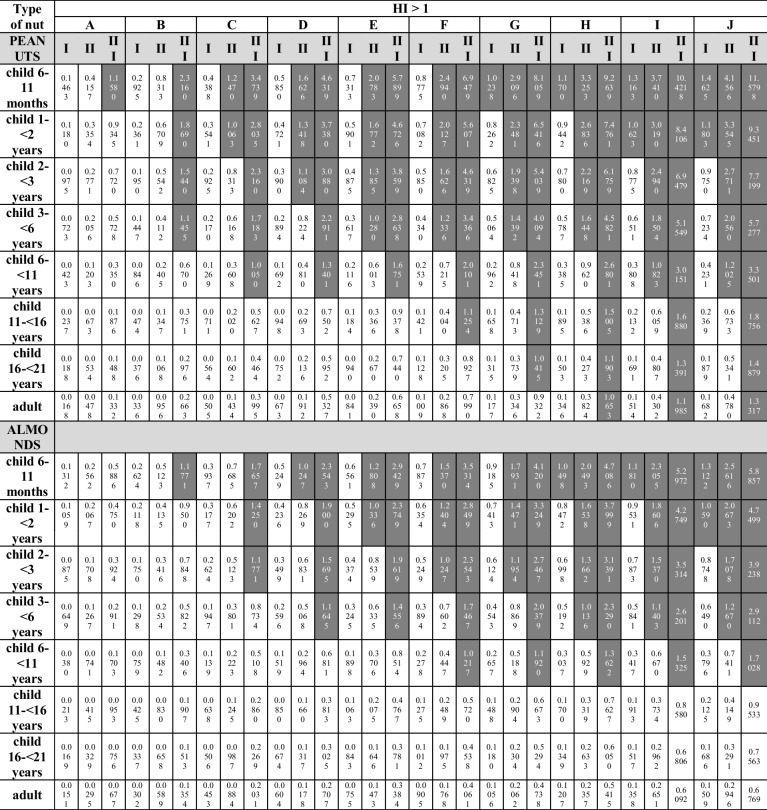

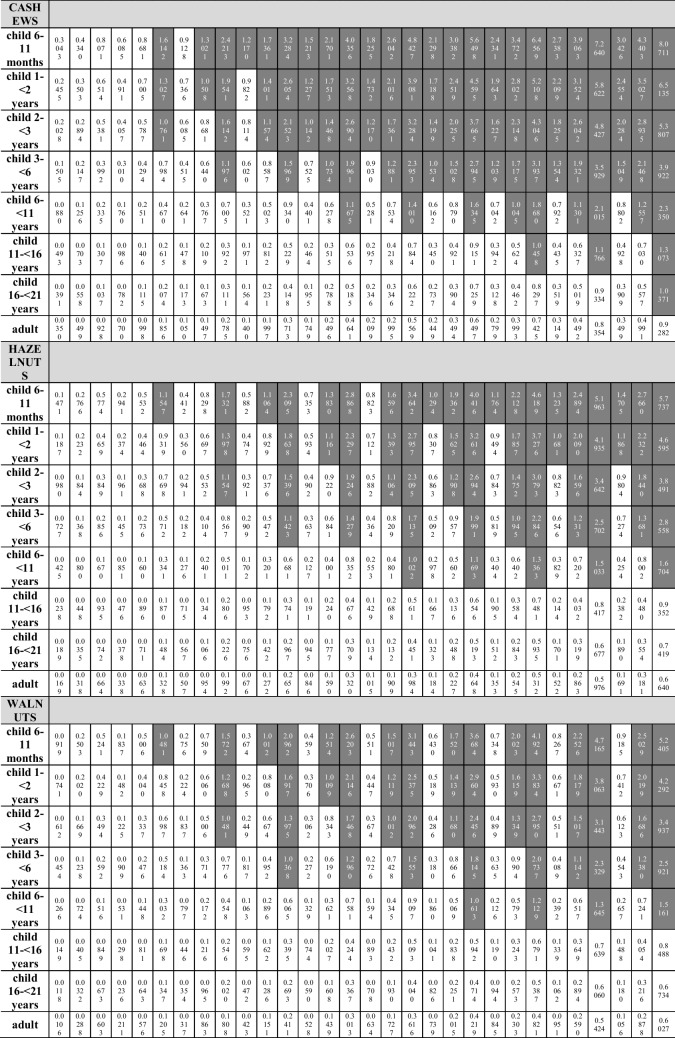
Hazard Index (HI) calculated for Cd, Pb, Ni, and Cr taking into account exposure to all elements present in one type of nut

Additionally, HI was estimated assuming simultaneous consumption of nuts in different configurations (e.g. walnuts—hazelnuts, almonds—peanuts—cashews, hazelnuts—walnuts—peanuts—almonds, etc.). The obtained results are presented in Tables D.1–D.3 (supplementary material).

The Hazard Index (for non-carcinogenic risk scenarios) was estimated in studies conducted by *Taghizadeh *et al*.* [28]. A Hazard Index less than or equal to 1 was found for simultaneous exposure to Cr and Pb, which suggests an acceptable level of non-carcinogenic adverse health risk. The Hazard Index calculated in the studies by *Gu *et al*.* [43] also did not exceed the value equal to one.

## Conclusions

The growing consumption of nuts in recent years, not only because of their taste but mainly based on their nutritional properties and beneficial effects, requires periodical control. The own study showed the presence of MTEs in nuts such as almonds, cashews, hazelnuts, peanuts, and walnuts. The obtained results of element concentrations were, with minor exceptions, comparable to those obtained in studies by other Authors. Apart from cadmium, for which a European standard has been established, it is difficult to determine whether a given concentration of elements (Pb, Ni, Cr) in edible nuts is or is not safe for consumers. An effective tool that allows for an approximate estimation of health risk is the Hazard Quotient and Hazard Index. The study takes into account the exposure of the entire population (children and adults), for which many exposure scenarios have been assumed. In many scenarios, the HQ and HI values ​​exceeded 1, which can be translated into the possibility of non-cancer health risks in a given population. We are aware that the scenarios with the highest consumption in the toddler population in most cases do not reflect actual consumption. However, assessing such risk gives an overall view of the situation. The results will allow to plan the diet of children and adults which includes nuts. It should be remembered that nuts in our diet are usually not only in their natural form – we also have them in plant milk (e.g. almond milk), butter (e.g. peanut or almond butter), bars, etc. In summary, the present findings, based on health risk indicators, are of potential use to food composition tables. Additionally, the presented results may, and even should, serve as a starting material for further research in this area, which will supplement the obtained results with other types of nuts (such as macadamia nuts, pistachios, Brazil nuts, and pecans).

## Supplementary Information

Below is the link to the electronic supplementary material.

Supplementary file1 (DOCX 262 KB)

## Data Availability

No datasets were generated or analysed during the current study.
