# Supplementary material for: Evaluation of Cadmium, Lead, Chromium, and Nickel Content in Various Types of Nuts: Almonds, Cashews, Hazelnuts, Peanuts, and Walnuts – Health Risk of Polish Consumers
Source: Biol Trace Elem Res. 2024 Nov 6;203(7):3913–30. doi: 10.1007/s12011-024-04438-4 (PMC12174183; doi:10.1007/s12011-024-04438-4)
Supplement: Supplementary file 1 — Supplementary file1 (DOCX 262 KB) [file 12011_2024_4438_MOESM1_ESM.docx]

**Table A.1** Sample characteristics

| **No.** | **Sample code** | **Sample type** | **Packaging** | **Country of origin** | **Sample form** | **Package weight [g]** | |
| --- | --- | --- | --- | --- | --- | --- | --- |
| 1 | K79 /II/20/21 | almonds | plastic packaging | USA | shelled, unroasted, unsalted | | 100 |
| 2 | K80 /II/20/21 |  | plastic packaging | USA | shelled, unroasted, unsalted | | 100 |
| 3 | K83 /II/20/21 |  | plastic packaging | USA | shelled, unroasted, unsalted | | 100 |
| 4 | K109 /II/20/21 |  | plastic packaging | USA | blanched, unsalted | | 100 |
| 5 | K110 /II/20/21 |  | plastic packaging | USA | blanched, unsalted | | 100 |
| 6 | K111 /II/20/21 |  | plastic packaging | USA | shelled, unroasted, unsalted | | 100 |
| 7 | K112 /II/20/21 |  | plastic packaging | USA | shelled, unroasted, unsalted | | 100 |
| 8 | K113 /II/20/21 |  | plastic packaging | USA | shelled, unroasted, unsalted | | 100 |
| 9 | K114 /II/20/21 |  | plastic packaging | USA | shelled, unroasted, unsalted | | 100 |
| 10 | K115 /II/20/21 |  | plastic packaging | USA | shelled, unroasted, unsalted | | 100 |
| 11 | K116 /II/20/21 |  | plastic packaging | USA | shelled, unroasted, unsalted | | 100 |
| 12 | K117 /II/20/21 |  | plastic packaging | Italy | shelled, unroasted, unsalted | | 100 |
| 13 | K118 /II/20/21 |  | plastic packaging | Italy | shelled, unroasted, unsalted | | 100 |
| 14 | K119 /II/20/21 |  | plastic packaging | USA | shelled, unroasted, unsalted | | 100 |
| 15 | K120 /II/20/21 |  | plastic packaging | USA | shelled, unroasted, unsalted | | 100 |
| 16 | K121 /II/20/21 | cashews | plastic packaging | India | shelled, unroasted, unsalted | | 50 |
| 17 | K122 /II/20/21 |  | plastic packaging | India | shelled, unroasted, unsalted | | 50 |
| 18 | K123 /II/20/21 |  | plastic packaging | Vietnam | shelled, unroasted, unsalted | | 100 |
| 19 | K124 /II/20/21 |  | plastic packaging | Vietnam | shelled, unroasted, unsalted | | 100 |
| 20 | K125 /II/20/21 |  | plastic packaging | Ivory Coast | shelled, unroasted, unsalted | | 100 |
| 21 | K126 /II/20/21 |  | plastic packaging | Ivory Coast | shelled, unroasted, unsalted | | 100 |
| 22 | K127 /II/20/21 |  | plastic packaging | Vietnam | shelled, unroasted, unsalted | | 100 |
| 23 | K128 /II/20/21 |  | plastic packaging | Vietnam | shelled, unroasted, unsalted | | 100 |
| 24 | K75 /II/20/21 | hazelnuts | sold by weight | Poland | whole (in shell) | | 306 |
| 25 | K76 /II/20/21 |  | sold by weight | Poland | whole (in shell) | | 312 |
| 26 | K81 /II/20/21 |  | sold by weight | Poland | whole (in shell) | | 298 |
| 27 | K97 /II/20/21 |  | plastic packaging | Turkey | shelled, unroasted, unsalted | | 100 |
| 28 | K98 /II/20/21 |  | plastic packaging | Turkey | shelled, unroasted, unsalted | | 100 |
| 29 | K99 /II/20/21 |  | plastic packaging | Turkey | shelled, unroasted, unsalted | | 100 |
| 30 | K100 /II/20/21 |  | plastic packaging | Turkey | shelled, unroasted, unsalted | | 100 |
| 31 | K101 /II/20/21 |  | plastic packaging | Georgia | shelled, unroasted, unsalted | | 100 |
| 32 | K102 /II/20/21 |  | plastic packaging | Georgia | shelled, unroasted, unsalted | | 100 |
| 33 | K103 /II/20/21 |  | plastic packaging | Turkey | shelled, unroasted, unsalted | | 100 |
| 34 | K104 /II/20/21 |  | plastic packaging | Turkey | shelled, unroasted, unsalted | | 100 |
| 35 | K105 /II/20/21 |  | plastic packaging | Azerbaijan | shelled, unroasted, unsalted | | 100 |
| 36 | K106 /II/20/21 |  | plastic packaging | Azerbaijan | shelled, unroasted, unsalted | | 100 |
| 37 | K107 /II/20/21 |  | sold by weight | Poland | whole (in shell) | | 302 |
| 38 | K108 /II/20/21 |  | sold by weight | Poland | whole (in shell) | | 306 |
| 39 | K77 /II/20/21 | walnuts | sold by weight | Poland | whole (in shell) | | 309 |
| 40 | K78 /II/20/21 |  | sold by weight | Poland | whole (in shell) | | 299 |
| 41 | K82 /II/20/21 |  | sold by weight | Poland | whole (in shell) | | 306 |
| 42 | K85 /II/20/21 |  | plastic packaging | Poland | shelled, unroasted, unsalted | | 100 |
| 43 | K86 /II/20/21 |  | plastic packaging | Poland | shelled, unroasted, unsalted | | 100 |
| 44 | K87 /II/20/21 |  | plastic packaging | Poland | shelled, unroasted, unsalted | | 100 |
| 45 | K88 /II/20/21 |  | plastic packaging | Poland | shelled, unroasted, unsalted | | 100 |
| 46 | K89 /II/20/21 |  | plastic packaging | Chile | shelled, unroasted, unsalted | | 100 |
| 47 | K90 /II/20/21 |  | plastic packaging | Chile | shelled, unroasted, unsalted | | 100 |
| 48 | K91 /II/20/21 |  | plastic packaging | Moldova | shelled, unroasted, unsalted | | 100 |
| 49 | K92 /II/20/21 |  | plastic packaging | Moldova | shelled, unroasted, unsalted | | 100 |
| 50 | K93 /II/20/21 |  | plastic packaging | Chile | shelled, unroasted, unsalted | | 100 |
| 51 | K94 /II/20/21 |  | plastic packaging | Chile | shelled, unroasted, unsalted | | 100 |
| 52 | K95 /II/20/21 |  | sold by weight | Poland | whole (in shell) | | 301 |
| 53 | K96 /II/20/21 |  | sold by weight | Poland | whole (in shell) | | 298 |
| 54 | K129 /II/20/21 | peanuts | plastic packaging | China | whole (in shell) | | 200 |
| 55 | K130 /II/20/21 |  | plastic packaging | China | whole (in shell) | | 200 |
| 56 | K131 /II/20/21 |  | plastic packaging | Poland | shelled, roasted, unsalted | | 250 |
| 57 | K132 /II/20/21 |  | plastic packaging | Poland | shelled, roasted, unsalted | | 250 |
| 58 | K133 /II/20/21 |  | plastic packaging | Argentina | shelled, roasted, unsalted | | 250 |
| 59 | K134 /II/20/21 |  | plastic packaging | Argentina | shelled, roasted, unsalted | | 250 |
| 60 | K135 /II/20/21 |  | plastic packaging | Argentina | shelled, roasted, unsalted | | 150 |
| 61 | K136 /II/20/21 |  | plastic packaging | Argentina | shelled, roasted, unsalted | | 150 |
| 62 | K137 /II/20/21 |  | plastic packaging | China | shelled, roasted, unsalted | | 250 |
| 63 | K138 /II/20/21 |  | plastic packaging | China | shelled, roasted, unsalted | | 200 |
| 64 | K139 /II/20/21 |  | plastic packaging | China | shelled, roasted, unsalted | | 250 |
| 65 | K140 /II/20/21 |  | plastic packaging | China | shelled, roasted, unsalted | | 250 |
| 66 | K141 /II/20/21 |  | sold by weight | Poland | whole (in shell) | | 306 |
| 67 | K142 /II/20/21 |  | sold by weight | Poland | whole (in shell) | | 308 |
| 68 | K143 /II/20/21 |  | plastic packaging | Poland | shelled, roasted, unsalted | | 140 |
| 69 | K144 /II/20/21 |  | plastic packaging | Poland | shelled, roasted, unsalted | | 140 |

**Table B.1** The content of heavy metals in nuts [mg/kg]

| **Nut type** | **Cd** | **Pb** | **Ni** | **Cr** | **Reference** |
| --- | --- | --- | --- | --- | --- |
|  | Mean  Min – Max | Mean  Min – Max | Mean  Min – Max | Mean  Min – Max |  |
| **Almonds** | 0.024  0.004–0.143 | 0.224  0.058–0.757 | 1.299  0.806–1.630 | 0.253  0.180–0.320 | *This study* |
|  | 0.01575  0.00089–0.05165 | 0.01506  0.00521–0.04401 | - | - | *Bielecka et al. [12]* |
|  | - | - | 0.83  - | - | *Ščančar et al. [20]* |
|  | 0.040  - | - | - | 0.31  - | *Yin et al. [41]* |
|  | 0.48  - | 2.81  - | - | - | *Chung et al. [47]* |
|  | 0.00069  - | 0.00064  - | 1.1  - | 0.0056  - | *Rodushkin et al. [50]* |
|  | <0.002  - | <0.006  - | - | - | *Muller et al. [51]* |
|  | 3.35  - | 0.70  - | - | 2.90  - | *Bašić et al. [52]* |
|  | 0.0478 & 0.0672  - | 0.2163 & 0.2372  - | - | - | *Tošić et al. [53]* |
|  | - | - | 12.57  - | ND | *Woźniak et al. [54]* |
| **Cashews** | 0.064  - | 0.645  - | 6.434  4.365–7.980 | 0.233  0.185–0.300 | *This study* |
|  | 0.01325  0.00105–0.09914 | 0.02005  0.00244–0.05399 | - | - | *Bielecka et al. [12]* |
|  | 0.040  - | - | - | 2.2  - | *Yin et al. [41]* |
|  | 0.40  - | 6.61  - | - | - | *Chung et al. [47]* |
|  | 0.047  - | 0.135  - | - | - | *Łoźna et al. [48]* |
|  | 0.0008  - | 0.0024  - | 6.7  - | 0.012  - | *Rodushkin et al. [50]* |
|  | 0.012  - | <0.006  - | - | - | *Muller et al. [51]* |
|  | ND | 0.5096 & 0.5336  - | - | - | *Tošić et al. [53]* |
|  | - | - | 17.98  - | ND | *Woźniak et al. [54]* |
| **Hazelnuts** | 0.019  0.005–0.060 | 0.141  0.036–0.607 | 2.371  1.338–4.044 | 0.233  0.160–0.302 | *This study* |
|  | 0.01178  0.00343–0.02322 | 0.07577  0.06452–0.09491 | - | - | *Bielecka et al. [12]* |
|  | -  0.01–0.03 | -  0.02–0.07 | -  0.81–2.97 | -  0.02–0.05 | *Özkutlu et al. [39]* |
|  | 0.24  - | 0.17  - | - | 0.22  - | *Yin et al. [41]* |
|  | - | - | -  1.35–2.25 | - | *Altundag et al. [45]* |
|  | 0.094  - | 0.149  - | - | - | *Łoźna et al. [48]* |
|  | 0.01  - | 0.0073  - | 1.3  - | 0.0044  - | *Rodushkin et al. [50]* |
|  | 0.022  - | <0.006  - | - | - | *Muller et al. [51]* |
|  | 6.95  - | 1.38  - | - | 3.34  - | *Bašić et al. [52]* |
|  | ND | 0.4157 & 0.4493  - | - | - | *Tošić et al. [53]* |
|  | - | - | 14.40  - | ND | *Woźniak et al. [54]* |
| **Peanuts** | 0.092  0.009–0.183 | 0.229  0.080–0.818 | 3.172  1.268–11.203 | 0.204  0.120–0.285 | *This study* |
|  | 0.08449  0.03858–0.29223 | 0.18855  0.06676–1.35380 | - | - | *Bielecka et al. [12]* |
|  | 0.25  0.01–5.70 | 0.16  0.02–0.98 | - | - | *Gu et al. [44]* |
|  | 0.52  - | 18.62  - | - | - | *Chung et al. [47]* |
|  | 0.063  - | 0.131  - | - | - | *Łoźna et al. [48]* |
|  | 0.0937 & 0.1231  - | 0.3617 & 0.3721  - | - | - | *Tošić et al. [53]* |
|  | - | - | 8.80  - | ND | *Woźniak et al. [54]* |
| **Walnuts** | 0.015  0.007–0.024 | 0.118  0.037–0.396 | 2.765  0.765–5.666 | 0.133  0.088–0.196 | *This study* |
|  | 0.00548  0.00015–0.03364 | 0.01308  0.00570–0.02226 | - | - | *Bielecka et al. [12]* |
|  | 0.0073  - | 0.0556  - | - | 0.1841  - | *Han et al. [27]* |
|  | - | -  0.33–0.50 | - | -  0.12–2.56 | *Taghizadeh et al. [28]* |
|  | -  0.00012–0.00020 | - | -  0.00050–0.00176 | -  0.00024–0.00207 | *Kalkışım et al. [40]* |
|  | 0.020  - | - | - | - | *Yin et al. [41]* |
|  | 0.016  0.012–0.022 | 0.0069  0.0051–0.012 | - | 0.29  0.24–0.35 | *Ni et al. [42]* |
|  | 0.0073  0.0003–0.08 | 0.0556  0.0013–0.3904 | - | 0.1841  0.0424–0.6099 | *Han et al. [43]* |
|  | 0.49  - | 3.43  - | - | - | *Chung et al. [47]* |
|  | 0.024  - | 0.150  - | - | - | *Łoźna et al. [48]* |
|  | 0.27  ND–1.20 | 0.68  ND–1.34 | 3.69  1.63–6.54 | 0.53  0.03–1.27 | *Harangozo et al. [49]* |
|  | 0.00065  - | 0.00025  - | 1.6  - | 0.0013  - | *Rodushkin et al. [50]* |
|  | <0.002  - | <0.006  - | - | - | *Muller et al. [51]* |
|  | 3.85  - | 0.64  - | - | 7.17  - | *Bašić et al. [52]* |
|  | ND | 0.1390 & 0.1501  - | - | - | *Tošić et al. [53]* |
|  | - | - | 11.76  - | ND | *Woźniak et al. [54]* |

ND – not detected

**Table C.1** Hazard Quotient (HQ) calculated for Cd, Pb, Ni, and Cr contained in peanuts

| **population** | **element** | **HQ > 1** | | | | | | | | | | | | | | | | | | | | | | | | | | | | | | | | | | |
| --- | --- | --- | --- | --- | --- | --- | --- | --- | --- | --- | --- | --- | --- | --- | --- | --- | --- | --- | --- | --- | --- | --- | --- | --- | --- | --- | --- | --- | --- | --- | --- | --- | --- | --- | --- | --- |
|  |  | **A** | | | **B** | | | **C** | | | | **D** | | | **E** | | | **F** | | | | **G** | | | | **H** | | | | **I** | | | | **J** | | |
|  |  | **I** | **II** | **III** | **I** | **II** | **III** | **I** | **II** | **III** | **I** | | **II** | **III** | **I** | **II** | **III** | **I** | **II** | **III** | **I** | | **II** | **III** | **I** | | **II** | **III** | **I** | | **II** | **III** | **I** | | **II** | **III** |
| **child**  **6-11 months** | **Cd** | 0.0098 | 0.1002 | 0.1989 | 0.0196 | 0.2004 | 0.3978 | 0.0293 | 0.3006 | 0.5967 | 0.0391 | | 0.4008 | 0.7955 | 0.0489 | 0.5010 | 0.9944 | 0.0587 | 0.6012 | 1.1933 | 0.0685 | | 0.7014 | 1.3922 | 0.0783 | | 0.8016 | 1.5911 | 0.0880 | | 0.9018 | 1.7900 | 0.0978 | | 1.0020 | 1.9889 |
|  | **Pb** | 0.0241 | 0.0693 | 0.2470 | 0.0482 | 0.1386 | 0.4940 | 0.0723 | 0.2079 | 0.7410 | 0.0964 | | 0.2771 | 0.9880 | 0.1205 | 0.3464 | 1.2350 | 0.1446 | 0.4157 | 1.4820 | 0.1687 | | 0.4850 | 1.7290 | 0.1928 | | 0.5543 | 1.9760 | 0.2170 | | 0.6236 | 2.2230 | 0.2411 | | 0.6928 | 2.4700 |
|  | **Ni** | 0.0689 | 0.1724 | 0.6088 | 0.1378 | 0.3448 | 1.2177 | 0.2067 | 0.5172 | 1.8265 | 0.2755 | | 0.6896 | 2.4353 | 0.3444 | 0.8620 | 3.0442 | 0.4133 | 1.0344 | 3.6530 | 0.4822 | | 1.2069 | 4.2618 | 0.5511 | | 1.3793 | 4.8707 | 0.6200 | | 1.5517 | 5.4795 | 0.6889 | | 1.7241 | 6.0883 |
|  | **Cr** | 0.0435 | 0.0738 | 0.1033 | 0.0870 | 0.1475 | 0.2065 | 0.1304 | 0.2213 | 0.3098 | 0.1739 | | 0.2951 | 0.4130 | 0.2174 | 0.3688 | 0.5163 | 0.2609 | 0.4426 | 0.6196 | 0.3043 | | 0.5164 | 0.7228 | 0.3478 | | 0.5901 | 0.8261 | 0.3913 | | 0.6639 | 0.9293 | 0.4348 | | 0.7377 | 1.0326 |
| **child**  **1-<2 years** | **Cd** | 0.0079 | 0.0809 | 0.1605 | 0.0158 | 0.1617 | 0.3210 | 0.0237 | 0.2426 | 0.4815 | 0.0316 | | 0.3235 | 0.6420 | 0.0395 | 0.4043 | 0.8025 | 0.0474 | 0.4852 | 0.9630 | 0.0553 | | 0.5661 | 1.1235 | 0.0632 | | 0.6469 | 1.2840 | 0.0711 | | 0.7278 | 1.4445 | 0.0789 | | 0.8087 | 1.6050 |
|  | **Pb** | 0.0195 | 0.0559 | 0.1993 | 0.0389 | 0.1118 | 0.3987 | 0.0584 | 0.1677 | 0.5980 | 0.0778 | | 0.2237 | 0.7973 | 0.0973 | 0.2796 | 0.9967 | 0.1167 | 0.3355 | 1.1960 | 0.1362 | | 0.3914 | 1.3954 | 0.1556 | | 0.4473 | 1.5947 | 0.1751 | | 0.5032 | 1.7940 | 0.1945 | | 0.5591 | 1.9934 |
|  | **Ni** | 0.0556 | 0.1391 | 0.4913 | 0.1112 | 0.2783 | 0.9827 | 0.1668 | 0.4174 | 1.4740 | 0.2224 | | 0.5565 | 1.9654 | 0.2780 | 0.6957 | 2.4567 | 0.3336 | 0.8348 | 2.9480 | 0.3891 | | 0.9740 | 3.4394 | 0.4447 | | 1.1131 | 3.9307 | 0.5003 | | 1.2522 | 4.4220 | 0.5559 | | 1.3914 | 4.9134 |
|  | **Cr** | 0.0351 | 0.0595 | 0.0833 | 0.0702 | 0.1191 | 0.1667 | 0.1053 | 0.1786 | 0.2500 | 0.1404 | | 0.2381 | 0.3333 | 0.1754 | 0.2977 | 0.4167 | 0.2105 | 0.3572 | 0.5000 | 0.2456 | | 0.4167 | 0.5833 | 0.2807 | | 0.4762 | 0.6667 | 0.3158 | | 0.5358 | 0.7500 | 0.3509 | | 0.5953 | 0.8333 |
| **child**  **2-<3 years** | **Cd** | 0.0065 | 0.0668 | 0.1326 | 0.0130 | 0.1336 | 0.2652 | 0.0196 | 0.2004 | 0.3978 | 0.0261 | | 0.2672 | 0.5304 | 0.0326 | 0.3340 | 0.6630 | 0.0391 | 0.4008 | 0.7955 | 0.0457 | | 0.4676 | 0.9281 | 0.0522 | | 0.5344 | 1.0607 | 0.0587 | | 0.6012 | 1.1933 | 0.0652 | | 0.6680 | 1.3259 |
|  | **Pb** | 0.0161 | 0.0462 | 0.1647 | 0.0321 | 0.0924 | 0.3293 | 0.0482 | 0.1386 | 0.4940 | 0.0643 | | 0.1848 | 0.6587 | 0.0804 | 0.2309 | 0.8233 | 0.0964 | 0.2771 | 0.9880 | 0.1125 | | 0.3233 | 1.1527 | 0.1286 | | 0.3695 | 1.3174 | 0.1446 | | 0.4157 | 1.4820 | 0.1607 | | 0.4619 | 1.6467 |
|  | **Ni** | 0.0459 | 0.1149 | 0.4059 | 0.0918 | 0.2299 | 0.8118 | 0.1378 | 0.3448 | 1.2177 | 0.1837 | | 0.4598 | 1.6236 | 0.2296 | 0.5747 | 2.0294 | 0.2755 | 0.6896 | 2.4353 | 0.3215 | | 0.8046 | 2.8412 | 0.3674 | | 0.9195 | 3.2471 | 0.4133 | | 1.0344 | 3.6530 | 0.4592 | | 1.1494 | 4.0589 |
|  | **Cr** | 0.0290 | 0.0492 | 0.0688 | 0.0580 | 0.0984 | 0.1377 | 0.0870 | 0.1475 | 0.2065 | 0.1159 | | 0.1967 | 0.2754 | 0.1449 | 0.2459 | 0.3442 | 0.1739 | 0.2951 | 0.4130 | 0.2029 | | 0.3442 | 0.4819 | 0.2319 | | 0.3934 | 0.5507 | 0.2609 | | 0.4426 | 0.6196 | 0.2899 | | 0.4918 | 0.6884 |
| **child**  **3-<6 years** | **Cd** | 0.0048 | 0.0496 | 0.0984 | 0.0097 | 0.0991 | 0.1967 | 0.0145 | 0.1487 | 0.2951 | 0.0194 | | 0.1983 | 0.3935 | 0.0242 | 0.2478 | 0.4919 | 0.0290 | 0.2974 | 0.5902 | 0.0339 | | 0.3469 | 0.6886 | 0.0387 | | 0.3965 | 0.7870 | 0.0435 | | 0.4461 | 0.8854 | 0.0484 | | 0.4956 | 0.9837 |
|  | **Pb** | 0.0119 | 0.0343 | 0.1222 | 0.0238 | 0.0685 | 0.2443 | 0.0358 | 0.1028 | 0.3665 | 0.0477 | | 0.1371 | 0.4887 | 0.0596 | 0.1713 | 0.6109 | 0.0715 | 0.2056 | 0.7330 | 0.0835 | | 0.2399 | 0.8552 | 0.0954 | | 0.2742 | 0.9774 | 0.1073 | | 0.3084 | 1.0996 | 0.1192 | | 0.3427 | 1.2217 |
|  | **Ni** | 0.0341 | 0.0853 | 0.3011 | 0.0681 | 0.1706 | 0.6023 | 0.1022 | 0.2558 | 0.9034 | 0.1363 | | 0.3411 | 1.2046 | 0.1704 | 0.4264 | 1.5057 | 0.2044 | 0.5117 | 1.8069 | 0.2385 | | 0.5969 | 2.1080 | 0.2726 | | 0.6822 | 2.4091 | 0.3067 | | 0.7675 | 2.7103 | 0.3407 | | 0.8528 | 3.0114 |
|  | **Cr** | 0.0215 | 0.0365 | 0.0511 | 0.0430 | 0.0730 | 0.1022 | 0.0645 | 0.1095 | 0.1532 | 0.0860 | | 0.1459 | 0.2043 | 0.1075 | 0.1824 | 0.2554 | 0.1290 | 0.2189 | 0.3065 | 0.1505 | | 0.2554 | 0.3575 | 0.1720 | | 0.2919 | 0.4086 | 0.1935 | | 0.3284 | 0.4597 | 0.2151 | | 0.3649 | 0.5108 |
| **child**  **6-<11 years** | **Cd** | 0.0028 | 0.0290 | 0.0575 | 0.0057 | 0.0580 | 0.1151 | 0.0085 | 0.0870 | 0.1726 | 0.0113 | | 0.1160 | 0.2302 | 0.0142 | 0.1449 | 0.2877 | 0.0170 | 0.1739 | 0.3452 | 0.0198 | | 0.2029 | 0.4028 | 0.0226 | | 0.2319 | 0.4603 | 0.0255 | | 0.2609 | 0.5179 | 0.0283 | | 0.2899 | 0.5754 |
|  | **Pb** | 0.0070 | 0.0200 | 0.0715 | 0.0139 | 0.0401 | 0.1429 | 0.0209 | 0.0601 | 0.2144 | 0.0279 | | 0.0802 | 0.2858 | 0.0349 | 0.1002 | 0.3573 | 0.0418 | 0.1203 | 0.4288 | 0.0488 | | 0.1403 | 0.5002 | 0.0558 | | 0.1604 | 0.5717 | 0.0628 | | 0.1804 | 0.6431 | 0.0697 | | 0.2004 | 0.7146 |
|  | **Ni** | 0.0199 | 0.0499 | 0.1761 | 0.0399 | 0.0998 | 0.3523 | 0.0598 | 0.1496 | 0.5284 | 0.0797 | | 0.1995 | 0.7046 | 0.0996 | 0.2494 | 0.8807 | 0.1196 | 0.2993 | 1.0568 | 0.1395 | | 0.3492 | 1.2330 | 0.1594 | | 0.3990 | 1.4091 | 0.1794 | | 0.4489 | 1.5853 | 0.1993 | | 0.4988 | 1.7614 |
|  | **Cr** | 0.0126 | 0.0213 | 0.0299 | 0.0252 | 0.0427 | 0.0597 | 0.0377 | 0.0640 | 0.0896 | 0.0503 | | 0.0854 | 0.1195 | 0.0629 | 0.1067 | 0.1494 | 0.0755 | 0.1280 | 0.1792 | 0.0881 | | 0.1494 | 0.2091 | 0.1006 | | 0.1707 | 0.2390 | 0.1132 | | 0.1921 | 0.2689 | 0.1258 | | 0.2134 | 0.2987 |
| **child**  **11-<16 years** | **Cd** | 0.0016 | 0.0162 | 0.0322 | 0.0032 | 0.0325 | 0.0644 | 0.0048 | 0.0487 | 0.0966 | 0.0063 | | 0.0649 | 0.1289 | 0.0079 | 0.0812 | 0.1611 | 0.0095 | 0.0974 | 0.1933 | 0.0111 | | 0.1136 | 0.2255 | 0.0127 | | 0.1298 | 0.2577 | 0.0143 | | 0.1461 | 0.2899 | 0.0158 | | 0.1623 | 0.3221 |
|  | **Pb** | 0.0039 | 0.0112 | 0.0400 | 0.0078 | 0.0224 | 0.0800 | 0.0117 | 0.0337 | 0.1200 | 0.0156 | | 0.0449 | 0.1600 | 0.0195 | 0.0561 | 0.2000 | 0.0234 | 0.0673 | 0.2400 | 0.0273 | | 0.0786 | 0.2801 | 0.0312 | | 0.0898 | 0.3201 | 0.0351 | | 0.1010 | 0.3601 | 0.0390 | | 0.1122 | 0.4001 |
|  | **Ni** | 0.0112 | 0.0279 | 0.0986 | 0.0223 | 0.0559 | 0.1972 | 0.0335 | 0.0838 | 0.2958 | 0.0446 | | 0.1117 | 0.3945 | 0.0558 | 0.1396 | 0.4931 | 0.0669 | 0.1676 | 0.5917 | 0.0781 | | 0.1955 | 0.6903 | 0.0893 | | 0.2234 | 0.7889 | 0.1004 | | 0.2513 | 0.8875 | 0.1116 | | 0.2793 | 0.9861 |
|  | **Cr** | 0.0070 | 0.0119 | 0.0167 | 0.0141 | 0.0239 | 0.0335 | 0.0211 | 0.0358 | 0.0502 | 0.0282 | | 0.0478 | 0.0669 | 0.0352 | 0.0597 | 0.0836 | 0.0423 | 0.0717 | 0.1004 | 0.0493 | | 0.0836 | 0.1171 | 0.0563 | | 0.0956 | 0.1338 | 0.0634 | | 0.1075 | 0.1505 | 0.0704 | | 0.1195 | 0.1673 |
| **child**  **16-<21 years** | **Cd** | 0.0013 | 0.0129 | 0.0256 | 0.0025 | 0.0258 | 0.0511 | 0.0038 | 0.0386 | 0.0767 | 0.0050 | | 0.0515 | 0.1022 | 0.0063 | 0.0644 | 0.1278 | 0.0075 | 0.0773 | 0.1533 | 0.0088 | | 0.0901 | 0.1789 | 0.0101 | | 0.1030 | 0.2044 | 0.0113 | | 0.1159 | 0.2300 | 0.0126 | | 0.1288 | 0.2556 |
|  | **Pb** | 0.0031 | 0.0089 | 0.0317 | 0.0062 | 0.0178 | 0.0635 | 0.0093 | 0.0267 | 0.0952 | 0.0124 | | 0.0356 | 0.1270 | 0.0155 | 0.0445 | 0.1587 | 0.0186 | 0.0534 | 0.1904 | 0.0217 | | 0.0623 | 0.2222 | 0.0248 | | 0.0712 | 0.2539 | 0.0279 | | 0.0801 | 0.2856 | 0.0310 | | 0.0890 | 0.3174 |
|  | **Ni** | 0.0089 | 0.0222 | 0.0782 | 0.0177 | 0.0443 | 0.1565 | 0.0266 | 0.0665 | 0.2347 | 0.0354 | | 0.0886 | 0.3129 | 0.0443 | 0.1108 | 0.3911 | 0.0531 | 0.1329 | 0.4694 | 0.0620 | | 0.1551 | 0.5476 | 0.0708 | | 0.1772 | 0.6258 | 0.0797 | | 0.1994 | 0.7041 | 0.0885 | | 0.2215 | 0.7823 |
|  | **Cr** | 0.0056 | 0.0095 | 0.0133 | 0.0112 | 0.0190 | 0.0265 | 0.0168 | 0.0284 | 0.0398 | 0.0223 | | 0.0379 | 0.0531 | 0.0279 | 0.0474 | 0.0663 | 0.0335 | 0.0569 | 0.0796 | 0.0391 | | 0.0663 | 0.0929 | 0.0447 | | 0.0758 | 0.1061 | 0.0503 | | 0.0853 | 0.1194 | 0.0559 | | 0.0948 | 0.1327 |
| **adult** | **Cd** | 0.0011 | 0.0115 | 0.0229 | 0.0023 | 0.0230 | 0.0457 | 0.0034 | 0.0346 | 0.0686 | 0.0045 | | 0.0461 | 0.0915 | 0.0056 | 0.0576 | 0.1144 | 0.0068 | 0.0691 | 0.1372 | 0.0079 | | 0.0807 | 0.1601 | 0.0090 | | 0.0922 | 0.1830 | 0.0101 | | 0.1037 | 0.2058 | 0.0113 | | 0.1152 | 0.2287 |
|  | **Pb** | 0.0028 | 0.0080 | 0.0284 | 0.0055 | 0.0159 | 0.0568 | 0.0083 | 0.0239 | 0.0852 | 0.0111 | | 0.0319 | 0.1136 | 0.0139 | 0.0398 | 0.1420 | 0.0166 | 0.0478 | 0.1704 | 0.0194 | | 0.0558 | 0.1988 | 0.0222 | | 0.0637 | 0.2272 | 0.0249 | | 0.0717 | 0.2556 | 0.0277 | | 0.0797 | 0.2841 |
|  | **Ni** | 0.0079 | 0.0198 | 0.0700 | 0.0158 | 0.0397 | 0.1400 | 0.0238 | 0.0595 | 0.2100 | 0.0317 | | 0.0793 | 0.2801 | 0.0396 | 0.0991 | 0.3501 | 0.0475 | 0.1190 | 0.4201 | 0.0555 | | 0.1388 | 0.4901 | 0.0634 | | 0.1586 | 0.5601 | 0.0713 | | 0.1784 | 0.6301 | 0.0792 | | 0.1983 | 0.7002 |
|  | **Cr** | 0.0050 | 0.0085 | 0.0119 | 0.0100 | 0.0170 | 0.0238 | 0.0150 | 0.0254 | 0.0356 | 0.0200 | | 0.0339 | 0.0475 | 0.0250 | 0.0424 | 0.0594 | 0.0300 | 0.0509 | 0.0713 | 0.0350 | | 0.0594 | 0.0831 | 0.0400 | | 0.0679 | 0.0950 | 0.0450 | | 0.0763 | 0.1069 | 0.0500 | | 0.0848 | 0.1188 |

**Table C.2** Hazard Quotient (HQ) calculated for Cd, Pb, Ni, and Cr contained in almonds

| **population** | **element** | **HQ > 1** | | | | | | | | | | | | | | | | | | | | | | | | | | | | | | | | | | | |
| --- | --- | --- | --- | --- | --- | --- | --- | --- | --- | --- | --- | --- | --- | --- | --- | --- | --- | --- | --- | --- | --- | --- | --- | --- | --- | --- | --- | --- | --- | --- | --- | --- | --- | --- | --- | --- | --- |
|  |  | **A** | | | **B** | | | **C** | | | **D** | | | | **E** | | | | **F** | | | | **G** | | | | **H** | | | | **I** | | | | **J** | | |
|  |  | **I** | **II** | **III** | **I** | **II** | **III** | **I** | **II** | **III** | **I** | **II** | **III** | **I** | | **II** | **III** | **I** | | **II** | **III** | **I** | | **II** | **III** | **I** | | **II** | **III** | **I** | | **II** | **III** | **I** | | **II** | **III** |
| **child**  **6-11 months** | **Cd** | 0.0047 | 0.0264 | 0.1554 | 0.0094 | 0.0529 | 0.3109 | 0.0141 | 0.0793 | 0.4663 | 0.0187 | 0.1058 | 0.6217 | 0.0234 | | 0.1322 | 0.7772 | 0.0281 | | 0.1587 | 0.9326 | 0.0328 | | 0.1851 | 1.0880 | 0.0375 | | 0.2116 | 1.2435 | 0.0422 | | 0.2380 | 1.3989 | 0.0468 | | 0.2644 | 1.5543 |
|  | **Pb** | 0.0175 | 0.0676 | 0.2286 | 0.0350 | 0.1351 | 0.4572 | 0.0525 | 0.2027 | 0.6858 | 0.0700 | 0.2703 | 0.9144 | 0.0876 | | 0.3378 | 1.1430 | 0.1051 | | 0.4054 | 1.3717 | 0.1226 | | 0.4729 | 1.6003 | 0.1401 | | 0.5405 | 1.8289 | 0.1576 | | 0.6081 | 2.0575 | 0.1751 | | 0.6756 | 2.2861 |
|  | **Ni** | 0.0438 | 0.0706 | 0.0886 | 0.0876 | 0.1412 | 0.1772 | 0.1314 | 0.2118 | 0.2658 | 0.1752 | 0.2824 | 0.3543 | 0.2190 | | 0.3530 | 0.4429 | 0.2628 | | 0.4236 | 0.5315 | 0.3066 | | 0.4942 | 0.6201 | 0.3504 | | 0.5648 | 0.7087 | 0.3942 | | 0.6354 | 0.7973 | 0.4380 | | 0.7060 | 0.8859 |
|  | **Cr** | 0.0652 | 0.0916 | 0.1159 | 0.1304 | 0.1831 | 0.2319 | 0.1957 | 0.2747 | 0.3478 | 0.2609 | 0.3662 | 0.4638 | 0.3261 | | 0.4578 | 0.5797 | 0.3913 | | 0.5493 | 0.6957 | 0.4565 | | 0.6409 | 0.8116 | 0.5217 | | 0.7325 | 0.9275 | 0.5870 | | 0.8240 | 1.0435 | 0.6522 | | 0.9156 | 1.1594 |
| **child**  **1-<2**  **years** | **Cd** | 0.0038 | 0.0213 | 0.1254 | 0.0076 | 0.0427 | 0.2509 | 0.0113 | 0.0640 | 0.3763 | 0.0151 | 0.0854 | 0.5018 | 0.0189 | | 0.1067 | 0.6272 | 0.0227 | | 0.1280 | 0.7526 | 0.0265 | | 0.1494 | 0.8781 | 0.0302 | | 0.1707 | 1.0035 | 0.0340 | | 0.1921 | 1.1289 | 0.0378 | | 0.2134 | 1.2544 |
|  | **Pb** | 0.0141 | 0.0545 | 0.1845 | 0.0283 | 0.1091 | 0.3690 | 0.0424 | 0.1636 | 0.5535 | 0.0565 | 0.2181 | 0.7380 | 0.0707 | | 0.2726 | 0.9225 | 0.0848 | | 0.3272 | 1.1069 | 0.0989 | | 0.3817 | 1.2914 | 0.1131 | | 0.4362 | 1.4759 | 0.1272 | | 0.4907 | 1.6604 | 0.1413 | | 0.5453 | 1.8449 |
|  | **Ni** | 0.0354 | 0.0570 | 0.0715 | 0.0707 | 0.1139 | 0.1430 | 0.1061 | 0.1709 | 0.2145 | 0.1414 | 0.2279 | 0.2860 | 0.1768 | | 0.2849 | 0.3575 | 0.2121 | | 0.3418 | 0.4289 | 0.2475 | | 0.3988 | 0.5004 | 0.2828 | | 0.4558 | 0.5719 | 0.3182 | | 0.5128 | 0.6434 | 0.3535 | | 0.5697 | 0.7149 |
|  | **Cr** | 0.0526 | 0.0739 | 0.0936 | 0.1053 | 0.1478 | 0.1871 | 0.1579 | 0.2217 | 0.2807 | 0.2105 | 0.2956 | 0.3743 | 0.2632 | | 0.3694 | 0.4678 | 0.3158 | | 0.4433 | 0.5614 | 0.3684 | | 0.5172 | 0.6550 | 0.4211 | | 0.5911 | 0.7485 | 0.4737 | | 0.6650 | 0.8421 | 0.5263 | | 0.7389 | 0.9357 |
| **child**  **2-<3**  **years** | **Cd** | 0.0031 | 0.0176 | 0.1036 | 0.0062 | 0.0353 | 0.2072 | 0.0094 | 0.0529 | 0.3109 | 0.0125 | 0.0705 | 0.4145 | 0.0156 | | 0.0881 | 0.5181 | 0.0187 | | 0.1058 | 0.6217 | 0.0219 | | 0.1234 | 0.7254 | 0.0250 | | 0.1410 | 0.8290 | 0.0281 | | 0.1587 | 0.9326 | 0.0312 | | 0.1763 | 1.0362 |
|  | **Pb** | 0.0117 | 0.0450 | 0.1524 | 0.0233 | 0.0901 | 0.3048 | 0.0350 | 0.1351 | 0.4572 | 0.0467 | 0.1802 | 0.6096 | 0.0584 | | 0.2252 | 0.7620 | 0.0700 | | 0.2703 | 0.9144 | 0.0817 | | 0.3153 | 1.0668 | 0.0934 | | 0.3603 | 1.2192 | 0.1051 | | 0.4054 | 1.3717 | 0.1167 | | 0.4504 | 1.5241 |
|  | **Ni** | 0.0292 | 0.0471 | 0.0591 | 0.0584 | 0.0941 | 0.1181 | 0.0876 | 0.1412 | 0.1772 | 0.1168 | 0.1883 | 0.2362 | 0.1460 | | 0.2353 | 0.2953 | 0.1752 | | 0.2824 | 0.3543 | 0.2044 | | 0.3295 | 0.4134 | 0.2336 | | 0.3765 | 0.4725 | 0.2628 | | 0.4236 | 0.5315 | 0.2920 | | 0.4707 | 0.5906 |
|  | **Cr** | 0.0435 | 0.0610 | 0.0773 | 0.0870 | 0.1221 | 0.1546 | 0.1304 | 0.1831 | 0.2319 | 0.1739 | 0.2442 | 0.3092 | 0.2174 | | 0.3052 | 0.3865 | 0.2609 | | 0.3662 | 0.4638 | 0.3043 | | 0.4273 | 0.5411 | 0.3478 | | 0.4883 | 0.6184 | 0.3913 | | 0.5493 | 0.6957 | 0.4348 | | 0.6104 | 0.7729 |
| **child**  **3-<6**  **years** | **Cd** | 0.0023 | 0.0131 | 0.0769 | 0.0046 | 0.0262 | 0.1538 | 0.0070 | 0.0392 | 0.2306 | 0.0093 | 0.0523 | 0.3075 | 0.0116 | | 0.0654 | 0.3844 | 0.0139 | | 0.0785 | 0.4613 | 0.0162 | | 0.0916 | 0.5382 | 0.0185 | | 0.1046 | 0.6151 | 0.0209 | | 0.1177 | 0.6919 | 0.0232 | | 0.1308 | 0.7688 |
|  | **Pb** | 0.0087 | 0.0334 | 0.1131 | 0.0173 | 0.0668 | 0.2262 | 0.0260 | 0.1003 | 0.3392 | 0.0346 | 0.1337 | 0.4523 | 0.0433 | | 0.1671 | 0.5654 | 0.0520 | | 0.2005 | 0.6785 | 0.0606 | | 0.2339 | 0.7915 | 0.0693 | | 0.2673 | 0.9046 | 0.0780 | | 0.3008 | 1.0177 | 0.0866 | | 0.3342 | 1.1308 |
|  | **Ni** | 0.0217 | 0.0349 | 0.0438 | 0.0433 | 0.0698 | 0.0876 | 0.0650 | 0.1048 | 0.1315 | 0.0867 | 0.1397 | 0.1753 | 0.1083 | | 0.1746 | 0.2191 | 0.1300 | | 0.2095 | 0.2629 | 0.1517 | | 0.2444 | 0.3067 | 0.1733 | | 0.2794 | 0.3505 | 0.1950 | | 0.3143 | 0.3944 | 0.2167 | | 0.3492 | 0.4382 |
|  | **Cr** | 0.0323 | 0.0453 | 0.0573 | 0.0645 | 0.0906 | 0.1147 | 0.0968 | 0.1359 | 0.1720 | 0.1290 | 0.1811 | 0.2294 | 0.1613 | | 0.2264 | 0.2867 | 0.1935 | | 0.2717 | 0.3441 | 0.2258 | | 0.3170 | 0.4014 | 0.2581 | | 0.3623 | 0.4588 | 0.2903 | | 0.4076 | 0.5161 | 0.3226 | | 0.4529 | 0.5735 |
| **child**  **6-<11 years** | **Cd** | 0.0014 | 0.0077 | 0.0450 | 0.0027 | 0.0153 | 0.0899 | 0.0041 | 0.0230 | 0.1349 | 0.0054 | 0.0306 | 0.1799 | 0.0068 | | 0.0383 | 0.2248 | 0.0081 | | 0.0459 | 0.2698 | 0.0095 | | 0.0536 | 0.3148 | 0.0108 | | 0.0612 | 0.3597 | 0.0122 | | 0.0689 | 0.4047 | 0.0136 | | 0.0765 | 0.4497 |
|  | **Pb** | 0.0051 | 0.0195 | 0.0661 | 0.0101 | 0.0391 | 0.1323 | 0.0152 | 0.0586 | 0.1984 | 0.0203 | 0.0782 | 0.2646 | 0.0253 | | 0.0977 | 0.3307 | 0.0304 | | 0.1173 | 0.3968 | 0.0355 | | 0.1368 | 0.4630 | 0.0405 | | 0.1564 | 0.5291 | 0.0456 | | 0.1759 | 0.5952 | 0.0507 | | 0.1955 | 0.6614 |
|  | **Ni** | 0.0127 | 0.0204 | 0.0256 | 0.0253 | 0.0408 | 0.0513 | 0.0380 | 0.0613 | 0.0769 | 0.0507 | 0.0817 | 0.1025 | 0.0634 | | 0.1021 | 0.1281 | 0.0760 | | 0.1225 | 0.1538 | 0.0887 | | 0.1430 | 0.1794 | 0.1014 | | 0.1634 | 0.2050 | 0.1141 | | 0.1838 | 0.2307 | 0.1267 | | 0.2042 | 0.2563 |
|  | **Cr** | 0.0189 | 0.0265 | 0.0335 | 0.0377 | 0.0530 | 0.0671 | 0.0566 | 0.0795 | 0.1006 | 0.0755 | 0.1060 | 0.1342 | 0.0943 | | 0.1324 | 0.1677 | 0.1132 | | 0.1589 | 0.2013 | 0.1321 | | 0.1854 | 0.2348 | 0.1509 | | 0.2119 | 0.2683 | 0.1698 | | 0.2384 | 0.3019 | 0.1887 | | 0.2649 | 0.3354 |
| **child**  **11-<16 years** | **Cd** | 0.0008 | 0.0043 | 0.0252 | 0.0015 | 0.0086 | 0.0504 | 0.0023 | 0.0128 | 0.0755 | 0.0030 | 0.0171 | 0.1007 | 0.0038 | | 0.0214 | 0.1259 | 0.0046 | | 0.0257 | 0.1511 | 0.0053 | | 0.0300 | 0.1762 | 0.0061 | | 0.0343 | 0.2014 | 0.0068 | | 0.0385 | 0.2266 | 0.0076 | | 0.0428 | 0.2518 |
|  | **Pb** | 0.0028 | 0.0109 | 0.0370 | 0.0057 | 0.0219 | 0.0741 | 0.0085 | 0.0328 | 0.1111 | 0.0113 | 0.0438 | 0.1481 | 0.0142 | | 0.0547 | 0.1851 | 0.0170 | | 0.0657 | 0.2222 | 0.0199 | | 0.0766 | 0.2592 | 0.0227 | | 0.0875 | 0.2962 | 0.0255 | | 0.0985 | 0.3333 | 0.0284 | | 0.1094 | 0.3703 |
|  | **Ni** | 0.0071 | 0.0114 | 0.0143 | 0.0142 | 0.0229 | 0.0287 | 0.0213 | 0.0343 | 0.0430 | 0.0284 | 0.0457 | 0.0574 | 0.0355 | | 0.0572 | 0.0717 | 0.0426 | | 0.0686 | 0.0861 | 0.0497 | | 0.0800 | 0.1004 | 0.0568 | | 0.0915 | 0.1148 | 0.0639 | | 0.1029 | 0.1291 | 0.0710 | | 0.1143 | 0.1435 |
|  | **Cr** | 0.0106 | 0.0148 | 0.0188 | 0.0211 | 0.0297 | 0.0376 | 0.0317 | 0.0445 | 0.0563 | 0.0423 | 0.0593 | 0.0751 | 0.0528 | | 0.0741 | 0.0939 | 0.0634 | | 0.0890 | 0.1127 | 0.0739 | | 0.1038 | 0.1315 | 0.0845 | | 0.1186 | 0.1502 | 0.0951 | | 0.1335 | 0.1690 | 0.1056 | | 0.1483 | 0.1878 |
| **child**  **16-<21 years** | **Cd** | 0.0006 | 0.0034 | 0.0200 | 0.0012 | 0.0068 | 0.0399 | 0.0018 | 0.0102 | 0.0599 | 0.0024 | 0.0136 | 0.0799 | 0.0030 | | 0.0170 | 0.0999 | 0.0036 | | 0.0204 | 0.1198 | 0.0042 | | 0.0238 | 0.1398 | 0.0048 | | 0.0272 | 0.1598 | 0.0054 | | 0.0306 | 0.1797 | 0.0060 | | 0.0340 | 0.1997 |
|  | **Pb** | 0.0023 | 0.0087 | 0.0294 | 0.0045 | 0.0174 | 0.0587 | 0.0068 | 0.0260 | 0.0881 | 0.0090 | 0.0347 | 0.1175 | 0.0113 | | 0.0434 | 0.1469 | 0.0135 | | 0.0521 | 0.1762 | 0.0158 | | 0.0608 | 0.2056 | 0.0180 | | 0.0695 | 0.2350 | 0.0203 | | 0.0781 | 0.2644 | 0.0225 | | 0.0868 | 0.2937 |
|  | **Ni** | 0.0056 | 0.0091 | 0.0114 | 0.0113 | 0.0181 | 0.0228 | 0.0169 | 0.0272 | 0.0341 | 0.0225 | 0.0363 | 0.0455 | 0.0281 | | 0.0454 | 0.0569 | 0.0338 | | 0.0544 | 0.0683 | 0.0394 | | 0.0635 | 0.0797 | 0.0450 | | 0.0726 | 0.0911 | 0.0507 | | 0.0816 | 0.1024 | 0.0563 | | 0.0907 | 0.1138 |
|  | **Cr** | 0.0084 | 0.0118 | 0.0149 | 0.0168 | 0.0235 | 0.0298 | 0.0251 | 0.0353 | 0.0447 | 0.0335 | 0.0471 | 0.0596 | 0.0419 | | 0.0588 | 0.0745 | 0.0503 | | 0.0706 | 0.0894 | 0.0587 | | 0.0824 | 0.1043 | 0.0670 | | 0.0941 | 0.1192 | 0.0754 | | 0.1059 | 0.1341 | 0.0838 | | 0.1176 | 0.1490 |
| **adult** | **Cd** | 0.0005 | 0.0030 | 0.0179 | 0.0011 | 0.0061 | 0.0358 | 0.0016 | 0.0091 | 0.0536 | 0.0022 | 0.0122 | 0.0715 | 0.0027 | | 0.0152 | 0.0894 | 0.0032 | | 0.0182 | 0.1073 | 0.0038 | | 0.0213 | 0.1251 | 0.0043 | | 0.0243 | 0.1430 | 0.0048 | | 0.0274 | 0.1609 | 0.0054 | | 0.0304 | 0.1788 |
|  | **Pb** | 0.0020 | 0.0078 | 0.0263 | 0.0040 | 0.0155 | 0.0526 | 0.0060 | 0.0233 | 0.0789 | 0.0081 | 0.0311 | 0.1052 | 0.0101 | | 0.0388 | 0.1314 | 0.0121 | | 0.0466 | 0.1577 | 0.0141 | | 0.0544 | 0.1840 | 0.0161 | | 0.0622 | 0.2103 | 0.0181 | | 0.0699 | 0.2366 | 0.0201 | | 0.0777 | 0.2629 |
|  | **Ni** | 0.0050 | 0.0081 | 0.0102 | 0.0101 | 0.0162 | 0.0204 | 0.0151 | 0.0244 | 0.0306 | 0.0202 | 0.0325 | 0.0408 | 0.0252 | | 0.0406 | 0.0509 | 0.0302 | | 0.0487 | 0.0611 | 0.0353 | | 0.0568 | 0.0713 | 0.0403 | | 0.0650 | 0.0815 | 0.0453 | | 0.0731 | 0.0917 | 0.0504 | | 0.0812 | 0.1019 |
|  | **Cr** | 0.0075 | 0.0105 | 0.0133 | 0.0150 | 0.0211 | 0.0267 | 0.0225 | 0.0316 | 0.0400 | 0.0300 | 0.0421 | 0.0533 | 0.0375 | | 0.0526 | 0.0667 | 0.0450 | | 0.0632 | 0.0800 | 0.0525 | | 0.0737 | 0.0933 | 0.0600 | | 0.0842 | 0.1067 | 0.0675 | | 0.0948 | 0.1200 | 0.0750 | | 0.1053 | 0.1333 |

**Table C.3** Hazard Quotient (HQ) calculated for Cd, Pb, Ni, and Cr contained in hazelnuts

| **population** | **element** | **HQ > 1** | | | | | | | | | | | | | | | | | | | | | | | | | | | | | | | | | | | | | | |  |
| --- | --- | --- | --- | --- | --- | --- | --- | --- | --- | --- | --- | --- | --- | --- | --- | --- | --- | --- | --- | --- | --- | --- | --- | --- | --- | --- | --- | --- | --- | --- | --- | --- | --- | --- | --- | --- | --- | --- | --- | --- | --- |
|  |  | **A** | | | **B** | | | | **C** | | | | **D** | | | | **E** | | | | **F** | | | | **G** | | | | **H** | | | | **I** | | | | **J** | | | |  |
|  |  | **I** | **II** | **III** | | **I** | **II** | **III** | | **I** | **II** | **III** | | **I** | **II** | **III** | | **I** | **II** | **III** | | **I** | **II** | **III** | | **I** | **II** | **III** | | **I** | **II** | **III** | | **I** | **II** | **III** | | **I** | **II** | **III** | |
| **child**  **6-11 months** | **Cd** | 0.0056 | 0.0207 | 0.0648 | | 0.0111 | 0.0413 | 0.1295 | | 0.0167 | 0.0620 | 0.1943 | | 0.0222 | 0.0827 | 0.2590 | | 0.0278 | 0.1033 | 0.3238 | | 0.0333 | 0.1240 | 0.3885 | | 0.0389 | 0.1446 | 0.4533 | | 0.0444 | 0.1653 | 0.5180 | | 0.0500 | 0.1860 | 0.5828 | | 0.0555 | 0.2066 | 0.6475 | |
|  | **Pb** | 0.0108 | 0.0426 | 0.1834 | | 0.0216 | 0.0852 | 0.3668 | | 0.0324 | 0.1278 | 0.5502 | | 0.0432 | 0.1704 | 0.7337 | | 0.0541 | 0.2130 | 0.9171 | | 0.0649 | 0.2556 | 1.1005 | | 0.0757 | 0.2982 | 1.2839 | | 0.0865 | 0.3408 | 1.4673 | | 0.0973 | 0.3834 | 1.6507 | | 0.1081 | 0.4260 | 1.8341 | |
|  | **Ni** | 0.0727 | 0.1288 | 0.2198 | | 0.1454 | 0.2577 | 0.4396 | | 0.2182 | 0.3865 | 0.6593 | | 0.2909 | 0.5153 | 0.8791 | | 0.3636 | 0.6442 | 1.0989 | | 0.4363 | 0.7730 | 1.3187 | | 0.5090 | 0.9018 | 1.5385 | | 0.5817 | 1.0307 | 1.7583 | | 0.6545 | 1.1595 | 1.9780 | | 0.7272 | 1.2883 | 2.1978 | |
|  | **Cr** | 0.0580 | 0.0845 | 0.1094 | | 0.1159 | 0.1690 | 0.2188 | | 0.1739 | 0.2535 | 0.3283 | | 0.2319 | 0.3380 | 0.4377 | | 0.2899 | 0.4225 | 0.5471 | | 0.3478 | 0.5070 | 0.6565 | | 0.4058 | 0.5915 | 0.7659 | | 0.4638 | 0.6760 | 0.8754 | | 0.5217 | 0.7605 | 0.9848 | | 0.5797 | 0.8450 | 1.0942 | |
| **child**  **1-<2 years** | **Cd** | 0.0045 | 0.0167 | 0.0523 | | 0.0090 | 0.0334 | 0.1045 | | 0.0134 | 0.0500 | 0.1568 | | 0.0179 | 0.0667 | 0.2090 | | 0.0224 | 0.0834 | 0.2613 | | 0.0269 | 0.1001 | 0.3135 | | 0.0314 | 0.1167 | 0.3658 | | 0.0359 | 0.1334 | 0.4180 | | 0.0403 | 0.1501 | 0.4703 | | 0.0448 | 0.1668 | 0.5225 | |
|  | **Pb** | 0.0087 | 0.0344 | 0.1480 | | 0.0174 | 0.0688 | 0.2960 | | 0.0262 | 0.1031 | 0.4441 | | 0.0349 | 0.1375 | 0.5921 | | 0.0436 | 0.1719 | 0.7401 | | 0.0523 | 0.2063 | 0.8881 | | 0.0611 | 0.2407 | 1.0361 | | 0.0698 | 0.2750 | 1.1841 | | 0.0785 | 0.3094 | 1.3322 | | 0.0872 | 0.3438 | 1.4802 | |
|  | **Ni** | 0.0587 | 0.1040 | 0.1774 | | 0.1174 | 0.2079 | 0.3547 | | 0.1761 | 0.3119 | 0.5321 | | 0.2347 | 0.4159 | 0.7095 | | 0.2934 | 0.5199 | 0.8868 | | 0.3521 | 0.6238 | 1.0642 | | 0.4108 | 0.7278 | 1.2416 | | 0.4695 | 0.8318 | 1.4189 | | 0.5282 | 0.9357 | 1.5963 | | 0.5868 | 1.0397 | 1.7737 | |
|  | **Cr** | 0.0468 | 0.0682 | 0.0883 | | 0.0936 | 0.1364 | 0.1766 | | 0.1404 | 0.2046 | 0.2649 | | 0.1871 | 0.2728 | 0.3532 | | 0.2339 | 0.3410 | 0.4415 | | 0.2807 | 0.4091 | 0.5298 | | 0.3275 | 0.4773 | 0.6181 | | 0.3743 | 0.5455 | 0.7064 | | 0.4211 | 0.6137 | 0.7947 | | 0.4678 | 0.6819 | 0.8830 | |
| **child**  **2-<3 years** | **Cd** | 0.0037 | 0.0138 | 0.0432 | | 0.0074 | 0.0276 | 0.0863 | | 0.0111 | 0.0413 | 0.1295 | | 0.0148 | 0.0551 | 0.1727 | | 0.0185 | 0.0689 | 0.2158 | | 0.0222 | 0.0827 | 0.2590 | | 0.0259 | 0.0964 | 0.3022 | | 0.0296 | 0.1102 | 0.3453 | | 0.0333 | 0.1240 | 0.3885 | | 0.0370 | 0.1378 | 0.4317 | |
|  | **Pb** | 0.0072 | 0.0284 | 0.1223 | | 0.0144 | 0.0568 | 0.2446 | | 0.0216 | 0.0852 | 0.3668 | | 0.0288 | 0.1136 | 0.4891 | | 0.0360 | 0.1420 | 0.6114 | | 0.0432 | 0.1704 | 0.7337 | | 0.0505 | 0.1988 | 0.8559 | | 0.0577 | 0.2272 | 0.9782 | | 0.0649 | 0.2556 | 1.1005 | | 0.0721 | 0.2840 | 1.2228 | |
|  | **Ni** | 0.0485 | 0.0859 | 0.1465 | | 0.0970 | 0.1718 | 0.2930 | | 0.1454 | 0.2577 | 0.4396 | | 0.1939 | 0.3436 | 0.5861 | | 0.2424 | 0.4294 | 0.7326 | | 0.2909 | 0.5153 | 0.8791 | | 0.3393 | 0.6012 | 1.0257 | | 0.3878 | 0.6871 | 1.1722 | | 0.4363 | 0.7730 | 1.3187 | | 0.4848 | 0.8589 | 1.4652 | |
|  | **Cr** | 0.0386 | 0.0563 | 0.0729 | | 0.0773 | 0.1127 | 0.1459 | | 0.1159 | 0.1690 | 0.2188 | | 0.1546 | 0.2253 | 0.2918 | | 0.1932 | 0.2817 | 0.3647 | | 0.2319 | 0.3380 | 0.4377 | | 0.2705 | 0.3943 | 0.5106 | | 0.3092 | 0.4507 | 0.5836 | | 0.3478 | 0.5070 | 0.6565 | | 0.3865 | 0.5633 | 0.7295 | |
| **child**  **3-<6 years** | **Cd** | 0.0027 | 0.0102 | 0.0320 | | 0.0055 | 0.0204 | 0.0641 | | 0.0082 | 0.0307 | 0.0961 | | 0.0110 | 0.0409 | 0.1281 | | 0.0137 | 0.0511 | 0.1601 | | 0.0165 | 0.0613 | 0.1922 | | 0.0192 | 0.0715 | 0.2242 | | 0.0220 | 0.0818 | 0.2562 | | 0.0247 | 0.0920 | 0.2882 | | 0.0275 | 0.1022 | 0.3203 | |
|  | **Pb** | 0.0053 | 0.0211 | 0.0907 | | 0.0107 | 0.0421 | 0.1814 | | 0.0160 | 0.0632 | 0.2722 | | 0.0214 | 0.0843 | 0.3629 | | 0.0267 | 0.1054 | 0.4536 | | 0.0321 | 0.1264 | 0.5443 | | 0.0374 | 0.1475 | 0.6350 | | 0.0428 | 0.1686 | 0.7258 | | 0.0481 | 0.1897 | 0.8165 | | 0.0535 | 0.2107 | 0.9072 | |
|  | **Ni** | 0.0360 | 0.0637 | 0.1087 | | 0.0719 | 0.1274 | 0.2174 | | 0.1079 | 0.1912 | 0.3261 | | 0.1439 | 0.2549 | 0.4348 | | 0.1798 | 0.3186 | 0.5435 | | 0.2158 | 0.3823 | 0.6523 | | 0.2518 | 0.4461 | 0.7610 | | 0.2877 | 0.5098 | 0.8697 | | 0.3237 | 0.5735 | 0.9784 | | 0.3597 | 0.6372 | 1.0871 | |
|  | **Cr** | 0.0287 | 0.0418 | 0.0541 | | 0.0573 | 0.0836 | 0.1082 | | 0.0860 | 0.1254 | 0.1624 | | 0.1147 | 0.1672 | 0.2165 | | 0.1434 | 0.2090 | 0.2706 | | 0.1720 | 0.2508 | 0.3247 | | 0.2007 | 0.2926 | 0.3789 | | 0.2294 | 0.3344 | 0.4330 | | 0.2581 | 0.3762 | 0.4871 | | 0.2867 | 0.4179 | 0.5412 | |
| **child**  **6-<11 years** | **Cd** | 0.0016 | 0.0060 | 0.0187 | | 0.0032 | 0.0120 | 0.0375 | | 0.0048 | 0.0179 | 0.0562 | | 0.0064 | 0.0239 | 0.0749 | | 0.0080 | 0.0299 | 0.0937 | | 0.0096 | 0.0359 | 0.1124 | | 0.0112 | 0.0418 | 0.1311 | | 0.0129 | 0.0478 | 0.1499 | | 0.0145 | 0.0538 | 0.1686 | | 0.0161 | 0.0598 | 0.1873 | |
|  | **Pb** | 0.0031 | 0.0123 | 0.0531 | | 0.0063 | 0.0247 | 0.1061 | | 0.0094 | 0.0370 | 0.1592 | | 0.0125 | 0.0493 | 0.2123 | | 0.0156 | 0.0616 | 0.2653 | | 0.0188 | 0.0740 | 0.3184 | | 0.0219 | 0.0863 | 0.3714 | | 0.0250 | 0.0986 | 0.4245 | | 0.0281 | 0.1109 | 0.4776 | | 0.0313 | 0.1233 | 0.5306 | |
|  | **Ni** | 0.0210 | 0.0373 | 0.0636 | | 0.0421 | 0.0745 | 0.1272 | | 0.0631 | 0.1118 | 0.1908 | | 0.0842 | 0.1491 | 0.2543 | | 0.1052 | 0.1864 | 0.3179 | | 0.1262 | 0.2236 | 0.3815 | | 0.1473 | 0.2609 | 0.4451 | | 0.1683 | 0.2982 | 0.5087 | | 0.1893 | 0.3355 | 0.5723 | | 0.2104 | 0.3727 | 0.6358 | |
|  | **Cr** | 0.0168 | 0.0244 | 0.0317 | | 0.0335 | 0.0489 | 0.0633 | | 0.0503 | 0.0733 | 0.0950 | | 0.0671 | 0.0978 | 0.1266 | | 0.0839 | 0.1222 | 0.1583 | | 0.1006 | 0.1467 | 0.1899 | | 0.1174 | 0.1711 | 0.2216 | | 0.1342 | 0.1956 | 0.2532 | | 0.1509 | 0.2200 | 0.2849 | | 0.1677 | 0.2445 | 0.3166 | |
| **child**  **11-<16 years** | **Cd** | 0.0009 | 0.0033 | 0.0105 | | 0.0018 | 0.0067 | 0.0210 | | 0.0027 | 0.0100 | 0.0315 | | 0.0036 | 0.0134 | 0.0420 | | 0.0045 | 0.0167 | 0.0524 | | 0.0054 | 0.0201 | 0.0629 | | 0.0063 | 0.0234 | 0.0734 | | 0.0072 | 0.0268 | 0.0839 | | 0.0081 | 0.0301 | 0.0944 | | 0.0090 | 0.0335 | 0.1049 | |
|  | **Pb** | 0.0018 | 0.0069 | 0.0297 | | 0.0035 | 0.0138 | 0.0594 | | 0.0053 | 0.0207 | 0.0891 | | 0.0070 | 0.0276 | 0.1188 | | 0.0088 | 0.0345 | 0.1485 | | 0.0105 | 0.0414 | 0.1782 | | 0.0123 | 0.0483 | 0.2080 | | 0.0140 | 0.0552 | 0.2377 | | 0.0158 | 0.0621 | 0.2674 | | 0.0175 | 0.0690 | 0.2971 | |
|  | **Ni** | 0.0118 | 0.0209 | 0.0356 | | 0.0236 | 0.0417 | 0.0712 | | 0.0353 | 0.0626 | 0.1068 | | 0.0471 | 0.0835 | 0.1424 | | 0.0589 | 0.1043 | 0.1780 | | 0.0707 | 0.1252 | 0.2136 | | 0.0824 | 0.1461 | 0.2492 | | 0.0942 | 0.1669 | 0.2848 | | 0.1060 | 0.1878 | 0.3204 | | 0.1178 | 0.2087 | 0.3560 | |
|  | **Cr** | 0.0009 | 0.0033 | 0.0105 | | 0.0018 | 0.0067 | 0.0210 | | 0.0027 | 0.0100 | 0.0315 | | 0.0036 | 0.0134 | 0.0420 | | 0.0045 | 0.0167 | 0.0524 | | 0.0054 | 0.0201 | 0.0629 | | 0.0063 | 0.0234 | 0.0734 | | 0.0072 | 0.0268 | 0.0839 | | 0.0081 | 0.0301 | 0.0944 | | 0.0090 | 0.0335 | 0.1049 | |
| **child**  **16-<21 years** | **Cd** | 0.0007 | 0.0027 | 0.0083 | | 0.0014 | 0.0053 | 0.0166 | | 0.0021 | 0.0080 | 0.0250 | | 0.0029 | 0.0106 | 0.0333 | | 0.0036 | 0.0133 | 0.0416 | | 0.0043 | 0.0159 | 0.0499 | | 0.0050 | 0.0186 | 0.0582 | | 0.0057 | 0.0212 | 0.0666 | | 0.0064 | 0.0239 | 0.0749 | | 0.0071 | 0.0266 | 0.0832 | |
|  | **Pb** | 0.0014 | 0.0055 | 0.0236 | | 0.0028 | 0.0109 | 0.0471 | | 0.0042 | 0.0164 | 0.0707 | | 0.0056 | 0.0219 | 0.0943 | | 0.0069 | 0.0274 | 0.1178 | | 0.0083 | 0.0328 | 0.1414 | | 0.0097 | 0.0383 | 0.1650 | | 0.0111 | 0.0438 | 0.1885 | | 0.0125 | 0.0493 | 0.2121 | | 0.0139 | 0.0547 | 0.2357 | |
|  | **Ni** | 0.0093 | 0.0166 | 0.0282 | | 0.0187 | 0.0331 | 0.0565 | | 0.0280 | 0.0497 | 0.0847 | | 0.0374 | 0.0662 | 0.1130 | | 0.0467 | 0.0828 | 0.1412 | | 0.0561 | 0.0993 | 0.1694 | | 0.0654 | 0.1159 | 0.1977 | | 0.0747 | 0.1324 | 0.2259 | | 0.0841 | 0.1490 | 0.2542 | | 0.0934 | 0.1655 | 0.2824 | |
|  | **Cr** | 0.0074 | 0.0109 | 0.0141 | | 0.0149 | 0.0217 | 0.0281 | | 0.0223 | 0.0326 | 0.0422 | | 0.0298 | 0.0434 | 0.0562 | | 0.0372 | 0.0543 | 0.0703 | | 0.0447 | 0.0651 | 0.0844 | | 0.0521 | 0.0760 | 0.0984 | | 0.0596 | 0.0869 | 0.1125 | | 0.0670 | 0.0977 | 0.1265 | | 0.0745 | 0.1086 | 0.1406 | |
| **adult** | **Cd** | 0.0006 | 0.0024 | 0.0074 | | 0.0013 | 0.0048 | 0.0149 | | 0.0019 | 0.0071 | 0.0223 | | 0.0026 | 0.0095 | 0.0298 | | 0.0032 | 0.0119 | 0.0372 | | 0.0038 | 0.0143 | 0.0447 | | 0.0045 | 0.0166 | 0.0521 | | 0.0051 | 0.0190 | 0.0596 | | 0.0057 | 0.0214 | 0.0670 | | 0.0064 | 0.0238 | 0.0745 | |
|  | **Pb** | 0.0012 | 0.0049 | 0.0211 | | 0.0025 | 0.0098 | 0.0422 | | 0.0037 | 0.0147 | 0.0633 | | 0.0050 | 0.0196 | 0.0844 | | 0.0062 | 0.0245 | 0.1055 | | 0.0075 | 0.0294 | 0.1266 | | 0.0087 | 0.0343 | 0.1476 | | 0.0099 | 0.0392 | 0.1687 | | 0.0112 | 0.0441 | 0.1898 | | 0.0124 | 0.0490 | 0.2109 | |
|  | **Ni** | 0.0084 | 0.0148 | 0.0253 | | 0.0167 | 0.0296 | 0.0506 | | 0.0251 | 0.0444 | 0.0758 | | 0.0335 | 0.0593 | 0.1011 | | 0.0418 | 0.0741 | 0.1264 | | 0.0502 | 0.0889 | 0.1517 | | 0.0585 | 0.1037 | 0.1769 | | 0.0669 | 0.1185 | 0.2022 | | 0.0753 | 0.1333 | 0.2275 | | 0.0836 | 0.1482 | 0.2528 | |
|  | **Cr** | 0.0067 | 0.0097 | 0.0126 | | 0.0133 | 0.0194 | 0.0252 | | 0.0200 | 0.0292 | 0.0378 | | 0.0267 | 0.0389 | 0.0503 | | 0.0333 | 0.0486 | 0.0629 | | 0.0400 | 0.0583 | 0.0755 | | 0.0467 | 0.0680 | 0.0881 | | 0.0533 | 0.0777 | 0.1007 | | 0.0600 | 0.0875 | 0.1133 | | 0.0667 | 0.0972 | 0.1258 | |

**Table C.4** Hazard Quotient (HQ) calculated for Cd, Pb, Ni, and Cr contained in walnuts

| **population** | **element** | **HQ > 1** | | | | | | | | | | | | | | | | | | | | | | | | | | | | | |
| --- | --- | --- | --- | --- | --- | --- | --- | --- | --- | --- | --- | --- | --- | --- | --- | --- | --- | --- | --- | --- | --- | --- | --- | --- | --- | --- | --- | --- | --- | --- | --- |
|  |  | **A** | | | **B** | | | **C** | | | **D** | | | **E** | | | **F** | | | **G** | | | **H** | | | **I** | | | **J** | | |
|  |  | **I** | **II** | **III** | **I** | **II** | **III** | **I** | **II** | **III** | **I** | **II** | **III** | **I** | **II** | **III** | **I** | **II** | **III** | **I** | **II** | **III** | **I** | **II** | **III** | **I** | **II** | **III** | **I** | **II** | **III** |
| **child**  **6-11 months** | **Cd** | 0.0073 | 0.0162 | 0.0256 | 0.0146 | 0.0324 | 0.0512 | 0.0219 | 0.0486 | 0.0767 | 0.0292 | 0.0648 | 0.1023 | 0.0365 | 0.0810 | 0.1279 | 0.0438 | 0.0972 | 0.1535 | 0.0511 | 0.1134 | 0.1790 | 0.0583 | 0.1297 | 0.2046 | 0.0656 | 0.1459 | 0.2302 | 0.0729 | 0.1621 | 0.2558 |
|  | **Pb** | 0.0111 | 0.0357 | 0.1195 | 0.0222 | 0.0714 | 0.2391 | 0.0333 | 0.1071 | 0.3586 | 0.0444 | 0.1428 | 0.4781 | 0.0555 | 0.1786 | 0.5976 | 0.0666 | 0.2143 | 0.7172 | 0.0777 | 0.2500 | 0.8367 | 0.0888 | 0.2857 | 0.9562 | 0.0999 | 0.3214 | 1.0758 | 0.1110 | 0.3571 | 1.1953 |
|  | **Ni** | 0.0416 | 0.1503 | 0.3079 | 0.0832 | 0.3005 | 0.6159 | 0.1247 | 0.4508 | 0.9238 | 0.1663 | 0.6011 | 1.2317 | 0.2079 | 0.7513 | 1.5397 | 0.2495 | 0.9016 | 1.8476 | 0.2910 | 1.0519 | 2.1555 | 0.3326 | 1.2021 | 2.4635 | 0.3742 | 1.3524 | 2.7714 | 0.4158 | 1.5027 | 3.0793 |
|  | **Cr** | 0.0319 | 0.0481 | 0.0710 | 0.0638 | 0.0962 | 0.1420 | 0.0957 | 0.1443 | 0.2130 | 0.1275 | 0.1924 | 0.2841 | 0.1594 | 0.2405 | 0.3551 | 0.1913 | 0.2886 | 0.4261 | 0.2232 | 0.3367 | 0.4971 | 0.2551 | 0.3848 | 0.5681 | 0.2870 | 0.4329 | 0.6391 | 0.3188 | 0.4810 | 0.7101 |
| **child**  **1-<2 years** | **Cd** | 0.0059 | 0.0131 | 0.0206 | 0.0118 | 0.0262 | 0.0413 | 0.0177 | 0.0392 | 0.0619 | 0.0235 | 0.0523 | 0.0826 | 0.0294 | 0.0654 | 0.1032 | 0.0353 | 0.0785 | 0.1238 | 0.0412 | 0.0916 | 0.1445 | 0.0471 | 0.1046 | 0.1651 | 0.0530 | 0.1177 | 0.1858 | 0.0589 | 0.1308 | 0.2064 |
|  | **Pb** | 0.0090 | 0.0288 | 0.0965 | 0.0179 | 0.0576 | 0.1929 | 0.0269 | 0.0865 | 0.2894 | 0.0358 | 0.1153 | 0.3858 | 0.0448 | 0.1441 | 0.4823 | 0.0537 | 0.1729 | 0.5788 | 0.0627 | 0.2017 | 0.6752 | 0.0716 | 0.2306 | 0.7717 | 0.0806 | 0.2594 | 0.8681 | 0.0896 | 0.2882 | 0.9646 |
|  | **Ni** | 0.0336 | 0.1213 | 0.2485 | 0.0671 | 0.2425 | 0.4970 | 0.1007 | 0.3638 | 0.7455 | 0.1342 | 0.4851 | 0.9940 | 0.1678 | 0.6063 | 1.2425 | 0.2013 | 0.7276 | 1.4911 | 0.2349 | 0.8489 | 1.7396 | 0.2684 | 0.9702 | 1.9881 | 0.3020 | 1.0914 | 2.2366 | 0.3355 | 1.2127 | 2.4851 |
|  | **Cr** | 0.0257 | 0.0388 | 0.0573 | 0.0515 | 0.0776 | 0.1146 | 0.0772 | 0.1165 | 0.1719 | 0.1029 | 0.1553 | 0.2292 | 0.1287 | 0.1941 | 0.2865 | 0.1544 | 0.2329 | 0.3439 | 0.1801 | 0.2717 | 0.4012 | 0.2058 | 0.3106 | 0.4585 | 0.2316 | 0.3494 | 0.5158 | 0.2573 | 0.3882 | 0.5731 |
| **child**  **2-<3 years** | **Cd** | 0.0049 | 0.0108 | 0.0171 | 0.0097 | 0.0216 | 0.0341 | 0.0146 | 0.0324 | 0.0512 | 0.0194 | 0.0432 | 0.0682 | 0.0243 | 0.0540 | 0.0853 | 0.0292 | 0.0648 | 0.1023 | 0.0340 | 0.0756 | 0.1194 | 0.0389 | 0.0864 | 0.1364 | 0.0438 | 0.0972 | 0.1535 | 0.0486 | 0.1080 | 0.1705 |
|  | **Pb** | 0.0074 | 0.0238 | 0.0797 | 0.0148 | 0.0476 | 0.1594 | 0.0222 | 0.0714 | 0.2391 | 0.0296 | 0.0952 | 0.3187 | 0.0370 | 0.1190 | 0.3984 | 0.0444 | 0.1428 | 0.4781 | 0.0518 | 0.1666 | 0.5578 | 0.0592 | 0.1905 | 0.6375 | 0.0666 | 0.2143 | 0.7172 | 0.0740 | 0.2381 | 0.7969 |
|  | **Ni** | 0.0277 | 0.1002 | 0.2053 | 0.0554 | 0.2004 | 0.4106 | 0.0832 | 0.3005 | 0.6159 | 0.1109 | 0.4007 | 0.8212 | 0.1386 | 0.5009 | 1.0264 | 0.1663 | 0.6011 | 1.2317 | 0.1940 | 0.7013 | 1.4370 | 0.2217 | 0.8014 | 1.6423 | 0.2495 | 0.9016 | 1.8476 | 0.2772 | 1.0018 | 2.0529 |
|  | **Cr** | 0.0213 | 0.0321 | 0.0473 | 0.0425 | 0.0641 | 0.0947 | 0.0638 | 0.0962 | 0.1420 | 0.0850 | 0.1283 | 0.1894 | 0.1063 | 0.1603 | 0.2367 | 0.1275 | 0.1924 | 0.2841 | 0.1488 | 0.2245 | 0.3314 | 0.1700 | 0.2566 | 0.3787 | 0.1913 | 0.2886 | 0.4261 | 0.2126 | 0.3207 | 0.4734 |
| **child**  **3-<6 years** | **Cd** | 0.0036 | 0.0080 | 0.0127 | 0.0072 | 0.0160 | 0.0253 | 0.0108 | 0.0240 | 0.0380 | 0.0144 | 0.0321 | 0.0506 | 0.0180 | 0.0401 | 0.0633 | 0.0216 | 0.0481 | 0.0759 | 0.0253 | 0.0561 | 0.0886 | 0.0289 | 0.0641 | 0.1012 | 0.0325 | 0.0721 | 0.1139 | 0.0361 | 0.0802 | 0.1265 |
|  | **Pb** | 0.0055 | 0.0177 | 0.0591 | 0.0110 | 0.0353 | 0.1182 | 0.0165 | 0.0530 | 0.1774 | 0.0220 | 0.0707 | 0.2365 | 0.0274 | 0.0883 | 0.2956 | 0.0329 | 0.1060 | 0.3547 | 0.0384 | 0.1236 | 0.4138 | 0.0439 | 0.1413 | 0.4730 | 0.0494 | 0.1590 | 0.5321 | 0.0549 | 0.1766 | 0.5912 |
|  | **Ni** | 0.0206 | 0.0743 | 0.1523 | 0.0411 | 0.1487 | 0.3046 | 0.0617 | 0.2230 | 0.4569 | 0.0823 | 0.2973 | 0.6092 | 0.1028 | 0.3716 | 0.7616 | 0.1234 | 0.4460 | 0.9139 | 0.1440 | 0.5203 | 1.0662 | 0.1645 | 0.5946 | 1.2185 | 0.1851 | 0.6689 | 1.3708 | 0.2056 | 0.7433 | 1.5231 |
|  | **Cr** | 0.0158 | 0.0238 | 0.0351 | 0.0315 | 0.0476 | 0.0703 | 0.0473 | 0.0714 | 0.1054 | 0.0631 | 0.0952 | 0.1405 | 0.0789 | 0.1190 | 0.1756 | 0.0946 | 0.1428 | 0.2108 | 0.1104 | 0.1666 | 0.2459 | 0.1262 | 0.1904 | 0.2810 | 0.1419 | 0.2141 | 0.3161 | 0.1577 | 0.2379 | 0.3513 |
| **child**  **6-<11 years** | **Cd** | 0.0021 | 0.0047 | 0.0074 | 0.0042 | 0.0094 | 0.0148 | 0.0063 | 0.0141 | 0.0222 | 0.0084 | 0.0188 | 0.0296 | 0.0106 | 0.0234 | 0.0370 | 0.0127 | 0.0281 | 0.0444 | 0.0148 | 0.0328 | 0.0518 | 0.0169 | 0.0375 | 0.0592 | 0.0190 | 0.0422 | 0.0666 | 0.0211 | 0.0469 | 0.0740 |
|  | **Pb** | 0.0032 | 0.0103 | 0.0346 | 0.0064 | 0.0207 | 0.0692 | 0.0096 | 0.0310 | 0.1037 | 0.0128 | 0.0413 | 0.1383 | 0.0161 | 0.0517 | 0.1729 | 0.0193 | 0.0620 | 0.2075 | 0.0225 | 0.0723 | 0.2421 | 0.0257 | 0.0827 | 0.2766 | 0.0289 | 0.0930 | 0.3112 | 0.0321 | 0.1033 | 0.3458 |
|  | **Ni** | 0.0120 | 0.0435 | 0.0891 | 0.0241 | 0.0869 | 0.1782 | 0.0361 | 0.1304 | 0.2673 | 0.0481 | 0.1739 | 0.3564 | 0.0601 | 0.2174 | 0.4454 | 0.0722 | 0.2608 | 0.5345 | 0.0842 | 0.3043 | 0.6236 | 0.0962 | 0.3478 | 0.7127 | 0.1083 | 0.3913 | 0.8018 | 0.1203 | 0.4347 | 0.8909 |
|  | **Cr** | 0.0092 | 0.0139 | 0.0205 | 0.0184 | 0.0278 | 0.0411 | 0.0277 | 0.0418 | 0.0616 | 0.0369 | 0.0557 | 0.0822 | 0.0461 | 0.0696 | 0.1027 | 0.0553 | 0.0835 | 0.1233 | 0.0646 | 0.0974 | 0.1438 | 0.0738 | 0.1113 | 0.1644 | 0.0830 | 0.1253 | 0.1849 | 0.0922 | 0.1392 | 0.2055 |
| **child**  **11-<16 years** | **Cd** | 0.0012 | 0.0026 | 0.0041 | 0.0024 | 0.0053 | 0.0083 | 0.0035 | 0.0079 | 0.0124 | 0.0047 | 0.0105 | 0.0166 | 0.0059 | 0.0131 | 0.0207 | 0.0071 | 0.0158 | 0.0249 | 0.0083 | 0.0184 | 0.0290 | 0.0095 | 0.0210 | 0.0331 | 0.0106 | 0.0236 | 0.0373 | 0.0118 | 0.0263 | 0.0414 |
|  | **Pb** | 0.0018 | 0.0058 | 0.0194 | 0.0036 | 0.0116 | 0.0387 | 0.0054 | 0.0174 | 0.0581 | 0.0072 | 0.0231 | 0.0774 | 0.0090 | 0.0289 | 0.0968 | 0.0108 | 0.0347 | 0.1162 | 0.0126 | 0.0405 | 0.1355 | 0.0144 | 0.0463 | 0.1549 | 0.0162 | 0.0521 | 0.1742 | 0.0180 | 0.0578 | 0.1936 |
|  | **Ni** | 0.0067 | 0.0243 | 0.0499 | 0.0135 | 0.0487 | 0.0998 | 0.0202 | 0.0730 | 0.1496 | 0.0269 | 0.0974 | 0.1995 | 0.0337 | 0.1217 | 0.2494 | 0.0404 | 0.1460 | 0.2993 | 0.0471 | 0.1704 | 0.3491 | 0.0539 | 0.1947 | 0.3990 | 0.0606 | 0.2191 | 0.4489 | 0.0673 | 0.2434 | 0.4988 |
|  | **Cr** | 0.0052 | 0.0078 | 0.0115 | 0.0103 | 0.0156 | 0.0230 | 0.0155 | 0.0234 | 0.0345 | 0.0207 | 0.0312 | 0.0460 | 0.0258 | 0.0390 | 0.0575 | 0.0310 | 0.0467 | 0.0690 | 0.0362 | 0.0545 | 0.0805 | 0.0413 | 0.0623 | 0.0920 | 0.0465 | 0.0701 | 0.1035 | 0.0516 | 0.0779 | 0.1150 |
| **child**  **16-<21 years** | **Cd** | 0.0009 | 0.0021 | 0.0033 | 0.0019 | 0.0042 | 0.0066 | 0.0028 | 0.0062 | 0.0099 | 0.0037 | 0.0083 | 0.0131 | 0.0047 | 0.0104 | 0.0164 | 0.0056 | 0.0125 | 0.0197 | 0.0066 | 0.0146 | 0.0230 | 0.0075 | 0.0167 | 0.0263 | 0.0084 | 0.0187 | 0.0296 | 0.0094 | 0.0208 | 0.0329 |
|  | **Pb** | 0.0014 | 0.0046 | 0.0154 | 0.0029 | 0.0092 | 0.0307 | 0.0043 | 0.0138 | 0.0461 | 0.0057 | 0.0184 | 0.0614 | 0.0071 | 0.0229 | 0.0768 | 0.0086 | 0.0275 | 0.0921 | 0.0100 | 0.0321 | 0.1075 | 0.0114 | 0.0367 | 0.1229 | 0.0128 | 0.0413 | 0.1382 | 0.0143 | 0.0459 | 0.1536 |
|  | **Ni** | 0.0053 | 0.0193 | 0.0396 | 0.0107 | 0.0386 | 0.0791 | 0.0160 | 0.0579 | 0.1187 | 0.0214 | 0.0772 | 0.1583 | 0.0267 | 0.0965 | 0.1978 | 0.0321 | 0.1158 | 0.2374 | 0.0374 | 0.1352 | 0.2770 | 0.0427 | 0.1545 | 0.3165 | 0.0481 | 0.1738 | 0.3561 | 0.0534 | 0.1931 | 0.3957 |
|  | **Cr** | 0.0041 | 0.0062 | 0.0091 | 0.0082 | 0.0124 | 0.0182 | 0.0123 | 0.0185 | 0.0274 | 0.0164 | 0.0247 | 0.0365 | 0.0205 | 0.0309 | 0.0456 | 0.0246 | 0.0371 | 0.0547 | 0.0287 | 0.0433 | 0.0639 | 0.0328 | 0.0494 | 0.0730 | 0.0369 | 0.0556 | 0.0821 | 0.0410 | 0.0618 | 0.0912 |
| **adult** | **Cd** | 0.0008 | 0.0019 | 0.0029 | 0.0017 | 0.0037 | 0.0059 | 0.0025 | 0.0056 | 0.0088 | 0.0034 | 0.0075 | 0.0118 | 0.0042 | 0.0093 | 0.0147 | 0.0050 | 0.0112 | 0.0176 | 0.0059 | 0.0130 | 0.0206 | 0.0067 | 0.0149 | 0.0235 | 0.0075 | 0.0168 | 0.0265 | 0.0084 | 0.0186 | 0.0294 |
|  | **Pb** | 0.0013 | 0.0041 | 0.0137 | 0.0026 | 0.0082 | 0.0275 | 0.0038 | 0.0123 | 0.0412 | 0.0051 | 0.0164 | 0.0550 | 0.0064 | 0.0205 | 0.0687 | 0.0077 | 0.0246 | 0.0825 | 0.0089 | 0.0287 | 0.0962 | 0.0102 | 0.0329 | 0.1100 | 0.0115 | 0.0370 | 0.1237 | 0.0128 | 0.0411 | 0.1375 |
|  | **Ni** | 0.0048 | 0.0173 | 0.0354 | 0.0096 | 0.0346 | 0.0708 | 0.0143 | 0.0518 | 0.1062 | 0.0191 | 0.0691 | 0.1417 | 0.0239 | 0.0864 | 0.1771 | 0.0287 | 0.1037 | 0.2125 | 0.0335 | 0.1210 | 0.2479 | 0.0383 | 0.1382 | 0.2833 | 0.0430 | 0.1555 | 0.3187 | 0.0478 | 0.1728 | 0.3541 |
|  | **Cr** | 0.0037 | 0.0055 | 0.0082 | 0.0073 | 0.0111 | 0.0163 | 0.0110 | 0.0166 | 0.0245 | 0.0147 | 0.0221 | 0.0327 | 0.0183 | 0.0277 | 0.0408 | 0.0220 | 0.0332 | 0.0490 | 0.0257 | 0.0387 | 0.0572 | 0.0293 | 0.0443 | 0.0653 | 0.0330 | 0.0498 | 0.0735 | 0.0367 | 0.0553 | 0.0817 |

**Table C.5** Hazard Quotient (HQ) calculated for Cd, Pb, Ni, and Cr contained in cashews

| **population** | **element** | **HQ > 1** | | | | | | | | | | | | | | | | | | | | | | | | | | | | | | | | | | |
| --- | --- | --- | --- | --- | --- | --- | --- | --- | --- | --- | --- | --- | --- | --- | --- | --- | --- | --- | --- | --- | --- | --- | --- | --- | --- | --- | --- | --- | --- | --- | --- | --- | --- | --- | --- | --- |
|  |  | **A** | | | **B** | | | | **C** | | | | **D** | | | | **E** | | | | **F** | | | | **G** | | | **H** | | | **I** | | | **J** | | |
|  |  | **I** | **II** | **III** | **I** | **II** | **III** | **I** | | **II** | **III** | **I** | | **II** | **III** | **I** | | **II** | **III** | **I** | | **II** | **III** | **I** | | **II** | **III** | **I** | **II** | **III** | **I** | **II** | **III** | **I** | **II** | **III** |
| **child**  **6-11 months** | **Cd** |  |  | 0.0699 |  |  | 0.1398 |  | |  | 0.2098 |  | |  | 0.2797 |  | |  | 0.3496 |  | |  | 0.4195 |  | |  | 0.4894 |  |  | 0.5593 |  |  | 0.6293 |  |  | 0.6992 |
|  | **Pb** |  |  | 0.1948 |  |  | 0.3896 |  | |  | 0.5844 |  | |  | 0.7792 |  | |  | 0.9740 |  | |  | 1.1688 |  | |  | 1.3636 |  |  | 1.5584 |  |  | 1.7532 |  |  | 1.9480 |
|  | **Ni** | 0.2372 | 0.3497 | 0.4337 | 0.4745 | 0.6994 | 0.8674 | 0.7117 | | 1.0490 | 1.3011 | 0.9489 | | 1.3987 | 1.7348 | 1.1861 | | 1.7484 | 2.1685 | 1.4234 | | 2.0981 | 2.6022 | 1.6606 | | 2.4477 | 3.0359 | 1.8978 | 2.7974 | 3.4696 | 2.1351 | 3.1471 | 3.9033 | 2.3723 | 3.4968 | 4.3370 |
|  | **Cr** | 0.0670 | 0.0844 | 0.1087 | 0.1341 | 0.1687 | 0.2174 | 0.2011 | | 0.2531 | 0.3261 | 0.2681 | | 0.3374 | 0.4348 | 0.3351 | | 0.4218 | 0.5435 | 0.4022 | | 0.5061 | 0.6522 | 0.4692 | | 0.5905 | 0.7609 | 0.5362 | 0.6748 | 0.8696 | 0.6033 | 0.7592 | 0.9783 | 0.6703 | 0.8435 | 1.0870 |
| **child**  **1-<2 years** | **Cd** |  |  | 0.0564 |  |  | 0.1129 |  | |  | 0.1693 |  | |  | 0.2257 |  | |  | 0.2821 |  | |  | 0.3386 |  | |  | 0.3950 |  |  | 0.4514 |  |  | 0.5078 |  |  | 0.5643 |
|  | **Pb** |  |  | 0.1572 |  |  | 0.3144 |  | |  | 0.4716 |  | |  | 0.6288 |  | |  | 0.7860 |  | |  | 0.9432 |  | |  | 1.1005 |  |  | 1.2577 |  |  | 1.4149 |  |  | 1.5721 |
|  | **Ni** | 0.1914 | 0.2822 | 0.3500 | 0.3829 | 0.5644 | 0.7000 | 0.5743 | | 0.8466 | 1.0500 | 0.7658 | | 1.1288 | 1.4000 | 0.9572 | | 1.4110 | 1.7500 | 1.1487 | | 1.6932 | 2.1000 | 1.3401 | | 1.9754 | 2.4500 | 1.5316 | 2.2576 | 2.8000 | 1.7230 | 2.5398 | 3.1500 | 1.9145 | 2.8220 | 3.5000 |
|  | **Cr** | 0.0541 | 0.0681 | 0.0877 | 0.1082 | 0.1361 | 0.1754 | 0.1623 | | 0.2042 | 0.2632 | 0.2164 | | 0.2723 | 0.3509 | 0.2705 | | 0.3404 | 0.4386 | 0.3246 | | 0.4084 | 0.5263 | 0.3787 | | 0.4765 | 0.6140 | 0.4327 | 0.5446 | 0.7018 | 0.4868 | 0.6127 | 0.7895 | 0.5409 | 0.6807 | 0.8772 |
| **child**  **2-<3 years** | **Cd** |  |  | 0.0466 |  |  | 0.0932 |  | |  | 0.1398 |  | |  | 0.1864 |  | |  | 0.2331 |  | |  | 0.2797 |  | |  | 0.3263 |  |  | 0.3729 |  |  | 0.4195 |  |  | 0.4661 |
|  | **Pb** |  |  | 0.1299 |  |  | 0.2597 |  | |  | 0.3896 |  | |  | 0.5195 |  | |  | 0.6493 |  | |  | 0.7792 |  | |  | 0.9091 |  |  | 1.0389 |  |  | 1.1688 |  |  | 1.2987 |
|  | **Ni** | 0.1582 | 0.2331 | 0.2891 | 0.3163 | 0.4662 | 0.5783 | 0.4745 | | 0.6994 | 0.8674 | 0.6326 | | 0.9325 | 1.1565 | 0.7908 | | 1.1656 | 1.4457 | 0.9489 | | 1.3987 | 1.7348 | 1.1071 | | 1.6318 | 2.0239 | 1.2652 | 1.8649 | 2.3130 | 1.4234 | 2.0981 | 2.6022 | 1.5815 | 2.3312 | 2.8913 |
|  | **Cr** | 0.0447 | 0.0562 | 0.0725 | 0.0894 | 0.1125 | 0.1449 | 0.1341 | | 0.1687 | 0.2174 | 0.1787 | | 0.2249 | 0.2899 | 0.2234 | | 0.2812 | 0.3623 | 0.2681 | | 0.3374 | 0.4348 | 0.3128 | | 0.3936 | 0.5072 | 0.3575 | 0.4499 | 0.5797 | 0.4022 | 0.5061 | 0.6522 | 0.4469 | 0.5623 | 0.7246 |
| **child**  **3-<6 years** | **Cd** |  |  | 0.0346 |  |  | 0.0692 |  | |  | 0.1038 |  | |  | 0.1383 |  | |  | 0.1729 |  | |  | 0.2075 |  | |  | 0.2421 |  |  | 0.2767 |  |  | 0.3113 |  |  | 0.3458 |
|  | **Pb** |  |  | 0.0964 |  |  | 0.1927 |  | |  | 0.2891 |  | |  | 0.3854 |  | |  | 0.4818 |  | |  | 0.5781 |  | |  | 0.6745 |  |  | 0.7708 |  |  | 0.8672 |  |  | 0.9635 |
|  | **Ni** | 0.1173 | 0.1730 | 0.2145 | 0.2347 | 0.3459 | 0.4290 | 0.3520 | | 0.5189 | 0.6435 | 0.4694 | | 0.6918 | 0.8581 | 0.5867 | | 0.8648 | 1.0726 | 0.7040 | | 1.0378 | 1.2871 | 0.8214 | | 1.2107 | 1.5016 | 0.9387 | 1.3837 | 1.7161 | 1.0560 | 1.5566 | 1.9306 | 1.1734 | 1.7296 | 2.1452 |
|  | **Cr** | 0.0332 | 0.0417 | 0.0538 | 0.0663 | 0.0834 | 0.1075 | 0.0995 | | 0.1252 | 0.1613 | 0.1326 | | 0.1669 | 0.2151 | 0.1658 | | 0.2086 | 0.2688 | 0.1989 | | 0.2503 | 0.3226 | 0.2321 | | 0.2921 | 0.3763 | 0.2652 | 0.3338 | 0.4301 | 0.2984 | 0.3755 | 0.4839 | 0.3315 | 0.4172 | 0.5376 |
| **child**  **6-<11 years** | **Cd** |  |  | 0.0202 |  |  | 0.0405 |  | |  | 0.0607 |  | |  | 0.0809 |  | |  | 0.1011 |  | |  | 0.1214 |  | |  | 0.1416 |  |  | 0.1618 |  |  | 0.1821 |  |  | 0.2023 |
|  | **Pb** |  |  | 0.0564 |  |  | 0.1127 |  | |  | 0.1691 |  | |  | 0.2254 |  | |  | 0.2818 |  | |  | 0.3381 |  | |  | 0.3945 |  |  | 0.4509 |  |  | 0.5072 |  |  | 0.5636 |
|  | **Ni** | 0.0686 | 0.1012 | 0.1255 | 0.1373 | 0.2023 | 0.2509 | 0.2059 | | 0.3035 | 0.3764 | 0.2745 | | 0.4047 | 0.5019 | 0.3432 | | 0.5058 | 0.6274 | 0.4118 | | 0.6070 | 0.7528 | 0.4804 | | 0.7082 | 0.8783 | 0.5491 | 0.8093 | 1.0038 | 0.6177 | 0.9105 | 1.1292 | 0.6863 | 1.0116 | 1.2547 |
|  | **Cr** | 0.0194 | 0.0244 | 0.0314 | 0.0388 | 0.0488 | 0.0629 | 0.0582 | | 0.0732 | 0.0943 | 0.0776 | | 0.0976 | 0.1258 | 0.0970 | | 0.1220 | 0.1572 | 0.1164 | | 0.1464 | 0.1887 | 0.1357 | | 0.1708 | 0.2201 | 0.1551 | 0.1952 | 0.2516 | 0.1745 | 0.2196 | 0.2830 | 0.1939 | 0.2440 | 0.3145 |
| **child**  **11-<16 years** | **Cd** |  |  | 0.0113 |  |  | 0.0226 |  | |  | 0.0340 |  | |  | 0.0453 |  | |  | 0.0566 |  | |  | 0.0679 |  | |  | 0.0793 |  |  | 0.0906 |  |  | 0.1019 |  |  | 0.1132 |
|  | **Pb** |  |  | 0.0316 |  |  | 0.0631 |  | |  | 0.0947 |  | |  | 0.1262 |  | |  | 0.1578 |  | |  | 0.1893 |  | |  | 0.2209 |  |  | 0.2524 |  |  | 0.2840 |  |  | 0.3155 |
|  | **Ni** | 0.0384 | 0.0566 | 0.0702 | 0.0768 | 0.1133 | 0.1405 | 0.1153 | | 0.1699 | 0.2107 | 0.1537 | | 0.2266 | 0.2810 | 0.1921 | | 0.2832 | 0.3512 | 0.2305 | | 0.3398 | 0.4215 | 0.2690 | | 0.3965 | 0.4917 | 0.3074 | 0.4531 | 0.5620 | 0.3458 | 0.5097 | 0.6322 | 0.3842 | 0.5664 | 0.7025 |
|  | **Cr** | 0.0109 | 0.0137 | 0.0176 | 0.0217 | 0.0273 | 0.0352 | 0.0326 | | 0.0410 | 0.0528 | 0.0434 | | 0.0547 | 0.0704 | 0.0543 | | 0.0683 | 0.0880 | 0.0651 | | 0.0820 | 0.1056 | 0.0760 | | 0.0956 | 0.1232 | 0.0869 | 0.1093 | 0.1408 | 0.0977 | 0.1230 | 0.1585 | 0.1086 | 0.1366 | 0.1761 |
| **child**  **16-<21 years** | **Cd** |  |  | 0.0090 |  |  | 0.0180 |  | |  | 0.0270 |  | |  | 0.0359 |  | |  | 0.0449 |  | |  | 0.0539 |  | |  | 0.0629 |  |  | 0.0719 |  |  | 0.0809 |  |  | 0.0898 |
|  | **Pb** |  |  | 0.0250 |  |  | 0.0501 |  | |  | 0.0751 |  | |  | 0.1001 |  | |  | 0.1252 |  | |  | 0.1502 |  | |  | 0.1752 |  |  | 0.2002 |  |  | 0.2253 |  |  | 0.2503 |
|  | **Ni** | 0.0305 | 0.0449 | 0.0557 | 0.0610 | 0.0899 | 0.1115 | 0.0914 | | 0.1348 | 0.1672 | 0.1219 | | 0.1797 | 0.2229 | 0.1524 | | 0.2247 | 0.2786 | 0.1829 | | 0.2696 | 0.3344 | 0.2134 | | 0.3145 | 0.3901 | 0.2439 | 0.3594 | 0.4458 | 0.2743 | 0.4044 | 0.5015 | 0.3048 | 0.4493 | 0.5573 |
|  | **Cr** | 0.0086 | 0.0108 | 0.0140 | 0.0172 | 0.0217 | 0.0279 | 0.0258 | | 0.0325 | 0.0419 | 0.0345 | | 0.0434 | 0.0559 | 0.0431 | | 0.0542 | 0.0698 | 0.0517 | | 0.0650 | 0.0838 | 0.0603 | | 0.0759 | 0.0978 | 0.0689 | 0.0867 | 0.1117 | 0.0775 | 0.0975 | 0.1257 | 0.0861 | 0.1084 | 0.1397 |
| **adult** | **Cd** |  |  | 0.0080 |  |  | 0.0161 |  | |  | 0.0241 |  | |  | 0.0322 |  | |  | 0.0402 |  | |  | 0.0482 |  | |  | 0.0563 |  |  | 0.0643 |  |  | 0.0724 |  |  | 0.0804 |
|  | **Pb** |  |  | 0.0224 |  |  | 0.0448 |  | |  | 0.0672 |  | |  | 0.0896 |  | |  | 0.1120 |  | |  | 0.1344 |  | |  | 0.1568 |  |  | 0.1792 |  |  | 0.2016 |  |  | 0.2240 |
|  | **Ni** | 0.0273 | 0.0402 | 0.0499 | 0.0546 | 0.0804 | 0.0998 | 0.0818 | | 0.1206 | 0.1496 | 0.1091 | | 0.1609 | 0.1995 | 0.1364 | | 0.2011 | 0.2494 | 0.1637 | | 0.2413 | 0.2993 | 0.1910 | | 0.2815 | 0.3491 | 0.2183 | 0.3217 | 0.3990 | 0.2455 | 0.3619 | 0.4489 | 0.2728 | 0.4021 | 0.4988 |
|  | **Cr** | 0.0077 | 0.0097 | 0.0125 | 0.0154 | 0.0194 | 0.0250 | 0.0231 | | 0.0291 | 0.0375 | 0.0308 | | 0.0388 | 0.0500 | 0.0385 | | 0.0485 | 0.0625 | 0.0463 | | 0.0582 | 0.0750 | 0.0540 | | 0.0679 | 0.0875 | 0.0617 | 0.0776 | 0.1000 | 0.0694 | 0.0873 | 0.1125 | 0.0771 | 0.0970 | 0.1250 |

**Table D.1** Hazard Index (HI) calculated for Cd, Pb, Ni, and Cr taking into account exposure to all elements and the simultaneous consumption of two tested nut types

| **Nut type** |  | **Hazard Index scenario** | | | | | | | | | | | | | | | | | | | | | | | | | | | | | |
| --- | --- | --- | --- | --- | --- | --- | --- | --- | --- | --- | --- | --- | --- | --- | --- | --- | --- | --- | --- | --- | --- | --- | --- | --- | --- | --- | --- | --- | --- | --- | --- |
|  |  | **A** | | | **B** | | | **C** | | | **D** | | | **E** | | | **F** | | | **G** | | | **H** | | | **I** | | | **J** | | |
|  | **Age** | **I** | **II** | **III** | **I** | **II** | **III** | **I** | **II** | **III** | **I** | **II** | **III** | **I** | **II** | **III** | **I** | **II** | **III** | **I** | **II** | **III** | **I** | **II** | **III** | **I** | **II** | **III** | **I** | **II** | **III** |
| **peanuts + almonds** | **6-11 months** | 0.28 | 0.67 | 1.75 | 0.55 | 1.34 | 3.49 | 0.83 | 2.02 | 5.24 | 1.11 | 2.69 | 6.99 | 1.39 | 3.36 | 8.73 | 1.66 | 4.03 | 10.48 | 1.94 | 4.70 | 12.23 | 2.22 | 5.37 | 13.97 | 2.50 | 6.05 | 15.72 | 2.77 | 6.72 | 17.47 |
|  | **1-<2 years** | 0.22 | 0.54 | 1.41 | 0.45 | 1.08 | 2.82 | 0.67 | 1.63 | 4.23 | 0.90 | 2.17 | 5.64 | 1.12 | 2.71 | 7.05 | 1.34 | 3.25 | 8.46 | 1.57 | 3.80 | 9.87 | 1.79 | 4.34 | 11.28 | 2.02 | 4.88 | 12.69 | 2.24 | 5.42 | 14.10 |
|  | **2-<3 years** | 0.18 | 0.45 | 1.16 | 0.37 | 0.90 | 2.33 | 0.55 | 1.34 | 3.49 | 0.74 | 1.79 | 4.66 | 0.92 | 2.24 | 5.82 | 1.11 | 2.69 | 6.99 | 1.29 | 3.14 | 8.15 | 1.48 | 3.58 | 9.31 | 1.66 | 4.03 | 10.48 | 1.85 | 4.48 | 11.64 |
|  | **3-<6 years** | 0.14 | 0.33 | 0.86 | 0.27 | 0.66 | 1.73 | 0.41 | 1.00 | 2.59 | 0.55 | 1.33 | 3.46 | 0.69 | 1.66 | 4.32 | 0.82 | 1.99 | 5.18 | 0.96 | 2.33 | 6.05 | 1.10 | 2.66 | 6.91 | 1.24 | 2.99 | 7.77 | 1.37 | 3.32 | 8.64 |
|  | **6-<11 years** | 0.08 | 0.19 | 0.51 | 0.16 | 0.39 | 1.01 | 0.24 | 0.58 | 1.52 | 0.32 | 0.78 | 2.02 | 0.40 | 0.97 | 2.53 | 0.48 | 1.17 | 3.03 | 0.56 | 1.36 | 3.54 | 0.64 | 1.55 | 4.04 | 0.72 | 1.75 | 4.55 | 0.80 | 1.94 | 5.05 |
|  | **11-<16 years** | 0.04 | 0.11 | 0.28 | 0.09 | 0.22 | 0.57 | 0.13 | 0.33 | 0.85 | 0.18 | 0.44 | 1.13 | 0.22 | 0.54 | 1.41 | 0.27 | 0.65 | 1.70 | 0.31 | 0.76 | 1.98 | 0.36 | 0.87 | 2.26 | 0.40 | 0.98 | 2.55 | 0.45 | 1.09 | 2.83 |
|  | **16-<21 years** | 0.04 | 0.09 | 0.22 | 0.07 | 0.17 | 0.45 | 0.11 | 0.26 | 0.67 | 0.14 | 0.35 | 0.90 | 0.18 | 0.43 | 1.12 | 0.21 | 0.52 | 1.35 | 0.25 | 0.60 | 1.57 | 0.29 | 0.69 | 1.80 | 0.32 | 0.78 | 2.02 | 0.36 | 0.86 | 2.24 |
|  | **adult** | 0.03 | 0.08 | 0.20 | 0.06 | 0.15 | 0.40 | 0.10 | 0.23 | 0.60 | 0.13 | 0.31 | 0.80 | 0.16 | 0.39 | 1.00 | 0.19 | 0.46 | 1.21 | 0.22 | 0.54 | 1.41 | 0.26 | 0.62 | 1.61 | 0.29 | 0.70 | 1.81 | 0.32 | 0.77 | 2.01 |
| **peanuts + cashews** | **6-11 months** | 0.45 | 0.85 | 1.97 | 0.90 | 1.70 | 3.93 | 1.35 | 2.55 | 5.90 | 1.80 | 3.40 | 7.86 | 2.25 | 4.25 | 9.83 | 2.70 | 5.10 | 11.79 | 3.15 | 5.95 | 13.76 | 3.60 | 6.80 | 15.72 | 4.05 | 7.65 | 17.69 | 4.51 | 8.50 | 19.65 |
|  | **1-<2 years** | 0.36 | 0.69 | 1.59 | 0.73 | 1.37 | 3.17 | 1.09 | 2.06 | 4.76 | 1.45 | 2.74 | 6.34 | 1.82 | 3.43 | 7.93 | 2.18 | 4.11 | 9.52 | 2.54 | 4.80 | 11.10 | 2.91 | 5.49 | 12.69 | 3.27 | 6.17 | 14.27 | 3.64 | 6.86 | 15.86 |
|  | **2-<3 years** | 0.30 | 0.57 | 1.31 | 0.60 | 1.13 | 2.62 | 0.90 | 1.70 | 3.93 | 1.20 | 2.27 | 5.24 | 1.50 | 2.83 | 6.55 | 1.80 | 3.40 | 7.86 | 2.10 | 3.97 | 9.17 | 2.40 | 4.53 | 10.48 | 2.70 | 5.10 | 11.79 | 3.00 | 5.66 | 13.10 |
|  | **3-<6 years** | 0.22 | 0.42 | 0.97 | 0.45 | 0.84 | 1.94 | 0.67 | 1.26 | 2.92 | 0.89 | 1.68 | 3.89 | 1.11 | 2.10 | 4.86 | 1.34 | 2.52 | 5.83 | 1.56 | 2.94 | 6.80 | 1.78 | 3.36 | 7.78 | 2.01 | 3.78 | 8.75 | 2.23 | 4.20 | 9.72 |
|  | **6-<11 years** | 0.13 | 0.25 | 0.57 | 0.26 | 0.49 | 1.14 | 0.39 | 0.74 | 1.71 | 0.52 | 0.98 | 2.27 | 0.65 | 1.23 | 2.84 | 0.78 | 1.47 | 3.41 | 0.91 | 1.72 | 3.98 | 1.04 | 1.97 | 4.55 | 1.17 | 2.21 | 5.12 | 1.30 | 2.46 | 5.69 |
|  | **11-<16 years** | 0.07 | 0.14 | 0.32 | 0.15 | 0.28 | 0.64 | 0.22 | 0.41 | 0.95 | 0.29 | 0.55 | 1.27 | 0.36 | 0.69 | 1.59 | 0.44 | 0.83 | 1.91 | 0.51 | 0.96 | 2.23 | 0.58 | 1.10 | 2.55 | 0.66 | 1.24 | 2.86 | 0.73 | 1.38 | 3.18 |
|  | **16-<21 years** | 0.06 | 0.11 | 0.25 | 0.12 | 0.22 | 0.50 | 0.17 | 0.33 | 0.76 | 0.23 | 0.44 | 1.01 | 0.29 | 0.55 | 1.26 | 0.35 | 0.66 | 1.51 | 0.41 | 0.76 | 1.77 | 0.46 | 0.87 | 2.02 | 0.52 | 0.98 | 2.27 | 0.58 | 1.09 | 2.52 |
|  | **adult** | 0.05 | 0.10 | 0.23 | 0.10 | 0.20 | 0.45 | 0.16 | 0.29 | 0.68 | 0.21 | 0.39 | 0.90 | 0.26 | 0.49 | 1.13 | 0.31 | 0.59 | 1.36 | 0.36 | 0.68 | 1.58 | 0.41 | 0.78 | 1.81 | 0.47 | 0.88 | 2.03 | 0.52 | 0.98 | 2.26 |
| **peanuts + hazelnuts** | **6-11 months** | 0.29 | 0.69 | 1.74 | 0.59 | 1.38 | 3.47 | 0.88 | 2.08 | 5.21 | 1.17 | 2.77 | 6.94 | 1.47 | 3.46 | 8.68 | 1.76 | 4.15 | 10.41 | 2.05 | 4.85 | 12.15 | 2.35 | 5.54 | 13.88 | 2.64 | 6.23 | 15.62 | 2.93 | 6.92 | 17.35 |
|  | **1-<2 years** | 0.24 | 0.56 | 1.40 | 0.47 | 1.12 | 2.80 | 0.71 | 1.68 | 4.20 | 0.95 | 2.23 | 5.60 | 1.18 | 2.79 | 7.00 | 1.42 | 3.35 | 8.40 | 1.66 | 3.91 | 9.80 | 1.89 | 4.47 | 11.20 | 2.13 | 5.03 | 12.60 | 2.37 | 5.59 | 14.00 |
|  | **2-<3 years** | 0.20 | 0.46 | 1.16 | 0.39 | 0.92 | 2.31 | 0.59 | 1.38 | 3.47 | 0.78 | 1.85 | 4.63 | 0.98 | 2.31 | 5.78 | 1.17 | 2.77 | 6.94 | 1.37 | 3.23 | 8.10 | 1.56 | 3.69 | 9.26 | 1.76 | 4.15 | 10.41 | 1.96 | 4.62 | 11.57 |
|  | **3-<6 years** | 0.15 | 0.34 | 0.86 | 0.29 | 0.68 | 1.72 | 0.44 | 1.03 | 2.58 | 0.58 | 1.37 | 3.43 | 0.73 | 1.71 | 4.29 | 0.87 | 2.05 | 5.15 | 1.02 | 2.40 | 6.01 | 1.16 | 2.74 | 6.87 | 1.31 | 3.08 | 7.73 | 1.45 | 3.42 | 8.58 |
|  | **6-<11 years** | 0.08 | 0.20 | 0.50 | 0.17 | 0.40 | 1.00 | 0.25 | 0.60 | 1.51 | 0.34 | 0.80 | 2.01 | 0.42 | 1.00 | 2.51 | 0.51 | 1.20 | 3.01 | 0.59 | 1.40 | 3.51 | 0.68 | 1.60 | 4.02 | 0.76 | 1.80 | 4.52 | 0.85 | 2.00 | 5.02 |
|  | **11-<16 years** | 0.05 | 0.11 | 0.28 | 0.10 | 0.22 | 0.56 | 0.14 | 0.34 | 0.84 | 0.19 | 0.45 | 1.12 | 0.24 | 0.56 | 1.41 | 0.29 | 0.67 | 1.69 | 0.33 | 0.78 | 1.97 | 0.38 | 0.90 | 2.25 | 0.43 | 1.01 | 2.53 | 0.48 | 1.12 | 2.81 |
|  | **16-<21 years** | 0.04 | 0.09 | 0.22 | 0.08 | 0.18 | 0.45 | 0.11 | 0.27 | 0.67 | 0.15 | 0.36 | 0.89 | 0.19 | 0.44 | 1.11 | 0.23 | 0.53 | 1.34 | 0.26 | 0.62 | 1.56 | 0.30 | 0.71 | 1.78 | 0.34 | 0.80 | 2.01 | 0.38 | 0.89 | 2.23 |
|  | **adult** | 0.03 | 0.08 | 0.20 | 0.07 | 0.16 | 0.40 | 0.10 | 0.24 | 0.60 | 0.13 | 0.32 | 0.80 | 0.17 | 0.40 | 1.00 | 0.20 | 0.48 | 1.20 | 0.24 | 0.56 | 1.40 | 0.27 | 0.64 | 1.60 | 0.30 | 0.72 | 1.80 | 0.34 | 0.80 | 2.00 |
| **peanuts + walnuts** | **6-11 months** | 0.24 | 0.67 | 1.68 | 0.48 | 1.33 | 3.36 | 0.71 | 2.00 | 5.05 | 0.95 | 2.66 | 6.73 | 1.19 | 3.33 | 8.41 | 1.43 | 4.00 | 10.09 | 1.67 | 4.66 | 11.77 | 1.90 | 5.33 | 13.46 | 2.14 | 5.99 | 15.14 | 2.38 | 6.66 | 16.82 |
|  | **1-<2 years** | 0.19 | 0.54 | 1.36 | 0.38 | 1.07 | 2.71 | 0.58 | 1.61 | 4.07 | 0.77 | 2.15 | 5.43 | 0.96 | 2.69 | 6.79 | 1.15 | 3.22 | 8.14 | 1.35 | 3.76 | 9.50 | 1.54 | 4.30 | 10.86 | 1.73 | 4.84 | 12.22 | 1.92 | 5.37 | 13.57 |
|  | **2-<3 years** | 0.16 | 0.44 | 1.12 | 0.32 | 0.89 | 2.24 | 0.48 | 1.33 | 3.36 | 0.63 | 1.78 | 4.49 | 0.79 | 2.22 | 5.61 | 0.95 | 2.66 | 6.73 | 1.11 | 3.11 | 7.85 | 1.27 | 3.55 | 8.97 | 1.43 | 4.00 | 10.09 | 1.59 | 4.44 | 11.21 |
|  | **3-<6 years** | 0.12 | 0.33 | 0.83 | 0.24 | 0.66 | 1.66 | 0.35 | 0.99 | 2.50 | 0.47 | 1.32 | 3.33 | 0.59 | 1.65 | 4.16 | 0.71 | 1.98 | 4.99 | 0.82 | 2.31 | 5.82 | 0.94 | 2.64 | 6.66 | 1.06 | 2.96 | 7.49 | 1.18 | 3.29 | 8.32 |
|  | **6-<11 years** | 0.07 | 0.19 | 0.49 | 0.14 | 0.39 | 0.97 | 0.21 | 0.58 | 1.46 | 0.28 | 0.77 | 1.95 | 0.34 | 0.96 | 2.43 | 0.41 | 1.16 | 2.92 | 0.48 | 1.35 | 3.41 | 0.55 | 1.54 | 3.89 | 0.62 | 1.73 | 4.38 | 0.69 | 1.93 | 4.87 |
|  | **11-<16 years** | 0.04 | 0.11 | 0.27 | 0.08 | 0.22 | 0.54 | 0.12 | 0.32 | 0.82 | 0.15 | 0.43 | 1.09 | 0.19 | 0.54 | 1.36 | 0.23 | 0.65 | 1.63 | 0.27 | 0.76 | 1.91 | 0.31 | 0.86 | 2.18 | 0.35 | 0.97 | 2.45 | 0.39 | 1.08 | 2.72 |
|  | **16-<21 years** | 0.03 | 0.09 | 0.22 | 0.06 | 0.17 | 0.43 | 0.09 | 0.26 | 0.65 | 0.12 | 0.34 | 0.86 | 0.15 | 0.43 | 1.08 | 0.18 | 0.51 | 1.30 | 0.21 | 0.60 | 1.51 | 0.24 | 0.68 | 1.73 | 0.28 | 0.77 | 1.95 | 0.31 | 0.86 | 2.16 |
|  | **adult** | 0.03 | 0.08 | 0.19 | 0.05 | 0.15 | 0.39 | 0.08 | 0.23 | 0.58 | 0.11 | 0.31 | 0.77 | 0.14 | 0.38 | 0.97 | 0.16 | 0.46 | 1.16 | 0.19 | 0.54 | 1.35 | 0.22 | 0.61 | 1.55 | 0.25 | 0.69 | 1.74 | 0.27 | 0.77 | 1.93 |
| **almonds + cashews** | **6-11 months** | 0.44 | 0.69 | 1.40 | 0.87 | 1.38 | 2.79 | 1.31 | 2.07 | 4.19 | 1.74 | 2.76 | 5.58 | 2.18 | 3.45 | 6.98 | 2.61 | 4.14 | 8.37 | 3.05 | 4.83 | 9.77 | 3.48 | 5.52 | 11.17 | 3.92 | 6.21 | 12.56 | 4.35 | 6.90 | 13.96 |
|  | **1-<2 years** | 0.35 | 0.56 | 1.13 | 0.70 | 1.11 | 2.25 | 1.05 | 1.67 | 3.38 | 1.41 | 2.23 | 4.51 | 1.76 | 2.78 | 5.63 | 2.11 | 3.34 | 6.76 | 2.46 | 3.90 | 7.88 | 2.81 | 4.46 | 9.01 | 3.16 | 5.01 | 10.14 | 3.51 | 5.57 | 11.26 |
|  | **2-<3 years** | 0.29 | 0.46 | 0.93 | 0.58 | 0.92 | 1.86 | 0.87 | 1.38 | 2.79 | 1.16 | 1.84 | 3.72 | 1.45 | 2.30 | 4.65 | 1.74 | 2.76 | 5.58 | 2.03 | 3.22 | 6.51 | 2.32 | 3.68 | 7.44 | 2.61 | 4.14 | 8.37 | 2.90 | 4.60 | 9.30 |
|  | **3-<6 years** | 0.22 | 0.34 | 0.69 | 0.43 | 0.68 | 1.38 | 0.65 | 1.02 | 2.07 | 0.86 | 1.37 | 2.76 | 1.08 | 1.71 | 3.45 | 1.29 | 2.05 | 4.14 | 1.51 | 2.39 | 4.83 | 1.72 | 2.73 | 5.52 | 1.94 | 3.07 | 6.21 | 2.15 | 3.41 | 6.90 |
|  | **6-<11 years** | 0.13 | 0.20 | 0.40 | 0.25 | 0.40 | 0.81 | 0.38 | 0.60 | 1.21 | 0.50 | 0.80 | 1.62 | 0.63 | 1.00 | 2.02 | 0.76 | 1.20 | 2.42 | 0.88 | 1.40 | 2.83 | 1.01 | 1.60 | 3.23 | 1.13 | 1.80 | 3.63 | 1.26 | 2.00 | 4.04 |
|  | **11-<16 years** | 0.07 | 0.11 | 0.23 | 0.14 | 0.22 | 0.45 | 0.21 | 0.34 | 0.68 | 0.28 | 0.45 | 0.90 | 0.35 | 0.56 | 1.13 | 0.42 | 0.67 | 1.36 | 0.49 | 0.78 | 1.58 | 0.56 | 0.89 | 1.81 | 0.63 | 1.01 | 2.03 | 0.71 | 1.12 | 2.26 |
|  | **16-<21 years** | 0.06 | 0.09 | 0.18 | 0.11 | 0.18 | 0.36 | 0.17 | 0.27 | 0.54 | 0.22 | 0.35 | 0.72 | 0.28 | 0.44 | 0.90 | 0.34 | 0.53 | 1.08 | 0.39 | 0.62 | 1.26 | 0.45 | 0.71 | 1.43 | 0.50 | 0.80 | 1.61 | 0.56 | 0.89 | 1.79 |
|  | **adult** | 0.05 | 0.08 | 0.16 | 0.10 | 0.16 | 0.32 | 0.15 | 0.24 | 0.48 | 0.20 | 0.32 | 0.64 | 0.25 | 0.40 | 0.80 | 0.30 | 0.48 | 0.96 | 0.35 | 0.56 | 1.12 | 0.40 | 0.63 | 1.28 | 0.45 | 0.71 | 1.44 | 0.50 | 0.79 | 1.61 |
| **almonds + hazelnuts** | **6-11 months** | 0.28 | 0.53 | 1.17 | 0.56 | 1.07 | 2.33 | 0.83 | 1.60 | 3.50 | 1.11 | 2.13 | 4.66 | 1.39 | 2.66 | 5.83 | 1.67 | 3.20 | 7.00 | 1.95 | 3.73 | 8.16 | 2.23 | 4.26 | 9.33 | 2.50 | 4.79 | 10.49 | 2.78 | 5.33 | 11.66 |
|  | **1-<2 years** | 0.22 | 0.43 | 0.94 | 0.45 | 0.86 | 1.88 | 0.67 | 1.29 | 2.82 | 0.90 | 1.72 | 3.76 | 1.12 | 2.15 | 4.70 | 1.35 | 2.58 | 5.65 | 1.57 | 3.01 | 6.59 | 1.80 | 3.44 | 7.53 | 2.02 | 3.87 | 8.47 | 2.25 | 4.30 | 9.41 |
|  | **2-<3 years** | 0.19 | 0.36 | 0.78 | 0.37 | 0.71 | 1.55 | 0.56 | 1.07 | 2.33 | 0.74 | 1.42 | 3.11 | 0.93 | 1.78 | 3.89 | 1.11 | 2.13 | 4.66 | 1.30 | 2.49 | 5.44 | 1.48 | 2.84 | 6.22 | 1.67 | 3.20 | 7.00 | 1.86 | 3.55 | 7.77 |
|  | **3-<6 years** | 0.14 | 0.26 | 0.58 | 0.28 | 0.53 | 1.15 | 0.41 | 0.79 | 1.73 | 0.55 | 1.05 | 2.31 | 0.69 | 1.32 | 2.88 | 0.83 | 1.58 | 3.46 | 0.96 | 1.84 | 4.04 | 1.10 | 2.11 | 4.61 | 1.24 | 2.37 | 5.19 | 1.38 | 2.64 | 5.77 |
|  | **6-<11 years** | 0.08 | 0.15 | 0.34 | 0.16 | 0.31 | 0.67 | 0.24 | 0.46 | 1.01 | 0.32 | 0.62 | 1.35 | 0.40 | 0.77 | 1.69 | 0.48 | 0.92 | 2.02 | 0.56 | 1.08 | 2.36 | 0.64 | 1.23 | 2.70 | 0.72 | 1.39 | 3.04 | 0.81 | 1.54 | 3.37 |
|  | **11-<16 years** | 0.05 | 0.09 | 0.19 | 0.09 | 0.17 | 0.38 | 0.14 | 0.26 | 0.57 | 0.18 | 0.35 | 0.76 | 0.23 | 0.43 | 0.94 | 0.27 | 0.52 | 1.13 | 0.32 | 0.60 | 1.32 | 0.36 | 0.69 | 1.51 | 0.41 | 0.78 | 1.70 | 0.45 | 0.86 | 1.89 |
|  | **16-<21 years** | 0.04 | 0.07 | 0.15 | 0.07 | 0.14 | 0.30 | 0.11 | 0.21 | 0.45 | 0.14 | 0.27 | 0.60 | 0.18 | 0.34 | 0.75 | 0.21 | 0.41 | 0.90 | 0.25 | 0.48 | 1.05 | 0.29 | 0.55 | 1.20 | 0.32 | 0.62 | 1.35 | 0.36 | 0.68 | 1.50 |
|  | **adult** | 0.03 | 0.06 | 0.13 | 0.06 | 0.12 | 0.27 | 0.10 | 0.18 | 0.40 | 0.13 | 0.25 | 0.54 | 0.16 | 0.31 | 0.67 | 0.19 | 0.37 | 0.80 | 0.22 | 0.43 | 0.94 | 0.26 | 0.49 | 1.07 | 0.29 | 0.55 | 1.21 | 0.32 | 0.61 | 1.34 |
| **almonds + walnuts** | **6-11 months** | 0.22 | 0.51 | 1.11 | 0.45 | 1.01 | 2.23 | 0.67 | 1.52 | 3.34 | 0.89 | 2.03 | 4.45 | 1.12 | 2.53 | 5.56 | 1.34 | 3.04 | 6.68 | 1.56 | 3.55 | 7.79 | 1.78 | 4.05 | 8.90 | 2.01 | 4.56 | 10.01 | 2.23 | 5.06 | 11.13 |
|  | **1-<2 years** | 0.18 | 0.41 | 0.90 | 0.36 | 0.82 | 1.80 | 0.54 | 1.23 | 2.69 | 0.72 | 1.63 | 3.59 | 0.90 | 2.04 | 4.49 | 1.08 | 2.45 | 5.39 | 1.26 | 2.86 | 6.29 | 1.44 | 3.27 | 7.18 | 1.62 | 3.68 | 8.08 | 1.80 | 4.09 | 8.98 |
|  | **2-<3 years** | 0.15 | 0.34 | 0.74 | 0.30 | 0.68 | 1.48 | 0.45 | 1.01 | 2.23 | 0.59 | 1.35 | 2.97 | 0.74 | 1.69 | 3.71 | 0.89 | 2.03 | 4.45 | 1.04 | 2.36 | 5.19 | 1.19 | 2.70 | 5.93 | 1.34 | 3.04 | 6.68 | 1.49 | 3.38 | 7.42 |
|  | **3-<6 years** | 0.11 | 0.25 | 0.55 | 0.22 | 0.50 | 1.10 | 0.33 | 0.75 | 1.65 | 0.44 | 1.00 | 2.20 | 0.55 | 1.25 | 2.75 | 0.66 | 1.50 | 3.30 | 0.77 | 1.75 | 3.85 | 0.88 | 2.00 | 4.40 | 0.99 | 2.25 | 4.95 | 1.10 | 2.51 | 5.50 |
|  | **6-<11 years** | 0.06 | 0.15 | 0.32 | 0.13 | 0.29 | 0.64 | 0.19 | 0.44 | 0.97 | 0.26 | 0.59 | 1.29 | 0.32 | 0.73 | 1.61 | 0.39 | 0.88 | 1.93 | 0.45 | 1.03 | 2.25 | 0.52 | 1.17 | 2.58 | 0.58 | 1.32 | 2.90 | 0.65 | 1.47 | 3.22 |
|  | **11-<16 years** | 0.04 | 0.08 | 0.18 | 0.07 | 0.16 | 0.36 | 0.11 | 0.25 | 0.54 | 0.14 | 0.33 | 0.72 | 0.18 | 0.41 | 0.90 | 0.22 | 0.49 | 1.08 | 0.25 | 0.57 | 1.26 | 0.29 | 0.66 | 1.44 | 0.33 | 0.74 | 1.62 | 0.36 | 0.82 | 1.80 |
|  | **16-<21 years** | 0.03 | 0.07 | 0.14 | 0.06 | 0.13 | 0.29 | 0.09 | 0.20 | 0.43 | 0.11 | 0.26 | 0.57 | 0.14 | 0.33 | 0.71 | 0.17 | 0.39 | 0.86 | 0.20 | 0.46 | 1.00 | 0.23 | 0.52 | 1.14 | 0.26 | 0.59 | 1.29 | 0.29 | 0.65 | 1.43 |
|  | **adult** | 0.03 | 0.06 | 0.13 | 0.05 | 0.12 | 0.26 | 0.08 | 0.17 | 0.38 | 0.10 | 0.23 | 0.51 | 0.13 | 0.29 | 0.64 | 0.15 | 0.35 | 0.77 | 0.18 | 0.41 | 0.90 | 0.21 | 0.47 | 1.02 | 0.23 | 0.52 | 1.15 | 0.26 | 0.58 | 1.28 |
| **cashews + hazelnuts** | **6-11 months** | 0.45 | 0.71 | 1.38 | 0.90 | 1.42 | 2.77 | 1.35 | 2.13 | 4.15 | 1.81 | 2.84 | 5.54 | 2.26 | 3.55 | 6.92 | 2.71 | 4.26 | 8.31 | 3.16 | 4.97 | 9.69 | 3.61 | 5.69 | 11.08 | 4.06 | 6.40 | 12.46 | 4.51 | 7.11 | 13.84 |
|  | **1-<2 years** | 0.36 | 0.57 | 1.12 | 0.73 | 1.15 | 2.23 | 1.09 | 1.72 | 3.35 | 1.46 | 2.29 | 4.47 | 1.82 | 2.87 | 5.59 | 2.19 | 3.44 | 6.70 | 2.55 | 4.01 | 7.82 | 2.91 | 4.59 | 8.94 | 3.28 | 5.16 | 10.06 | 3.64 | 5.73 | 11.17 |
|  | **2-<3 years** | 0.30 | 0.47 | 0.92 | 0.60 | 0.95 | 1.85 | 0.90 | 1.42 | 2.77 | 1.20 | 1.90 | 3.69 | 1.50 | 2.37 | 4.61 | 1.81 | 2.84 | 5.54 | 2.11 | 3.32 | 6.46 | 2.41 | 3.79 | 7.38 | 2.71 | 4.26 | 8.31 | 3.01 | 4.74 | 9.23 |
|  | **3-<6 years** | 0.22 | 0.35 | 0.68 | 0.45 | 0.70 | 1.37 | 0.67 | 1.05 | 2.05 | 0.89 | 1.41 | 2.74 | 1.12 | 1.76 | 3.42 | 1.34 | 2.11 | 4.11 | 1.56 | 2.46 | 4.79 | 1.79 | 2.81 | 5.48 | 2.01 | 3.16 | 6.16 | 2.23 | 3.51 | 6.85 |
|  | **6-<11 years** | 0.13 | 0.21 | 0.40 | 0.26 | 0.41 | 0.80 | 0.39 | 0.62 | 1.20 | 0.52 | 0.82 | 1.60 | 0.65 | 1.03 | 2.00 | 0.78 | 1.23 | 2.40 | 0.91 | 1.44 | 2.80 | 1.04 | 1.64 | 3.20 | 1.18 | 1.85 | 3.60 | 1.31 | 2.06 | 4.01 |
|  | **11-<16 years** | 0.07 | 0.12 | 0.22 | 0.15 | 0.23 | 0.45 | 0.22 | 0.35 | 0.67 | 0.29 | 0.46 | 0.90 | 0.37 | 0.58 | 1.12 | 0.44 | 0.69 | 1.35 | 0.51 | 0.81 | 1.57 | 0.58 | 0.92 | 1.79 | 0.66 | 1.04 | 2.02 | 0.73 | 1.15 | 2.24 |
|  | **16-<21 years** | 0.06 | 0.09 | 0.18 | 0.12 | 0.18 | 0.36 | 0.17 | 0.27 | 0.53 | 0.23 | 0.37 | 0.71 | 0.29 | 0.46 | 0.89 | 0.35 | 0.55 | 1.07 | 0.41 | 0.64 | 1.25 | 0.46 | 0.73 | 1.42 | 0.52 | 0.82 | 1.60 | 0.58 | 0.91 | 1.78 |
|  | **adult** | 0.05 | 0.08 | 0.16 | 0.10 | 0.16 | 0.32 | 0.16 | 0.25 | 0.48 | 0.21 | 0.33 | 0.64 | 0.26 | 0.41 | 0.80 | 0.31 | 0.49 | 0.96 | 0.36 | 0.57 | 1.11 | 0.42 | 0.65 | 1.27 | 0.47 | 0.74 | 1.43 | 0.52 | 0.82 | 1.59 |
| **cashews + walnuts** | **6-11 months** | 0.40 | 0.68 | 1.33 | 0.79 | 1.37 | 2.66 | 1.19 | 2.05 | 3.99 | 1.58 | 2.74 | 5.32 | 1.98 | 3.42 | 6.66 | 2.38 | 4.11 | 7.99 | 2.77 | 4.79 | 9.32 | 3.17 | 5.47 | 10.65 | 3.56 | 6.16 | 11.98 | 3.96 | 6.84 | 13.31 |
|  | **1-<2 years** | 0.32 | 0.55 | 1.07 | 0.64 | 1.10 | 2.15 | 0.96 | 1.66 | 3.22 | 1.28 | 2.21 | 4.30 | 1.60 | 2.76 | 5.37 | 1.92 | 3.31 | 6.45 | 2.24 | 3.87 | 7.52 | 2.56 | 4.42 | 8.59 | 2.88 | 4.97 | 9.67 | 3.20 | 5.52 | 10.74 |
|  | **2-<3 years** | 0.26 | 0.46 | 0.89 | 0.53 | 0.91 | 1.77 | 0.79 | 1.37 | 2.66 | 1.06 | 1.82 | 3.55 | 1.32 | 2.28 | 4.44 | 1.58 | 2.74 | 5.32 | 1.85 | 3.19 | 6.21 | 2.11 | 3.65 | 7.10 | 2.38 | 4.11 | 7.99 | 2.64 | 4.56 | 8.87 |
|  | **3-<6 years** | 0.20 | 0.34 | 0.66 | 0.39 | 0.68 | 1.32 | 0.59 | 1.02 | 1.98 | 0.78 | 1.35 | 2.63 | 0.98 | 1.69 | 3.29 | 1.18 | 2.03 | 3.95 | 1.37 | 2.37 | 4.61 | 1.57 | 2.71 | 5.27 | 1.76 | 3.05 | 5.93 | 1.96 | 3.38 | 6.58 |
|  | **6-<11 years** | 0.11 | 0.20 | 0.39 | 0.23 | 0.40 | 0.77 | 0.34 | 0.59 | 1.16 | 0.46 | 0.79 | 1.54 | 0.57 | 0.99 | 1.93 | 0.69 | 1.19 | 2.31 | 0.80 | 1.39 | 2.70 | 0.92 | 1.58 | 3.08 | 1.03 | 1.78 | 3.47 | 1.15 | 1.98 | 3.85 |
|  | **11-<16 years** | 0.06 | 0.11 | 0.22 | 0.13 | 0.22 | 0.43 | 0.19 | 0.33 | 0.65 | 0.26 | 0.44 | 0.86 | 0.32 | 0.55 | 1.08 | 0.38 | 0.67 | 1.29 | 0.45 | 0.78 | 1.51 | 0.51 | 0.89 | 1.72 | 0.58 | 1.00 | 1.94 | 0.64 | 1.11 | 2.16 |
|  | **16-<21 years** | 0.05 | 0.09 | 0.17 | 0.10 | 0.18 | 0.34 | 0.15 | 0.26 | 0.51 | 0.20 | 0.35 | 0.68 | 0.25 | 0.44 | 0.86 | 0.31 | 0.53 | 1.03 | 0.36 | 0.62 | 1.20 | 0.41 | 0.70 | 1.37 | 0.46 | 0.79 | 1.54 | 0.51 | 0.88 | 1.71 |
|  | **adult** | 0.05 | 0.08 | 0.15 | 0.09 | 0.16 | 0.31 | 0.14 | 0.24 | 0.46 | 0.18 | 0.31 | 0.61 | 0.23 | 0.39 | 0.77 | 0.27 | 0.47 | 0.92 | 0.32 | 0.55 | 1.07 | 0.36 | 0.63 | 1.22 | 0.41 | 0.71 | 1.38 | 0.46 | 0.79 | 1.53 |
| **hazelnuts + walnuts** | **6-11 months** | 0.24 | 0.53 | 1.10 | 0.48 | 1.05 | 2.20 | 0.72 | 1.58 | 3.30 | 0.96 | 2.11 | 4.41 | 1.19 | 2.63 | 5.51 | 1.43 | 3.16 | 6.61 | 1.67 | 3.69 | 7.71 | 1.91 | 4.22 | 8.81 | 2.15 | 4.74 | 9.91 | 2.39 | 5.27 | 11.01 |
|  | **1-<2 years** | 0.19 | 0.43 | 0.89 | 0.39 | 0.85 | 1.78 | 0.58 | 1.28 | 2.67 | 0.77 | 1.70 | 3.56 | 0.96 | 2.13 | 4.44 | 1.16 | 2.55 | 5.33 | 1.35 | 2.98 | 6.22 | 1.54 | 3.40 | 7.11 | 1.74 | 3.83 | 8.00 | 1.93 | 4.25 | 8.89 |
|  | **2-<3 years** | 0.16 | 0.35 | 0.73 | 0.32 | 0.70 | 1.47 | 0.48 | 1.05 | 2.20 | 0.64 | 1.41 | 2.94 | 0.80 | 1.76 | 3.67 | 0.96 | 2.11 | 4.41 | 1.11 | 2.46 | 5.14 | 1.27 | 2.81 | 5.87 | 1.43 | 3.16 | 6.61 | 1.59 | 3.51 | 7.34 |
|  | **3-<6 years** | 0.12 | 0.26 | 0.54 | 0.24 | 0.52 | 1.09 | 0.35 | 0.78 | 1.63 | 0.47 | 1.04 | 2.18 | 0.59 | 1.30 | 2.72 | 0.71 | 1.56 | 3.27 | 0.83 | 1.82 | 3.81 | 0.95 | 2.08 | 4.36 | 1.06 | 2.35 | 4.90 | 1.18 | 2.61 | 5.45 |
|  | **6-<11 years** | 0.07 | 0.15 | 0.32 | 0.14 | 0.30 | 0.64 | 0.21 | 0.46 | 0.96 | 0.28 | 0.61 | 1.27 | 0.35 | 0.76 | 1.59 | 0.41 | 0.91 | 1.91 | 0.48 | 1.07 | 2.23 | 0.55 | 1.22 | 2.55 | 0.62 | 1.37 | 2.87 | 0.69 | 1.52 | 3.19 |
|  | **11-<16 years** | 0.04 | 0.09 | 0.18 | 0.08 | 0.17 | 0.36 | 0.12 | 0.26 | 0.54 | 0.15 | 0.34 | 0.71 | 0.19 | 0.43 | 0.89 | 0.23 | 0.51 | 1.07 | 0.27 | 0.60 | 1.25 | 0.31 | 0.68 | 1.43 | 0.35 | 0.77 | 1.61 | 0.39 | 0.85 | 1.78 |
|  | **16-<21 years** | 0.03 | 0.07 | 0.14 | 0.06 | 0.14 | 0.28 | 0.09 | 0.20 | 0.42 | 0.12 | 0.27 | 0.57 | 0.15 | 0.34 | 0.71 | 0.18 | 0.41 | 0.85 | 0.21 | 0.47 | 0.99 | 0.25 | 0.54 | 1.13 | 0.28 | 0.61 | 1.27 | 0.31 | 0.68 | 1.42 |
|  | **adult** | 0.03 | 0.06 | 0.13 | 0.05 | 0.12 | 0.25 | 0.08 | 0.18 | 0.38 | 0.11 | 0.24 | 0.51 | 0.14 | 0.30 | 0.63 | 0.16 | 0.36 | 0.76 | 0.19 | 0.42 | 0.89 | 0.22 | 0.48 | 1.01 | 0.25 | 0.55 | 1.14 | 0.27 | 0.61 | 1.27 |

**Table D.2** Hazard Index (HI) calculated for Cd, Pb, Ni, and Cr taking into account exposure to all elements and the simultaneous consumption of three tested nut types

| **Nut type** |  | **Hazard Index scenario** | | | | | | | | | | | | | | | | | | | | | | | | | | | | | |
| --- | --- | --- | --- | --- | --- | --- | --- | --- | --- | --- | --- | --- | --- | --- | --- | --- | --- | --- | --- | --- | --- | --- | --- | --- | --- | --- | --- | --- | --- | --- | --- |
|  |  | **A** | | | **B** | | | **C** | | | **D** | | | **E** | | | **F** | | | **G** | | | **H** | | | **I** | | | **J** | | |
|  | **Age** | **I** | **II** | **III** | **I** | **II** | **III** | **I** | **II** | **III** | **I** | **II** | **III** | **I** | **II** | **III** | **I** | **II** | **III** | **I** | **II** | **III** | **I** | **II** | **III** | **I** | **II** | **III** | **I** | **II** | **III** |
| **peanuts + almonds + cashews** | **6-11 months** | 0.58 | 1.11 | 2.55 | 1.16 | 2.21 | 5.11 | 1.75 | 3.32 | 7.66 | 2.33 | 4.42 | 10.21 | 2.91 | 5.53 | 12.77 | 3.49 | 6.64 | 15.32 | 4.07 | 7.74 | 17.88 | 4.65 | 8.85 | 20.43 | 5.24 | 9.95 | 22.98 | 5.82 | 11.06 | 25.54 |
|  | **1-<2 years** | 0.47 | 0.89 | 2.06 | 0.94 | 1.78 | 4.12 | 1.41 | 2.68 | 6.18 | 1.88 | 3.57 | 8.24 | 2.35 | 4.46 | 10.30 | 2.82 | 5.35 | 12.37 | 3.29 | 6.25 | 14.43 | 3.76 | 7.14 | 16.49 | 4.23 | 8.03 | 18.55 | 4.69 | 8.92 | 20.61 |
|  | **2-<3 years** | 0.39 | 0.74 | 1.70 | 0.78 | 1.47 | 3.40 | 1.16 | 2.21 | 5.11 | 1.55 | 2.95 | 6.81 | 1.94 | 3.69 | 8.51 | 2.33 | 4.42 | 10.21 | 2.71 | 5.16 | 11.92 | 3.10 | 5.90 | 13.62 | 3.49 | 6.64 | 15.32 | 3.88 | 7.37 | 17.02 |
|  | **3-<6 years** | 0.29 | 0.55 | 1.26 | 0.58 | 1.09 | 2.53 | 0.86 | 1.64 | 3.79 | 1.15 | 2.19 | 5.05 | 1.44 | 2.73 | 6.32 | 1.73 | 3.28 | 7.58 | 2.01 | 3.83 | 8.84 | 2.30 | 4.38 | 10.10 | 2.59 | 4.92 | 11.37 | 2.88 | 5.47 | 12.63 |
|  | **6-<11 years** | 0.17 | 0.32 | 0.74 | 0.34 | 0.64 | 1.48 | 0.50 | 0.96 | 2.22 | 0.67 | 1.28 | 2.96 | 0.84 | 1.60 | 3.69 | 1.01 | 1.92 | 4.43 | 1.18 | 2.24 | 5.17 | 1.35 | 2.56 | 5.91 | 1.51 | 2.88 | 6.65 | 1.68 | 3.20 | 7.39 |
|  | **11-<16 years** | 0.09 | 0.18 | 0.41 | 0.19 | 0.36 | 0.83 | 0.28 | 0.54 | 1.24 | 0.38 | 0.72 | 1.65 | 0.47 | 0.90 | 2.07 | 0.57 | 1.07 | 2.48 | 0.66 | 1.25 | 2.90 | 0.75 | 1.43 | 3.31 | 0.85 | 1.61 | 3.72 | 0.94 | 1.79 | 4.14 |
|  | **16-<21 years** | 0.07 | 0.14 | 0.33 | 0.15 | 0.28 | 0.66 | 0.22 | 0.43 | 0.98 | 0.30 | 0.57 | 1.31 | 0.37 | 0.71 | 1.64 | 0.45 | 0.85 | 1.97 | 0.52 | 0.99 | 2.30 | 0.60 | 1.14 | 2.62 | 0.67 | 1.28 | 2.95 | 0.75 | 1.42 | 3.28 |
|  | **adult** | 0.07 | 0.13 | 0.29 | 0.13 | 0.25 | 0.59 | 0.20 | 0.38 | 0.88 | 0.27 | 0.51 | 1.17 | 0.33 | 0.64 | 1.47 | 0.40 | 0.76 | 1.76 | 0.47 | 0.89 | 2.06 | 0.54 | 1.02 | 2.35 | 0.60 | 1.14 | 2.64 | 0.67 | 1.27 | 2.94 |
| **peanuts + almonds + hazelnuts** | **6-11 months** | 0.42 | 0.95 | 2.32 | 0.85 | 1.90 | 4.65 | 1.27 | 2.85 | 6.97 | 1.70 | 3.79 | 9.30 | 2.12 | 4.74 | 11.62 | 2.55 | 5.69 | 13.94 | 2.97 | 6.64 | 16.27 | 3.40 | 7.59 | 18.59 | 3.82 | 8.54 | 20.92 | 4.25 | 9.48 | 23.24 |
|  | **1-<2 years** | 0.34 | 0.77 | 1.88 | 0.69 | 1.53 | 3.75 | 1.03 | 2.30 | 5.63 | 1.37 | 3.06 | 7.50 | 1.71 | 3.83 | 9.38 | 2.06 | 4.59 | 11.25 | 2.40 | 5.36 | 13.13 | 2.74 | 6.12 | 15.00 | 3.08 | 6.89 | 16.88 | 3.43 | 7.65 | 18.75 |
|  | **2-<3 years** | 0.28 | 0.63 | 1.55 | 0.57 | 1.26 | 3.10 | 0.85 | 1.90 | 4.65 | 1.13 | 2.53 | 6.20 | 1.42 | 3.16 | 7.75 | 1.70 | 3.79 | 9.30 | 1.98 | 4.43 | 10.84 | 2.26 | 5.06 | 12.39 | 2.55 | 5.69 | 13.94 | 2.83 | 6.32 | 15.49 |
|  | **3-<6 years** | 0.21 | 0.47 | 1.15 | 0.42 | 0.94 | 2.30 | 0.63 | 1.41 | 3.45 | 0.84 | 1.88 | 4.60 | 1.05 | 2.35 | 5.75 | 1.26 | 2.81 | 6.90 | 1.47 | 3.28 | 8.05 | 1.68 | 3.75 | 9.20 | 1.89 | 4.22 | 10.35 | 2.10 | 4.69 | 11.49 |
|  | **6-<11 years** | 0.12 | 0.27 | 0.67 | 0.25 | 0.55 | 1.34 | 0.37 | 0.82 | 2.02 | 0.49 | 1.10 | 2.69 | 0.61 | 1.37 | 3.36 | 0.74 | 1.65 | 4.03 | 0.86 | 1.92 | 4.71 | 0.98 | 2.20 | 5.38 | 1.11 | 2.47 | 6.05 | 1.23 | 2.74 | 6.72 |
|  | **11-<16 years** | 0.07 | 0.15 | 0.38 | 0.14 | 0.31 | 0.75 | 0.21 | 0.46 | 1.13 | 0.28 | 0.61 | 1.51 | 0.34 | 0.77 | 1.88 | 0.41 | 0.92 | 2.26 | 0.48 | 1.08 | 2.63 | 0.55 | 1.23 | 3.01 | 0.62 | 1.38 | 3.39 | 0.69 | 1.54 | 3.76 |
|  | **16-<21 years** | 0.05 | 0.12 | 0.30 | 0.11 | 0.24 | 0.60 | 0.16 | 0.37 | 0.90 | 0.22 | 0.49 | 1.19 | 0.27 | 0.61 | 1.49 | 0.33 | 0.73 | 1.79 | 0.38 | 0.85 | 2.09 | 0.44 | 0.97 | 2.39 | 0.49 | 1.10 | 2.69 | 0.55 | 1.22 | 2.99 |
|  | **adult** | 0.05 | 0.11 | 0.27 | 0.10 | 0.22 | 0.53 | 0.15 | 0.33 | 0.80 | 0.20 | 0.44 | 1.07 | 0.24 | 0.55 | 1.34 | 0.29 | 0.65 | 1.60 | 0.34 | 0.76 | 1.87 | 0.39 | 0.87 | 2.14 | 0.44 | 0.98 | 2.41 | 0.49 | 1.09 | 2.67 |
| **peanuts + almonds + walnuts** | **6-11 months** | 0.37 | 0.92 | 2.27 | 0.74 | 1.84 | 4.54 | 1.11 | 2.77 | 6.81 | 1.48 | 3.69 | 9.08 | 1.85 | 4.61 | 11.35 | 2.22 | 5.53 | 13.62 | 2.59 | 6.45 | 15.89 | 2.95 | 7.38 | 18.16 | 3.32 | 8.30 | 20.44 | 3.69 | 9.22 | 22.71 |
|  | **1-<2 years** | 0.30 | 0.74 | 1.83 | 0.60 | 1.49 | 3.66 | 0.89 | 2.23 | 5.50 | 1.19 | 2.98 | 7.33 | 1.49 | 3.72 | 9.16 | 1.79 | 4.46 | 10.99 | 2.09 | 5.21 | 12.83 | 2.38 | 5.95 | 14.66 | 2.68 | 6.70 | 16.49 | 2.98 | 7.44 | 18.32 |
|  | **2-<3 years** | 0.25 | 0.61 | 1.51 | 0.49 | 1.23 | 3.03 | 0.74 | 1.84 | 4.54 | 0.98 | 2.46 | 6.05 | 1.23 | 3.07 | 7.57 | 1.48 | 3.69 | 9.08 | 1.72 | 4.30 | 10.60 | 1.97 | 4.92 | 12.11 | 2.22 | 5.53 | 13.62 | 2.46 | 6.15 | 15.14 |
|  | **3-<6 years** | 0.18 | 0.46 | 1.12 | 0.37 | 0.91 | 2.25 | 0.55 | 1.37 | 3.37 | 0.73 | 1.82 | 4.49 | 0.91 | 2.28 | 5.62 | 1.10 | 2.74 | 6.74 | 1.28 | 3.19 | 7.86 | 1.46 | 3.65 | 8.98 | 1.64 | 4.10 | 10.11 | 1.83 | 4.56 | 11.23 |
|  | **6-<11 years** | 0.11 | 0.27 | 0.66 | 0.21 | 0.53 | 1.31 | 0.32 | 0.80 | 1.97 | 0.43 | 1.07 | 2.63 | 0.53 | 1.33 | 3.28 | 0.64 | 1.60 | 3.94 | 0.75 | 1.87 | 4.60 | 0.85 | 2.13 | 5.26 | 0.96 | 2.40 | 5.91 | 1.07 | 2.67 | 6.57 |
|  | **11-<16 years** | 0.06 | 0.15 | 0.37 | 0.12 | 0.30 | 0.74 | 0.18 | 0.45 | 1.10 | 0.24 | 0.60 | 1.47 | 0.30 | 0.75 | 1.84 | 0.36 | 0.90 | 2.21 | 0.42 | 1.05 | 2.57 | 0.48 | 1.19 | 2.94 | 0.54 | 1.34 | 3.31 | 0.60 | 1.49 | 3.68 |
|  | **16-<21 years** | 0.05 | 0.12 | 0.29 | 0.09 | 0.24 | 0.58 | 0.14 | 0.36 | 0.88 | 0.19 | 0.47 | 1.17 | 0.24 | 0.59 | 1.46 | 0.28 | 0.71 | 1.75 | 0.33 | 0.83 | 2.04 | 0.38 | 0.95 | 2.33 | 0.43 | 1.07 | 2.63 | 0.47 | 1.18 | 2.92 |
|  | **adult** | 0.04 | 0.11 | 0.26 | 0.08 | 0.21 | 0.52 | 0.13 | 0.32 | 0.78 | 0.17 | 0.42 | 1.04 | 0.21 | 0.53 | 1.31 | 0.25 | 0.64 | 1.57 | 0.30 | 0.74 | 1.83 | 0.34 | 0.85 | 2.09 | 0.38 | 0.95 | 2.35 | 0.42 | 1.06 | 2.61 |
| **peanuts + cashews + hazelnuts** | **6-11 months** | 0.60 | 1.13 | 2.54 | 1.20 | 2.25 | 5.08 | 1.79 | 3.38 | 7.63 | 2.39 | 4.51 | 10.17 | 2.99 | 5.63 | 12.71 | 3.59 | 6.76 | 15.25 | 4.18 | 7.88 | 17.80 | 4.78 | 9.01 | 20.34 | 5.38 | 10.14 | 22.88 | 5.98 | 11.26 | 25.42 |
|  | **1-<2 years** | 0.48 | 0.91 | 2.05 | 0.96 | 1.82 | 4.10 | 1.45 | 2.73 | 6.16 | 1.93 | 3.64 | 8.21 | 2.41 | 4.54 | 10.26 | 2.89 | 5.45 | 12.31 | 3.38 | 6.36 | 14.36 | 3.86 | 7.27 | 16.41 | 4.34 | 8.18 | 18.47 | 4.82 | 9.09 | 20.52 |
|  | **2-<3 years** | 0.40 | 0.75 | 1.69 | 0.80 | 1.50 | 3.39 | 1.20 | 2.25 | 5.08 | 1.59 | 3.00 | 6.78 | 1.99 | 3.75 | 8.47 | 2.39 | 4.51 | 10.17 | 2.79 | 5.26 | 11.86 | 3.19 | 6.01 | 13.56 | 3.59 | 6.76 | 15.25 | 3.98 | 7.51 | 16.95 |
|  | **3-<6 years** | 0.30 | 0.56 | 1.26 | 0.59 | 1.11 | 2.52 | 0.89 | 1.67 | 3.77 | 1.18 | 2.23 | 5.03 | 1.48 | 2.79 | 6.29 | 1.77 | 3.34 | 7.55 | 2.07 | 3.90 | 8.80 | 2.36 | 4.46 | 10.06 | 2.66 | 5.01 | 11.32 | 2.96 | 5.57 | 12.58 |
|  | **6-<11 years** | 0.17 | 0.33 | 0.74 | 0.35 | 0.65 | 1.47 | 0.52 | 0.98 | 2.21 | 0.69 | 1.30 | 2.94 | 0.86 | 1.63 | 3.68 | 1.04 | 1.96 | 4.41 | 1.21 | 2.28 | 5.15 | 1.38 | 2.61 | 5.88 | 1.56 | 2.93 | 6.62 | 1.73 | 3.26 | 7.36 |
|  | **11-<16 years** | 0.10 | 0.18 | 0.41 | 0.19 | 0.36 | 0.82 | 0.29 | 0.55 | 1.24 | 0.39 | 0.73 | 1.65 | 0.48 | 0.91 | 2.06 | 0.58 | 1.09 | 2.47 | 0.68 | 1.28 | 2.88 | 0.77 | 1.46 | 3.29 | 0.87 | 1.64 | 3.71 | 0.97 | 1.82 | 4.12 |
|  | **16-<21 years** | 0.08 | 0.14 | 0.33 | 0.15 | 0.29 | 0.65 | 0.23 | 0.43 | 0.98 | 0.31 | 0.58 | 1.31 | 0.38 | 0.72 | 1.63 | 0.46 | 0.87 | 1.96 | 0.54 | 1.01 | 2.29 | 0.61 | 1.16 | 2.61 | 0.69 | 1.30 | 2.94 | 0.77 | 1.45 | 3.27 |
|  | **adult** | 0.07 | 0.13 | 0.29 | 0.14 | 0.26 | 0.58 | 0.21 | 0.39 | 0.88 | 0.27 | 0.52 | 1.17 | 0.34 | 0.65 | 1.46 | 0.41 | 0.78 | 1.75 | 0.48 | 0.91 | 2.05 | 0.55 | 1.04 | 2.34 | 0.62 | 1.17 | 2.63 | 0.69 | 1.30 | 2.92 |
| **peanuts + cashews + walnuts** | **6-11 months** | 0.54 | 1.10 | 2.49 | 1.08 | 2.20 | 4.98 | 1.63 | 3.30 | 7.47 | 2.17 | 4.40 | 9.96 | 2.71 | 5.50 | 12.45 | 3.25 | 6.60 | 14.93 | 3.80 | 7.70 | 17.42 | 4.34 | 8.80 | 19.91 | 4.88 | 9.90 | 22.40 | 5.42 | 11.00 | 24.89 |
|  | **1-<2 years** | 0.44 | 0.89 | 2.01 | 0.88 | 1.78 | 4.02 | 1.31 | 2.66 | 6.03 | 1.75 | 3.55 | 8.04 | 2.19 | 4.44 | 10.04 | 2.63 | 5.33 | 12.05 | 3.06 | 6.21 | 14.06 | 3.50 | 7.10 | 16.07 | 3.94 | 7.99 | 18.08 | 4.38 | 8.88 | 20.09 |
|  | **2-<3 years** | 0.36 | 0.73 | 1.66 | 0.72 | 1.47 | 3.32 | 1.08 | 2.20 | 4.98 | 1.45 | 2.93 | 6.64 | 1.81 | 3.67 | 8.30 | 2.17 | 4.40 | 9.96 | 2.53 | 5.13 | 11.62 | 2.89 | 5.87 | 13.28 | 3.25 | 6.60 | 14.93 | 3.62 | 7.33 | 16.59 |
|  | **3-<6 years** | 0.27 | 0.54 | 1.23 | 0.54 | 1.09 | 2.46 | 0.80 | 1.63 | 3.69 | 1.07 | 2.18 | 4.92 | 1.34 | 2.72 | 6.16 | 1.61 | 3.26 | 7.39 | 1.88 | 3.81 | 8.62 | 2.15 | 4.35 | 9.85 | 2.41 | 4.90 | 11.08 | 2.68 | 5.44 | 12.31 |
|  | **6-<11 years** | 0.16 | 0.32 | 0.72 | 0.31 | 0.64 | 1.44 | 0.47 | 0.95 | 2.16 | 0.63 | 1.27 | 2.88 | 0.78 | 1.59 | 3.60 | 0.94 | 1.91 | 4.32 | 1.10 | 2.23 | 5.04 | 1.26 | 2.55 | 5.76 | 1.41 | 2.86 | 6.48 | 1.57 | 3.18 | 7.20 |
|  | **11-<16 years** | 0.09 | 0.18 | 0.40 | 0.18 | 0.36 | 0.81 | 0.26 | 0.53 | 1.21 | 0.35 | 0.71 | 1.61 | 0.44 | 0.89 | 2.02 | 0.53 | 1.07 | 2.42 | 0.61 | 1.25 | 2.82 | 0.70 | 1.43 | 3.23 | 0.79 | 1.60 | 3.63 | 0.88 | 1.78 | 4.03 |
|  | **16-<21 years** | 0.07 | 0.14 | 0.32 | 0.14 | 0.28 | 0.64 | 0.21 | 0.42 | 0.96 | 0.28 | 0.57 | 1.28 | 0.35 | 0.71 | 1.60 | 0.42 | 0.85 | 1.92 | 0.49 | 0.99 | 2.24 | 0.56 | 1.13 | 2.56 | 0.63 | 1.27 | 2.88 | 0.70 | 1.41 | 3.20 |
|  | **adult** | 0.06 | 0.13 | 0.29 | 0.12 | 0.25 | 0.57 | 0.19 | 0.38 | 0.86 | 0.25 | 0.51 | 1.15 | 0.31 | 0.63 | 1.43 | 0.37 | 0.76 | 1.72 | 0.44 | 0.89 | 2.00 | 0.50 | 1.01 | 2.29 | 0.56 | 1.14 | 2.58 | 0.62 | 1.26 | 2.86 |
| **peanuts + hazelnuts + walnuts** | **6-11 months** | 0.39 | 0.94 | 2.26 | 0.77 | 1.89 | 4.52 | 1.16 | 2.83 | 6.78 | 1.54 | 3.77 | 9.04 | 1.93 | 4.71 | 11.30 | 2.31 | 5.66 | 13.56 | 2.70 | 6.60 | 15.82 | 3.08 | 7.54 | 18.08 | 3.47 | 8.48 | 20.33 | 3.85 | 9.43 | 22.59 |
|  | **1-<2 years** | 0.31 | 0.76 | 1.82 | 0.62 | 1.52 | 3.65 | 0.93 | 2.28 | 5.47 | 1.24 | 3.04 | 7.29 | 1.55 | 3.80 | 9.12 | 1.86 | 4.56 | 10.94 | 2.18 | 5.32 | 12.76 | 2.49 | 6.09 | 14.59 | 2.80 | 6.85 | 16.41 | 3.11 | 7.61 | 18.23 |
|  | **2-<3 years** | 0.26 | 0.63 | 1.51 | 0.51 | 1.26 | 3.01 | 0.77 | 1.89 | 4.52 | 1.03 | 2.51 | 6.03 | 1.28 | 3.14 | 7.53 | 1.54 | 3.77 | 9.04 | 1.80 | 4.40 | 10.54 | 2.05 | 5.03 | 12.05 | 2.31 | 5.66 | 13.56 | 2.57 | 6.28 | 15.06 |
|  | **3-<6 years** | 0.19 | 0.47 | 1.12 | 0.38 | 0.93 | 2.24 | 0.57 | 1.40 | 3.35 | 0.76 | 1.86 | 4.47 | 0.95 | 2.33 | 5.59 | 1.14 | 2.80 | 6.71 | 1.33 | 3.26 | 7.82 | 1.52 | 3.73 | 8.94 | 1.71 | 4.20 | 10.06 | 1.91 | 4.66 | 11.18 |
|  | **6-<11 years** | 0.11 | 0.27 | 0.65 | 0.22 | 0.55 | 1.31 | 0.33 | 0.82 | 1.96 | 0.45 | 1.09 | 2.61 | 0.56 | 1.36 | 3.27 | 0.67 | 1.64 | 3.92 | 0.78 | 1.91 | 4.58 | 0.89 | 2.18 | 5.23 | 1.00 | 2.45 | 5.88 | 1.11 | 2.73 | 6.54 |
|  | **11-<16 years** | 0.06 | 0.15 | 0.37 | 0.12 | 0.31 | 0.73 | 0.19 | 0.46 | 1.10 | 0.25 | 0.61 | 1.46 | 0.31 | 0.76 | 1.83 | 0.37 | 0.92 | 2.20 | 0.44 | 1.07 | 2.56 | 0.50 | 1.22 | 2.93 | 0.56 | 1.37 | 3.29 | 0.62 | 1.53 | 3.66 |
|  | **16-<21 years** | 0.05 | 0.12 | 0.29 | 0.10 | 0.24 | 0.58 | 0.15 | 0.36 | 0.87 | 0.20 | 0.48 | 1.16 | 0.25 | 0.61 | 1.45 | 0.30 | 0.73 | 1.74 | 0.35 | 0.85 | 2.03 | 0.40 | 0.97 | 2.32 | 0.45 | 1.09 | 2.61 | 0.49 | 1.21 | 2.90 |
|  | **adult** | 0.04 | 0.11 | 0.26 | 0.09 | 0.22 | 0.52 | 0.13 | 0.33 | 0.78 | 0.18 | 0.43 | 1.04 | 0.22 | 0.54 | 1.30 | 0.27 | 0.65 | 1.56 | 0.31 | 0.76 | 1.82 | 0.35 | 0.87 | 2.08 | 0.40 | 0.98 | 2.34 | 0.44 | 1.08 | 2.60 |
| **almonds + cashews + hazelnuts** | **6-11 months** | 0.58 | 0.97 | 1.97 | 1.17 | 1.93 | 3.95 | 1.75 | 2.90 | 5.92 | 2.33 | 3.87 | 7.89 | 2.91 | 4.83 | 9.87 | 3.50 | 5.80 | 11.84 | 4.08 | 6.77 | 13.81 | 4.66 | 7.73 | 15.78 | 5.24 | 8.70 | 17.76 | 5.83 | 9.67 | 19.73 |
|  | **1-<2 years** | 0.47 | 0.78 | 1.59 | 0.94 | 1.56 | 3.18 | 1.41 | 2.34 | 4.78 | 1.88 | 3.12 | 6.37 | 2.35 | 3.90 | 7.96 | 2.82 | 4.68 | 9.55 | 3.29 | 5.46 | 11.15 | 3.76 | 6.24 | 12.74 | 4.23 | 7.02 | 14.33 | 4.70 | 7.80 | 15.92 |
|  | **2-<3 years** | 0.39 | 0.64 | 1.32 | 0.78 | 1.29 | 2.63 | 1.17 | 1.93 | 3.95 | 1.55 | 2.58 | 5.26 | 1.94 | 3.22 | 6.58 | 2.33 | 3.87 | 7.89 | 2.72 | 4.51 | 9.21 | 3.11 | 5.16 | 10.52 | 3.50 | 5.80 | 11.84 | 3.88 | 6.45 | 13.15 |
|  | **3-<6 years** | 0.29 | 0.48 | 0.98 | 0.58 | 0.96 | 1.95 | 0.86 | 1.43 | 2.93 | 1.15 | 1.91 | 3.90 | 1.44 | 2.39 | 4.88 | 1.73 | 2.87 | 5.86 | 2.02 | 3.35 | 6.83 | 2.31 | 3.83 | 7.81 | 2.59 | 4.30 | 8.78 | 2.88 | 4.78 | 9.76 |
|  | **6-<11 years** | 0.17 | 0.28 | 0.57 | 0.34 | 0.56 | 1.14 | 0.51 | 0.84 | 1.71 | 0.67 | 1.12 | 2.28 | 0.84 | 1.40 | 2.85 | 1.01 | 1.68 | 3.42 | 1.18 | 1.96 | 4.00 | 1.35 | 2.24 | 4.57 | 1.52 | 2.52 | 5.14 | 1.69 | 2.80 | 5.71 |
|  | **11-<16 years** | 0.09 | 0.16 | 0.32 | 0.19 | 0.31 | 0.64 | 0.28 | 0.47 | 0.96 | 0.38 | 0.63 | 1.28 | 0.47 | 0.78 | 1.60 | 0.57 | 0.94 | 1.92 | 0.66 | 1.10 | 2.24 | 0.75 | 1.25 | 2.56 | 0.85 | 1.41 | 2.88 | 0.94 | 1.57 | 3.20 |
|  | **16-<21 years** | 0.07 | 0.12 | 0.25 | 0.15 | 0.25 | 0.51 | 0.22 | 0.37 | 0.76 | 0.30 | 0.50 | 1.01 | 0.37 | 0.62 | 1.27 | 0.45 | 0.75 | 1.52 | 0.52 | 0.87 | 1.77 | 0.60 | 0.99 | 2.03 | 0.67 | 1.12 | 2.28 | 0.75 | 1.24 | 2.54 |
|  | **adult** | 0.07 | 0.11 | 0.23 | 0.13 | 0.22 | 0.45 | 0.20 | 0.33 | 0.68 | 0.27 | 0.44 | 0.91 | 0.33 | 0.56 | 1.13 | 0.40 | 0.67 | 1.36 | 0.47 | 0.78 | 1.59 | 0.54 | 0.89 | 1.82 | 0.60 | 1.00 | 2.04 | 0.67 | 1.11 | 2.27 |
| **almonds + cashews + walnuts** | **6-11 months** | 0.53 | 0.94 | 1.92 | 1.05 | 1.88 | 3.84 | 1.58 | 2.82 | 5.76 | 2.11 | 3.76 | 7.68 | 2.64 | 4.70 | 9.60 | 3.16 | 5.64 | 11.52 | 3.69 | 6.58 | 13.44 | 4.22 | 7.52 | 15.36 | 4.75 | 8.46 | 17.28 | 5.27 | 9.40 | 19.20 |
|  | **1-<2 years** | 0.43 | 0.76 | 1.55 | 0.85 | 1.52 | 3.10 | 1.28 | 2.28 | 4.65 | 1.70 | 3.04 | 6.20 | 2.13 | 3.79 | 7.75 | 2.55 | 4.55 | 9.30 | 2.98 | 5.31 | 10.84 | 3.40 | 6.07 | 12.39 | 3.83 | 6.83 | 13.94 | 4.26 | 7.59 | 15.49 |
|  | **2-<3 years** | 0.35 | 0.63 | 1.28 | 0.70 | 1.25 | 2.56 | 1.05 | 1.88 | 3.84 | 1.41 | 2.51 | 5.12 | 1.76 | 3.13 | 6.40 | 2.11 | 3.76 | 7.68 | 2.46 | 4.39 | 8.96 | 2.81 | 5.02 | 10.24 | 3.16 | 5.64 | 11.52 | 3.52 | 6.27 | 12.80 |
|  | **3-<6 years** | 0.26 | 0.47 | 0.95 | 0.52 | 0.93 | 1.90 | 0.78 | 1.40 | 2.85 | 1.04 | 1.86 | 3.80 | 1.30 | 2.33 | 4.75 | 1.56 | 2.79 | 5.70 | 1.83 | 3.26 | 6.65 | 2.09 | 3.72 | 7.60 | 2.35 | 4.19 | 8.55 | 2.61 | 4.65 | 9.50 |
|  | **6-<11 years** | 0.15 | 0.27 | 0.56 | 0.31 | 0.54 | 1.11 | 0.46 | 0.82 | 1.67 | 0.61 | 1.09 | 2.22 | 0.76 | 1.36 | 2.78 | 0.92 | 1.63 | 3.33 | 1.07 | 1.90 | 3.89 | 1.22 | 2.18 | 4.44 | 1.37 | 2.45 | 5.00 | 1.53 | 2.72 | 5.55 |
|  | **11-<16 years** | 0.09 | 0.15 | 0.31 | 0.17 | 0.30 | 0.62 | 0.26 | 0.46 | 0.93 | 0.34 | 0.61 | 1.24 | 0.43 | 0.76 | 1.55 | 0.51 | 0.91 | 1.87 | 0.60 | 1.07 | 2.18 | 0.68 | 1.22 | 2.49 | 0.77 | 1.37 | 2.80 | 0.85 | 1.52 | 3.11 |
|  | **16-<21 years** | 0.07 | 0.12 | 0.25 | 0.14 | 0.24 | 0.49 | 0.20 | 0.36 | 0.74 | 0.27 | 0.48 | 0.99 | 0.34 | 0.60 | 1.23 | 0.41 | 0.73 | 1.48 | 0.47 | 0.85 | 1.73 | 0.54 | 0.97 | 1.97 | 0.61 | 1.09 | 2.22 | 0.68 | 1.21 | 2.47 |
|  | **adult** | 0.06 | 0.11 | 0.22 | 0.12 | 0.22 | 0.44 | 0.18 | 0.32 | 0.66 | 0.24 | 0.43 | 0.88 | 0.30 | 0.54 | 1.10 | 0.36 | 0.65 | 1.32 | 0.42 | 0.76 | 1.55 | 0.49 | 0.87 | 1.77 | 0.55 | 0.97 | 1.99 | 0.61 | 1.08 | 2.21 |
| **almonds + walnuts + hazelnuts** | **6-11 months** | 0.37 | 0.78 | 1.69 | 0.74 | 1.57 | 3.38 | 1.11 | 2.35 | 5.07 | 1.48 | 3.13 | 6.76 | 1.85 | 3.92 | 8.45 | 2.22 | 4.70 | 10.14 | 2.59 | 5.48 | 11.83 | 2.96 | 6.26 | 13.52 | 3.33 | 7.05 | 15.21 | 3.70 | 7.83 | 16.90 |
|  | **1-<2 years** | 0.30 | 0.63 | 1.36 | 0.60 | 1.26 | 2.73 | 0.90 | 1.90 | 4.09 | 1.19 | 2.53 | 5.46 | 1.49 | 3.16 | 6.82 | 1.79 | 3.79 | 8.18 | 2.09 | 4.42 | 9.55 | 2.39 | 5.06 | 10.91 | 2.69 | 5.69 | 12.27 | 2.99 | 6.32 | 13.64 |
|  | **2-<3 years** | 0.25 | 0.52 | 1.13 | 0.49 | 1.04 | 2.25 | 0.74 | 1.57 | 3.38 | 0.99 | 2.09 | 4.51 | 1.23 | 2.61 | 5.63 | 1.48 | 3.13 | 6.76 | 1.73 | 3.65 | 7.89 | 1.97 | 4.18 | 9.01 | 2.22 | 4.70 | 10.14 | 2.47 | 5.22 | 11.27 |
|  | **3-<6 years** | 0.18 | 0.39 | 0.84 | 0.37 | 0.77 | 1.67 | 0.55 | 1.16 | 2.51 | 0.73 | 1.55 | 3.34 | 0.92 | 1.94 | 4.18 | 1.10 | 2.32 | 5.02 | 1.28 | 2.71 | 5.85 | 1.46 | 3.10 | 6.69 | 1.65 | 3.49 | 7.52 | 1.83 | 3.87 | 8.36 |
|  | **6-<11 years** | 0.11 | 0.23 | 0.49 | 0.21 | 0.45 | 0.98 | 0.32 | 0.68 | 1.47 | 0.43 | 0.91 | 1.96 | 0.54 | 1.13 | 2.44 | 0.64 | 1.36 | 2.93 | 0.75 | 1.59 | 3.42 | 0.86 | 1.81 | 3.91 | 0.96 | 2.04 | 4.40 | 1.07 | 2.27 | 4.89 |
|  | **11-<16 years** | 0.06 | 0.13 | 0.27 | 0.12 | 0.25 | 0.55 | 0.18 | 0.38 | 0.82 | 0.24 | 0.51 | 1.09 | 0.30 | 0.63 | 1.37 | 0.36 | 0.76 | 1.64 | 0.42 | 0.89 | 1.92 | 0.48 | 1.01 | 2.19 | 0.54 | 1.14 | 2.46 | 0.60 | 1.27 | 2.74 |
|  | **16-<21 years** | 0.05 | 0.10 | 0.22 | 0.10 | 0.20 | 0.43 | 0.14 | 0.30 | 0.65 | 0.19 | 0.40 | 0.87 | 0.24 | 0.50 | 1.09 | 0.29 | 0.60 | 1.30 | 0.33 | 0.70 | 1.52 | 0.38 | 0.80 | 1.74 | 0.43 | 0.91 | 1.95 | 0.48 | 1.01 | 2.17 |
|  | **adult** | 0.04 | 0.09 | 0.19 | 0.09 | 0.18 | 0.39 | 0.13 | 0.27 | 0.58 | 0.17 | 0.36 | 0.78 | 0.21 | 0.45 | 0.97 | 0.26 | 0.54 | 1.17 | 0.30 | 0.63 | 1.36 | 0.34 | 0.72 | 1.55 | 0.38 | 0.81 | 1.75 | 0.43 | 0.90 | 1.94 |
| **cashews + hazelnuts + walnuts** | **6-11 months** | 0.54 | 0.96 | 1.91 | 1.09 | 1.92 | 3.82 | 1.63 | 2.88 | 5.73 | 2.17 | 3.84 | 7.63 | 2.72 | 4.80 | 9.54 | 3.26 | 5.77 | 11.45 | 3.80 | 6.73 | 13.36 | 4.35 | 7.69 | 15.27 | 4.89 | 8.65 | 17.18 | 5.43 | 9.61 | 19.09 |
|  | **1-<2 years** | 0.44 | 0.78 | 1.54 | 0.88 | 1.55 | 3.08 | 1.32 | 2.33 | 4.62 | 1.75 | 3.10 | 6.16 | 2.19 | 3.88 | 7.70 | 2.63 | 4.65 | 9.24 | 3.07 | 5.43 | 10.78 | 3.51 | 6.20 | 12.32 | 3.95 | 6.98 | 13.86 | 4.38 | 7.75 | 15.40 |
|  | **2-<3 years** | 0.36 | 0.64 | 1.27 | 0.72 | 1.28 | 2.54 | 1.09 | 1.92 | 3.82 | 1.45 | 2.56 | 5.09 | 1.81 | 3.20 | 6.36 | 2.17 | 3.84 | 7.63 | 2.53 | 4.48 | 8.91 | 2.90 | 5.12 | 10.18 | 3.26 | 5.77 | 11.45 | 3.62 | 6.41 | 12.72 |
|  | **3-<6 years** | 0.27 | 0.48 | 0.94 | 0.54 | 0.95 | 1.89 | 0.81 | 1.43 | 2.83 | 1.07 | 1.90 | 3.78 | 1.34 | 2.38 | 4.72 | 1.61 | 2.85 | 5.66 | 1.88 | 3.33 | 6.61 | 2.15 | 3.80 | 7.55 | 2.42 | 4.28 | 8.50 | 2.69 | 4.75 | 9.44 |
|  | **6-<11 years** | 0.16 | 0.28 | 0.55 | 0.31 | 0.56 | 1.10 | 0.47 | 0.83 | 1.66 | 0.63 | 1.11 | 2.21 | 0.79 | 1.39 | 2.76 | 0.94 | 1.67 | 3.31 | 1.10 | 1.95 | 3.87 | 1.26 | 2.22 | 4.42 | 1.41 | 2.50 | 4.97 | 1.57 | 2.78 | 5.52 |
|  | **11-<16 years** | 0.09 | 0.16 | 0.31 | 0.18 | 0.31 | 0.62 | 0.26 | 0.47 | 0.93 | 0.35 | 0.62 | 1.24 | 0.44 | 0.78 | 1.55 | 0.53 | 0.93 | 1.85 | 0.62 | 1.09 | 2.16 | 0.70 | 1.25 | 2.47 | 0.79 | 1.40 | 2.78 | 0.88 | 1.56 | 3.09 |
|  | **16-<21 years** | 0.07 | 0.12 | 0.25 | 0.14 | 0.25 | 0.49 | 0.21 | 0.37 | 0.74 | 0.28 | 0.49 | 0.98 | 0.35 | 0.62 | 1.23 | 0.42 | 0.74 | 1.47 | 0.49 | 0.86 | 1.72 | 0.56 | 0.99 | 1.96 | 0.63 | 1.11 | 2.21 | 0.70 | 1.23 | 2.45 |
|  | **adult** | 0.06 | 0.11 | 0.22 | 0.12 | 0.22 | 0.44 | 0.19 | 0.33 | 0.66 | 0.25 | 0.44 | 0.88 | 0.31 | 0.55 | 1.10 | 0.37 | 0.66 | 1.32 | 0.44 | 0.77 | 1.54 | 0.50 | 0.88 | 1.76 | 0.56 | 0.99 | 1.98 | 0.62 | 1.11 | 2.19 |

**Table D.3** Hazard Index (HI) calculated for Cd, Pb, Ni, and Cr taking into account exposure to all elements and the simultaneous consumption of four tested nut types

| **Nut type** |  | **Hazard Index scenario** | | | | | | | | | | | | | | | | | | | | | | | | | | | | | |
| --- | --- | --- | --- | --- | --- | --- | --- | --- | --- | --- | --- | --- | --- | --- | --- | --- | --- | --- | --- | --- | --- | --- | --- | --- | --- | --- | --- | --- | --- | --- | --- |
|  |  | **A** | | | **B** | | | **C** | | | **D** | | | **E** | | | **F** | | | **G** | | | **H** | | | **I** | | | **J** | | |
|  | **Age** | **I** | **II** | **III** | **I** | **II** | **III** | **I** | **II** | **III** | **I** | **II** | **III** | **I** | **II** | **III** | **I** | **II** | **III** | **I** | **II** | **III** | **I** | **II** | **III** | **I** | **II** | **III** | **I** | **II** | **III** |
| **peanuts + almonds + cashews +hazelnuts** | **6-11 months** | 0.73 | 1.38 | 3.13 | 1.46 | 2.76 | 6.26 | 2.19 | 4.15 | 9.39 | 2.92 | 5.53 | 12.52 | 3.64 | 6.91 | 15.66 | 4.37 | 8.29 | 18.79 | 5.10 | 9.68 | 21.92 | 5.83 | 11.06 | 25.05 | 6.56 | 12.44 | 28.18 | 7.29 | 13.82 | 31.31 |
|  | **1-<2 years** | 0.59 | 1.12 | 2.53 | 1.18 | 2.23 | 5.05 | 1.76 | 3.35 | 7.58 | 2.35 | 4.46 | 10.11 | 2.94 | 5.58 | 12.63 | 3.53 | 6.69 | 15.16 | 4.12 | 7.81 | 17.69 | 4.71 | 8.93 | 20.21 | 5.29 | 10.04 | 22.74 | 5.88 | 11.16 | 25.27 |
|  | **2-<3 years** | 0.49 | 0.92 | 2.09 | 0.97 | 1.84 | 4.17 | 1.46 | 2.76 | 6.26 | 1.94 | 3.69 | 8.35 | 2.43 | 4.61 | 10.44 | 2.92 | 5.53 | 12.52 | 3.40 | 6.45 | 14.61 | 3.89 | 7.37 | 16.70 | 4.37 | 8.29 | 18.79 | 4.86 | 9.22 | 20.87 |
|  | **3-<6 years** | 0.36 | 0.68 | 1.55 | 0.72 | 1.37 | 3.10 | 1.08 | 2.05 | 4.65 | 1.44 | 2.74 | 6.19 | 1.80 | 3.42 | 7.74 | 2.16 | 4.10 | 9.29 | 2.52 | 4.79 | 10.84 | 2.88 | 5.47 | 12.39 | 3.24 | 6.15 | 13.94 | 3.60 | 6.84 | 15.49 |
|  | **6-<11 years** | 0.21 | 0.40 | 0.91 | 0.42 | 0.80 | 1.81 | 0.63 | 1.20 | 2.72 | 0.84 | 1.60 | 3.62 | 1.05 | 2.00 | 4.53 | 1.27 | 2.40 | 5.43 | 1.48 | 2.80 | 6.34 | 1.69 | 3.20 | 7.25 | 1.90 | 3.60 | 8.15 | 2.11 | 4.00 | 9.06 |
|  | **11-<16 years** | 0.12 | 0.22 | 0.51 | 0.24 | 0.45 | 1.01 | 0.35 | 0.67 | 1.52 | 0.47 | 0.90 | 2.03 | 0.59 | 1.12 | 2.54 | 0.71 | 1.34 | 3.04 | 0.83 | 1.57 | 3.55 | 0.94 | 1.79 | 4.06 | 1.06 | 2.02 | 4.56 | 1.18 | 2.24 | 5.07 |
|  | **16-<21 years** | 0.09 | 0.18 | 0.40 | 0.19 | 0.36 | 0.80 | 0.28 | 0.53 | 1.21 | 0.37 | 0.71 | 1.61 | 0.47 | 0.89 | 2.01 | 0.56 | 1.07 | 2.41 | 0.66 | 1.24 | 2.82 | 0.75 | 1.42 | 3.22 | 0.84 | 1.60 | 3.62 | 0.94 | 1.78 | 4.02 |
|  | **adult** | 0.08 | 0.16 | 0.36 | 0.17 | 0.32 | 0.72 | 0.25 | 0.48 | 1.08 | 0.34 | 0.64 | 1.44 | 0.42 | 0.79 | 1.80 | 0.50 | 0.95 | 2.16 | 0.59 | 1.11 | 2.52 | 0.67 | 1.27 | 2.88 | 0.75 | 1.43 | 3.24 | 0.84 | 1.59 | 3.60 |
| **peanuts + almonds + cashews +walnuts** | **6-11 months** | 0.67 | 1.36 | 3.08 | 1.35 | 2.71 | 6.16 | 2.02 | 4.07 | 9.23 | 2.69 | 5.42 | 12.31 | 3.37 | 6.78 | 15.39 | 4.04 | 8.14 | 18.47 | 4.72 | 9.49 | 21.54 | 5.39 | 10.85 | 24.62 | 6.06 | 12.21 | 27.70 | 6.74 | 13.56 | 30.78 |
|  | **1-<2 years** | 0.54 | 1.09 | 2.48 | 1.09 | 2.19 | 4.97 | 1.63 | 3.28 | 7.45 | 2.17 | 4.38 | 9.94 | 2.72 | 5.47 | 12.42 | 3.26 | 6.57 | 14.90 | 3.81 | 7.66 | 17.39 | 4.35 | 8.76 | 19.87 | 4.89 | 9.85 | 22.35 | 5.44 | 10.94 | 24.84 |
|  | **2-<3 years** | 0.45 | 0.90 | 2.05 | 0.90 | 1.81 | 4.10 | 1.35 | 2.71 | 6.16 | 1.80 | 3.62 | 8.21 | 2.25 | 4.52 | 10.26 | 2.69 | 5.42 | 12.31 | 3.14 | 6.33 | 14.36 | 3.59 | 7.23 | 16.41 | 4.04 | 8.14 | 18.47 | 4.49 | 9.04 | 20.52 |
|  | **3-<6 years** | 0.33 | 0.67 | 1.52 | 0.67 | 1.34 | 3.04 | 1.00 | 2.01 | 4.57 | 1.33 | 2.68 | 6.09 | 1.67 | 3.35 | 7.61 | 2.00 | 4.02 | 9.13 | 2.33 | 4.70 | 10.66 | 2.67 | 5.37 | 12.18 | 3.00 | 6.04 | 13.70 | 3.33 | 6.71 | 15.22 |
|  | **6-<11 years** | 0.19 | 0.39 | 0.89 | 0.39 | 0.78 | 1.78 | 0.58 | 1.18 | 2.67 | 0.78 | 1.57 | 3.56 | 0.97 | 1.96 | 4.45 | 1.17 | 2.35 | 5.34 | 1.36 | 2.75 | 6.23 | 1.56 | 3.14 | 7.12 | 1.75 | 3.53 | 8.01 | 1.95 | 3.92 | 8.90 |
|  | **11-<16 years** | 0.11 | 0.22 | 0.50 | 0.22 | 0.44 | 1.00 | 0.33 | 0.66 | 1.50 | 0.44 | 0.88 | 1.99 | 0.55 | 1.10 | 2.49 | 0.65 | 1.32 | 2.99 | 0.76 | 1.54 | 3.49 | 0.87 | 1.76 | 3.99 | 0.98 | 1.98 | 4.49 | 1.09 | 2.20 | 4.99 |
|  | **16-<21 years** | 0.09 | 0.17 | 0.40 | 0.17 | 0.35 | 0.79 | 0.26 | 0.52 | 1.19 | 0.35 | 0.70 | 1.58 | 0.43 | 0.87 | 1.98 | 0.52 | 1.05 | 2.37 | 0.61 | 1.22 | 2.77 | 0.69 | 1.39 | 3.16 | 0.78 | 1.57 | 3.56 | 0.87 | 1.74 | 3.95 |
|  | **adult** | 0.08 | 0.16 | 0.35 | 0.15 | 0.31 | 0.71 | 0.23 | 0.47 | 1.06 | 0.31 | 0.62 | 1.42 | 0.39 | 0.78 | 1.77 | 0.46 | 0.94 | 2.12 | 0.54 | 1.09 | 2.48 | 0.62 | 1.25 | 2.83 | 0.70 | 1.40 | 3.19 | 0.77 | 1.56 | 3.54 |
| **peanuts + almonds + hazelnuts + walnuts** | **6-11 months** | 0.52 | 1.20 | 2.85 | 1.03 | 2.40 | 5.70 | 1.55 | 3.60 | 8.54 | 2.07 | 4.79 | 11.39 | 2.58 | 5.99 | 14.24 | 3.10 | 7.19 | 17.09 | 3.61 | 8.39 | 19.94 | 4.13 | 9.59 | 22.78 | 4.65 | 10.79 | 25.63 | 5.16 | 11.99 | 28.48 |
|  | **1-<2 years** | 0.42 | 0.97 | 2.30 | 0.83 | 1.93 | 4.60 | 1.25 | 2.90 | 6.90 | 1.67 | 3.87 | 9.19 | 2.08 | 4.84 | 11.49 | 2.50 | 5.80 | 13.79 | 2.92 | 6.77 | 16.09 | 3.33 | 7.74 | 18.39 | 3.75 | 8.71 | 20.69 | 4.17 | 9.67 | 22.98 |
|  | **2-<3 years** | 0.34 | 0.80 | 1.90 | 0.69 | 1.60 | 3.80 | 1.03 | 2.40 | 5.70 | 1.38 | 3.20 | 7.59 | 1.72 | 4.00 | 9.49 | 2.07 | 4.79 | 11.39 | 2.41 | 5.59 | 13.29 | 2.75 | 6.39 | 15.19 | 3.10 | 7.19 | 17.09 | 3.44 | 7.99 | 18.99 |
|  | **3-<6 years** | 0.26 | 0.59 | 1.41 | 0.51 | 1.19 | 2.82 | 0.77 | 1.78 | 4.23 | 1.02 | 2.37 | 5.63 | 1.28 | 2.96 | 7.04 | 1.53 | 3.56 | 8.45 | 1.79 | 4.15 | 9.86 | 2.04 | 4.74 | 11.27 | 2.30 | 5.34 | 12.68 | 2.55 | 5.93 | 14.09 |
|  | **6-<11 years** | 0.15 | 0.35 | 0.82 | 0.30 | 0.69 | 1.65 | 0.45 | 1.04 | 2.47 | 0.60 | 1.39 | 3.30 | 0.75 | 1.73 | 4.12 | 0.90 | 2.08 | 4.94 | 1.05 | 2.43 | 5.77 | 1.20 | 2.77 | 6.59 | 1.34 | 3.12 | 7.42 | 1.49 | 3.47 | 8.24 |
|  | **11-<16 years** | 0.08 | 0.19 | 0.46 | 0.17 | 0.39 | 0.92 | 0.25 | 0.58 | 1.38 | 0.33 | 0.78 | 1.85 | 0.42 | 0.97 | 2.31 | 0.50 | 1.16 | 2.77 | 0.59 | 1.36 | 3.23 | 0.67 | 1.55 | 3.69 | 0.75 | 1.75 | 4.15 | 0.84 | 1.94 | 4.61 |
|  | **16-<21 years** | 0.07 | 0.15 | 0.37 | 0.13 | 0.31 | 0.73 | 0.20 | 0.46 | 1.10 | 0.27 | 0.62 | 1.46 | 0.33 | 0.77 | 1.83 | 0.40 | 0.92 | 2.20 | 0.46 | 1.08 | 2.56 | 0.53 | 1.23 | 2.93 | 0.60 | 1.39 | 3.29 | 0.66 | 1.54 | 3.66 |
|  | **adult** | 0.06 | 0.14 | 0.33 | 0.12 | 0.28 | 0.66 | 0.18 | 0.41 | 0.98 | 0.24 | 0.55 | 1.31 | 0.30 | 0.69 | 1.64 | 0.36 | 0.83 | 1.97 | 0.42 | 0.96 | 2.29 | 0.48 | 1.10 | 2.62 | 0.53 | 1.24 | 2.95 | 0.59 | 1.38 | 3.28 |
| **peanuts + cashews + hazelnuts + walnuts** | **6-11 months** | 0.69 | 1.38 | 3.07 | 1.38 | 2.75 | 6.13 | 2.07 | 4.13 | 9.20 | 2.76 | 5.51 | 12.27 | 3.45 | 6.88 | 15.33 | 4.14 | 8.26 | 18.40 | 4.83 | 9.64 | 21.47 | 5.52 | 11.01 | 24.53 | 6.20 | 12.39 | 27.60 | 6.89 | 13.77 | 30.67 |
|  | **1-<2 years** | 0.56 | 1.11 | 2.47 | 1.11 | 2.22 | 4.95 | 1.67 | 3.33 | 7.42 | 2.23 | 4.44 | 9.90 | 2.78 | 5.55 | 12.37 | 3.34 | 6.67 | 14.85 | 3.89 | 7.78 | 17.32 | 4.45 | 8.89 | 19.80 | 5.01 | 10.00 | 22.27 | 5.56 | 11.11 | 24.75 |
|  | **2-<3 years** | 0.46 | 0.92 | 2.04 | 0.92 | 1.84 | 4.09 | 1.38 | 2.75 | 6.13 | 1.84 | 3.67 | 8.18 | 2.30 | 4.59 | 10.22 | 2.76 | 5.51 | 12.27 | 3.22 | 6.42 | 14.31 | 3.68 | 7.34 | 16.35 | 4.14 | 8.26 | 18.40 | 4.60 | 9.18 | 20.44 |
|  | **3-<6 years** | 0.34 | 0.68 | 1.52 | 0.68 | 1.36 | 3.03 | 1.02 | 2.04 | 4.55 | 1.36 | 2.72 | 6.07 | 1.71 | 3.40 | 7.58 | 2.05 | 4.09 | 9.10 | 2.39 | 4.77 | 10.62 | 2.73 | 5.45 | 12.13 | 3.07 | 6.13 | 13.65 | 3.41 | 6.81 | 15.17 |
|  | **6-<11 years** | 0.20 | 0.40 | 0.89 | 0.40 | 0.80 | 1.77 | 0.60 | 1.19 | 2.66 | 0.80 | 1.59 | 3.55 | 1.00 | 1.99 | 4.44 | 1.20 | 2.39 | 5.32 | 1.40 | 2.79 | 6.21 | 1.60 | 3.19 | 7.10 | 1.80 | 3.58 | 7.98 | 1.99 | 3.98 | 8.87 |
|  | **11-<16 years** | 0.11 | 0.22 | 0.50 | 0.22 | 0.45 | 0.99 | 0.33 | 0.67 | 1.49 | 0.45 | 0.89 | 1.99 | 0.56 | 1.11 | 2.48 | 0.67 | 1.34 | 2.98 | 0.78 | 1.56 | 3.48 | 0.89 | 1.78 | 3.97 | 1.00 | 2.01 | 4.47 | 1.12 | 2.23 | 4.97 |
|  | **16-<21 years** | 0.09 | 0.18 | 0.39 | 0.18 | 0.35 | 0.79 | 0.27 | 0.53 | 1.18 | 0.35 | 0.71 | 1.58 | 0.44 | 0.88 | 1.97 | 0.53 | 1.06 | 2.36 | 0.62 | 1.24 | 2.76 | 0.71 | 1.42 | 3.15 | 0.80 | 1.59 | 3.55 | 0.89 | 1.77 | 3.94 |
|  | **adult** | 0.08 | 0.16 | 0.35 | 0.16 | 0.32 | 0.71 | 0.24 | 0.47 | 1.06 | 0.32 | 0.63 | 1.41 | 0.40 | 0.79 | 1.76 | 0.48 | 0.95 | 2.12 | 0.55 | 1.11 | 2.47 | 0.63 | 1.27 | 2.82 | 0.71 | 1.42 | 3.17 | 0.79 | 1.58 | 3.53 |
| **almonds + cashews + hazelnuts + walnuts** | **6-11 months** | 0.67 | 1.22 | 2.50 | 1.35 | 2.43 | 4.99 | 2.02 | 3.65 | 7.49 | 2.70 | 4.87 | 9.99 | 3.37 | 6.09 | 12.49 | 4.05 | 7.30 | 14.98 | 4.72 | 8.52 | 17.48 | 5.40 | 9.74 | 19.98 | 6.07 | 10.95 | 22.47 | 6.74 | 12.17 | 24.97 |
|  | **1-<2 years** | 0.54 | 0.98 | 2.02 | 1.09 | 1.96 | 4.03 | 1.63 | 2.95 | 6.05 | 2.18 | 3.93 | 8.06 | 2.72 | 4.91 | 10.08 | 3.27 | 5.89 | 12.09 | 3.81 | 6.88 | 14.11 | 4.35 | 7.86 | 16.12 | 4.90 | 8.84 | 18.14 | 5.44 | 9.82 | 20.15 |
|  | **2-<3 years** | 0.45 | 0.81 | 1.66 | 0.90 | 1.62 | 3.33 | 1.35 | 2.43 | 4.99 | 1.80 | 3.25 | 6.66 | 2.25 | 4.06 | 8.32 | 2.70 | 4.87 | 9.99 | 3.15 | 5.68 | 11.65 | 3.60 | 6.49 | 13.32 | 4.05 | 7.30 | 14.98 | 4.50 | 8.11 | 16.65 |
|  | **3-<6 years** | 0.33 | 0.60 | 1.24 | 0.67 | 1.20 | 2.47 | 1.00 | 1.81 | 3.71 | 1.33 | 2.41 | 4.94 | 1.67 | 3.01 | 6.18 | 2.00 | 3.61 | 7.41 | 2.33 | 4.21 | 8.65 | 2.67 | 4.82 | 9.88 | 3.00 | 5.42 | 11.12 | 3.34 | 6.02 | 12.35 |
|  | **6-<11 years** | 0.20 | 0.35 | 0.72 | 0.39 | 0.70 | 1.44 | 0.59 | 1.06 | 2.17 | 0.78 | 1.41 | 2.89 | 0.98 | 1.76 | 3.61 | 1.17 | 2.11 | 4.33 | 1.37 | 2.46 | 5.06 | 1.56 | 2.82 | 5.78 | 1.76 | 3.17 | 6.50 | 1.95 | 3.52 | 7.22 |
|  | **11-<16 years** | 0.11 | 0.20 | 0.40 | 0.22 | 0.39 | 0.81 | 0.33 | 0.59 | 1.21 | 0.44 | 0.79 | 1.62 | 0.55 | 0.99 | 2.02 | 0.66 | 1.18 | 2.43 | 0.76 | 1.38 | 2.83 | 0.87 | 1.58 | 3.24 | 0.98 | 1.77 | 3.64 | 1.09 | 1.97 | 4.04 |
|  | **16-<21 years** | 0.09 | 0.16 | 0.32 | 0.17 | 0.31 | 0.64 | 0.26 | 0.47 | 0.96 | 0.35 | 0.63 | 1.28 | 0.43 | 0.78 | 1.60 | 0.52 | 0.94 | 1.93 | 0.61 | 1.09 | 2.25 | 0.69 | 1.25 | 2.57 | 0.78 | 1.41 | 2.89 | 0.87 | 1.56 | 3.21 |
|  | **adult** | 0.08 | 0.14 | 0.29 | 0.16 | 0.28 | 0.57 | 0.23 | 0.42 | 0.86 | 0.31 | 0.56 | 1.15 | 0.39 | 0.70 | 1.44 | 0.47 | 0.84 | 1.72 | 0.54 | 0.98 | 2.01 | 0.62 | 1.12 | 2.30 | 0.70 | 1.26 | 2.58 | 0.78 | 1.40 | 2.87 |
